# A Deep Learning Approach to Analyzing Continuous-Time Cognitive Processes

**DOI:** 10.1162/opmi_a_00126

**Published:** 2024-03-13

**Authors:** Cory Shain, William Schuler

**Affiliations:** Department of Brain & Cognitive Sciences, Massachusetts Institute of Technology, Cambridge, MA, USA; Department of Linguistics, The Ohio State University, Columbus, OH, USA

**Keywords:** deep learning, time series, data analysis, nonlinear regression, human language processing

## Abstract

The dynamics of the mind are complex. Mental processes unfold continuously in time and may be sensitive to a myriad of interacting variables, especially in naturalistic settings. But statistical models used to analyze data from cognitive experiments often assume simplistic dynamics. Recent advances in deep learning have yielded startling improvements to simulations of dynamical cognitive processes, including speech comprehension, visual perception, and goal-directed behavior. But due to poor interpretability, deep learning is generally not used for scientific analysis. Here, we bridge this gap by showing that deep learning can be used, not just to imitate, but to *analyze* complex processes, providing flexible function approximation while preserving interpretability. To do so, we define and implement a nonlinear regression model in which the probability distribution over the response variable is parameterized by convolving the history of predictors over time using an artificial neural network, thereby allowing the shape and continuous temporal extent of effects to be inferred directly from time series data. Our approach relaxes standard simplifying assumptions (e.g., linearity, stationarity, and homoscedasticity) that are implausible for many cognitive processes and may critically affect the interpretation of data. We demonstrate substantial improvements on behavioral and neuroimaging data from the language processing domain, and we show that our model enables discovery of novel patterns in exploratory analyses, controls for diverse confounds in confirmatory analyses, and opens up research questions in cognitive (neuro)science that are otherwise hard to study.

## INTRODUCTION

The human brain is the most sophisticated computing device known, and one of the least understood. In the course of daily life it solves a wide array of difficult inference problems concurrently and with extraordinary efficiency. Psychologists, neuroscientists, and cognitive scientists seek to understand the real-time cognitive processes that underlie these abilities, and they are increasingly interested in studying these processes in context using naturalistic stimuli, tasks, and environments (Campbell & Tyler, 2018; Hamilton & Huth, 2018; Hasson et al., 2010, 2018). Naturalistic experiments typically involve observational (rather than experimental) designs that shift the burden of experimenter effort from experimental control to analysis: critical variables must be appropriately coded, control variables must adequately cover plausible confounds, and statistical models must be sufficiently expressive to capture the underlying characteristics of the experimental measure, whether behavioral or neural.

In practice, the statistical models used to analyze observational time series are overwhelmingly based on linear regression or generalizations thereof (Bates et al., 2015; Sims, 1980). These approaches make the following simplifying assumptions in some combination: time passes in discrete steps, effects are linear, and the response^1^ is stationary (time-invariant) and homoscedastic (constant variance). Are these assumptions always appropriate for the study of the mind and brain? One reason for skepticism comes from the machine learning literature. By far the greatest progress in artificially simulating human cognitive abilities has come from highly expressive, interactive, and nonlinear deep neural network (DNN) models (LeCun et al., 2015), which show a remarkable capacity to mimic dynamical cognitive processes like speech comprehension (Graves et al., 2013) and production (van den Oord et al., 2016), visual perception (Gao et al., 2017), and goal-directed behavior (Schrittwieser et al., 2020) in uncontrolled, naturalistic settings. These gains come from DNNs’ ability to flexibly integrate multiple sources of information, discover hidden structure, and adapt computations to relevant aspects of context (LeCun et al., 2015), abilities which are absent from standard regression analyses but are plausibly present in the human cognitive processes that scientists want to understand and that DNNs successfully emulate. Nevertheless, DNNs are rarely used for scientific data analysis because they are “black boxes”: they can accurately map inputs to outputs, but the computations they use to do so are opaque and therefore tend to be of limited value for understanding the modeled system.

These challenges are perhaps especially pronounced in the study of language processing, where the mind is managing a large space of variables (including world knowledge; episodic memory; semantic, syntactic, lexical, and phonological structure; perception; and articulation), each with myriad structural and statistical relationships to aspects of the local context (Ehrlich & Rayner, 1981; Frank & Goodman, 2012; Gibson, 2001; Hale, 2001; Taylor, 1953; Nicol & Swinney, 1989; Lewis & Vasishth, 2005; Warren, 1970). Furthermore, this complex processing is carried out so rapidly and incrementally that responses to multiple parts of the stimulus sequence (e.g., words) likely overlap in time (Mitchell, 1984; Smith & Kutas, 2015; Shain & Schuler, 2021); this overlap can be substantially increased by measurement latencies (e.g., in hemodynamic measures of brain activity; Boynton et al., 1996), beyond any latencies at the neuronal level. In addition, measures of language processing can change moment-by-moment due to e.g., task habituation, attentional fluctuation, and fatigue (Baayen et al., 2018; Christianson et al., 2022; Prasad & Linzen, 2021). Thus, studies of language processing might be particularly ill served by analyses that rely on stationary linear models: poor model fit to the underlying dynamics can both lead to misleading inferences and limit the range of questions that can be investigated (e.g., Baayen et al., 2018; Shain & Schuler 2021; cf. Thul et al., 2021).

Here we show that an appropriate combination of DNN design and black box interpretation can overcome this issue, synthesizing the flexibility of deep learning with the interpretability of linear regression. Our approach—the continuous-time deconvolutional regressive neural network (CDR-NN)—uses deep learning to relax the key simplifying assumptions above (discrete time, linearity, stationarity, and homoscedasticity) in order to estimate, visualize, and test properties of the response structure of a complex process from data. Our study expands significantly upon an earlier proposal of the CDR-NN approach (Shain, 2021, see SI A for detailed comparison). We evaluate CDR-NNs on a range of synthetic data, as well as on publicly available behavioral and neural data from studies of human language processing. We show that CDR-NNs yield large improvements to out-of-sample model fit over alternative approaches on both behavioral and neuroimaging data. We further show that CDR-NNs enable both flexible discovery of novel structure in exploratory analyses and control of diverse potential confounds in confirmatory analyses, and thus constitute an important advance for both goals.

## RELATED METHODS FOR ANALYZING TIME SERIES

Here we briefly review existing approaches to analyzing observational time series and discuss key simplifying assumptions (which can be relaxed by CDR-NNs) that are made in some combination by each of them: discrete time, linearity, stationarity, and homoscedasticity.

In regression analyses of observational time series, linear models (LMs) are currently the dominant method. Linear regression attempts to identify the vector of parameters **b** that models the expected value of response *y* via linear combination with predictor vector **x**:

$$\text{E}\left(y\right)=x^{⊤}b$$ (1)

A common way of relaxing this linearity assumption is the *generalized additive model* (GAM; Hastie & Tibshirani, 1986; Wood, 2006), which permits arbitrary nonlinear spline functions *f*_*k*_ on subsets of predictors **v**_1_, …, **v**_*k*_ ∈ 𝒫(**x**) (vectors corresponding to the powerset of elements in **x**):

$$\text{E}\left(y\right)=f_{1}\left(v_{1}\right)+\ldots +f_{k}\left(v_{k}\right)$$ (2)

Both linear and GAM regression models can be augmented with random effects terms to capture hierarchical structure in the observations (Bates et al., 2015; Wang, 1998). Errors in these models are assumed to be independent and identically distributed, and normal error is commonly assumed for continuous response variables. This entails that variance is assumed constant (homoscedastic), since only the expectation E(*y*) (and not any other distributional parameter) is modeled as a function of **x**. This assumption can relaxed using generalized additive models for location, scale and shape (GAMLSS; Rigby & Stasinopoulos, 2005), a generalization of GAMs that admits additive nonlinear influences of predictors on up to four parameters of the distribution over the response (response distribution) 𝓕 with parameter vector **s** over response *y*:

$$s=f_{1}\left(v_{1}\right)+\ldots +f_{k}\left(v_{k}\right)$$ (3)

$$y∼𝓕\left(s\right)$$ (4)

When naively applied to time series, these approaches make strong temporal independence assumptions: the response *y*_*i*_ depends solely on the corresponding predictors **x**_*i*_ and is independent of any predictor values that precede (or follow) *y*_*i*_ in time. This assumption can be relaxed in the design of **x** e.g., by including regressors from previous events—yielding a *distributed lag* (Koyck, 1954) or *finite impulse response* (FIR; Neuvo et al., 1984) model (also called “spillover” in psycholinguistics; Mitchell, 1984)—or by including variables encoding the passage of time, which are especially useful in GAMs to permit modeling of nonstationarity (Baayen et al., 2018). Relatedly, when the response variable has sufficiently high temporal resolution relative to the predictors, the time series can be “epoched” by fitting a separate linear model for each of a set of fixed delays relative to the predictor timestamps. This approach is commonly used to estimate impulse response functions (e.g., event-related potentials) in electrophysiology (e.g., Smith & Kutas, 2015). A similar kind of impulse response estimation is possible using vector autoregression (Sims, 1980) and related methods (e.g., reservoir computers; Bollt, 2021; Gauthier et al., 2021), which infer the timecourse of evoked changes in a dynamical system provided some discretization of the time dimension into steps. However, as argued at length in Shain and Schuler (2021), discrete-time approaches like FIR, epoching, and vector autoregression are limited in their ability to infer continuous dynamics from data with variable event durations and low temporal resolution relative to the predictors, and are thus difficult to apply to many kinds of time series.

In response to this limitation, Shain and Schuler (2021) proposed *continuous-time deconvolutional regression* (CDR), a kernel-based variational Bayesian model that infers the parameterization of continuous-time impulse response functions (IRFs) from data. In brief, in CDR, the expected value of *y*_*t*_ at timestamp *t* is a linear model on $x_{t}^{\prime }$, where $x_{t}^{\prime }$ is a convolution over time of preceding inputs *x*(*t*) with convolution weights derived from the estimated IRF *g*(*t*):

$$\text{E}\left(y_{t}\right)={\left(x_{t}^{\prime }\right)}^{⊤}b$$ (5)

$$x_{t}^{\prime }=\int_{0}^{t}x\left(\tau \right)g\left(t-\tau \right)d\tau$$ (6)

CDR otherwise assumes homoscedasticity and stationarity (like LMs and GAMs) and linear/additive effects (like LMs). The IRFs estimated by CDR describe diffusion of effects over time in continuous-time dynamical systems (like the human mind) in which previous events may continue to influence the response as the experiment unfolds. CDR substantially improves fit to naturalistic human language processing data, while also shedding light on important aspects of processing dynamics that are otherwise difficult to obtain (Shain & Schuler, 2021). For in-depth review of these and related approaches to time series analysis, especially under the possibility of delayed effects, see Shain and Schuler (2021).

CDR-NNs relax all of the above assumptions using *deep learning*—the use of multilayer artificial neural networks for function approximation (LeCun et al., 2015). An artificial neural network is a supervised machine learning algorithm that transforms inputs into outputs via nonlinear transformations with learned parameters. A *deep* neural network (DNN) involves sequential transformations of the network’s own hidden states, allowing the network to learn complex nonlinear interactions of the input features. DNNs have been shown by mathematical analyses to be universal function approximators (Hornik, 1991), and thousands of practical applications have demonstrated their effectiveness for learning complex patterns in real data, to the point that DNNs now dominate engineering fields like natural language processing and computer vision (LeCun et al., 2015). A DNN is typically trained by *backpropagation* (Rumelhart et al., 1986), which involves (1) computing the partial derivatives of some objective function (e.g., negative log likelihood) with respect to each of the model’s parameters and (2) changing those parameters via a deterministic function of the computed derivatives, seeking to optimize the objective. CDR-NNs relate the predictors **x** to the probability distribution over response *y* using deep neural networks whose architecture ensures continuous-time deconvolution (see The CDR-NN Model below). As in CDR (Shain & Schuler, 2021), a CDR-NN is a time series model that estimates a function relating two arbitrary-length time series (predictors and responses) via convolution over time, with no “epoching” of response data relative to predictor timestamps (cf., common practice in electrophysiology; Smith & Kutas, 2015). This design allows CDR-NNs to apply to a wider range of time series, including those in which the temporal resolution of the response is low relative to the predictors (e.g., behavioral or functional magnetic resonance imaging—fMRI—studies of many cognitive processes).

The consequences of model definitions for the kinds of information about the underlying process that can and cannot be captured by a given model type are summarized in Table 1. LMs, GAMs, and GAMLSS can only model discrete-time IRFs, whereas CDR and CDR-NNs can additionally model continuous-time IRFs. LMs and CDR only model linear effects, whereas GAMs, GAMLSS, and CDR-NNs also model nonlinear effects. LMs and CDR only model linear effect interactions that are sparse (i.e., specified by the analyst), whereas GAMs and GAMLSS also model nonlinear interactions through sparse tensor-product spline functions. Only CDR-NNs model arbitrary nonlinear interactions over the full set of predictors, while also permitting explicit constraints on interactions and nonlinearity under an appropriate model definition. LMs and CDR only capture nonstationarity in the form of linear trends over time, whereas GAMs, GAMLSS, and CDR-NNs capture arbitrary nonstationarity via interactions with the time dimension. Finally, only GAMLSS and CDR-NNs directly model influences of predictors on all parameters of the response distribution (distributional regression), and thereby capture heteroscedasticity in the modeled system. CDR-NNs therefore merge the advantages of continuous-time deconvolutional modeling from CDR with the advantages of nonlinear modeling from GAM(LSS), at least as implemented in popular software packages like lme4 (Bates et al., 2015) and mgcv (Wood, 2006)—see e.g., Bürkner (2018) for methods to relax linearity and homoscedasticity assumptions using Bayesian inference.

**Table 1.** Comparison of key features of the solution spaces defined by linear models (LMs), generalized additive models (GAMs), generalized additive models for location, scale, and shape (GAMLSS), continuous-time deconvolutional regression (CDR), and continuous-time deconvolutional regressive neural networks (CDR-NNs). The sparse/dense distinction under *Interactions* concerns whether analysts must explicitly add interactions to the model (sparse) or whether the model considers all possible interactions (dense). Features absent from a model type cannot be directly modeled when using it.

	Feature	LM	GAM	GAMLSS	CDR	CDR-NN
Impulse response	Discrete-time	✓	✓	✓	✓	✓
	Continuous-time	·	·	·	✓	✓
Effects	Linear	✓	✓	✓	✓	✓
	Nonlinear	·	✓	✓	·	✓
Interactions	Linear sparse	✓	✓	✓	✓	✓
	Nonlinear sparse	·	✓	✓	·	✓
	Nonlinear dense	·	·	·	·	✓
Nonstationarity	Linear	✓	✓	✓	✓	✓
	Nonlinear	·	✓	✓	·	✓
Predictive distribution	Homoscedastic	✓	✓	✓	✓	✓
	Heteroscedastic	·	·	✓	·	✓

CDR-NNs bear a close conceptual relationship to multiple recent toolkits that build either on the LM/GAM frameworks reviewed above or on deep learning. For example, the multivariate temporal response function (mTRF) toolbox (Crosse et al., 2016) supports regularized linear modeling for impulse response identification. Nonlinear generalizations of this idea have been developed using generalized additive models (Ehinger & Dimigen, 2019) and recurrent neural networks (Chehab et al., 2022). These approaches are all underlyingly discrete-time in that they assume a regular sampling interval for the response variable to which the stimulus sequence must be aligned. For high temporal resolution measures like EEG and MEG in which the sampling interval is both regular and fast compared to the stimulus stream, this assumption is appropriate. However, it becomes problematic when events have variable duration and the sampling density of the response is low relative to the stimulus, as in language experiments using reading times or fMRI (Shain & Schuler, 2021). Thus, in addition to their advantages for capturing complexities like nonlinearity, interactions, non-stationarity, and heteroscedasticity, CDR-NNs can be applied to a broader range of domains than existing tools.

See Why Modeling Assumptions Matter: The Case of Human Language Processing for discussion of how the specific simplifying assumptions summarized in Table 1 are potentially problematic for the domain of language processing.

## THE CDR-NN MODEL

Our core proposal (CDR-NN) is a deep neural generalization of several existing techniques for time series regression, including the linear models (LMs, Galton, 1886), linear mixed effects models (LMEs, Bates et al., 2015), generalized additive models (GAMs, Wood, 2006), generalized additive models for location, scale, and shape (GAMLSS, Rigby & Stasinopoulos, 2005), and continuous-time deconvolutional regression models (CDR, Shain & Schuler, 2021) discussed above.

We define the regression problem as follows (for a reference of variable definitions, see Table 2). Let **y** ∈ ℝ^*Y*^ be a single sample from the *Y*-dimensional dependent variable that we seek to model (the response, e.g., an fMRI blood oxygen level-dependent—BOLD—measure), taken at time *τ* (e.g., seconds elapsed between the start of the experiment and the acquisition time of the fMRI image). Let 𝓕 be a probability distribution with *S*-dimensional parameter vector **s** ∈ ℝ^*S*^ (e.g., the mean and variance of a normal distribution over the response) such that **y** ∼ 𝓕(**s**). Let **X** ∈ ℝ^*N*×*K*^ be a matrix of *N* *K*-dimensional predictor vectors **x**_*n*_, 1 ≤ *n* ≤ *N* (e.g., the duration and relative frequency of each of the *N* words in a story). Let **t** ∈ ℝ^*N*^> be the vector of predictor timestamps *t*_1_, …, *t*_*N*_ such that *t*_*n*_ is the timestamp of **x**_*n*_ (e.g., seconds elapsed between the start of the experiment and the onset of a word in a story). Let **d** ∈ ℝ^*N*^ be the vector of temporal offsets *d*_1_, …, *d*_*N*_ such that *d*_*n*_ = *τ* − *t*_*n*_, i.e., the signed distance in time between **y** and **x**_*n*_ (e.g., the time in seconds of an fMRI volume minus the time in seconds of the word onsets in a story). A CDR-NN defines a function from 〈**X**, **t**, *τ*〉 to **s**, that is, from the predictors and their timestamps to the parameters of the response distribution at a particular delay.

**Table 2.**
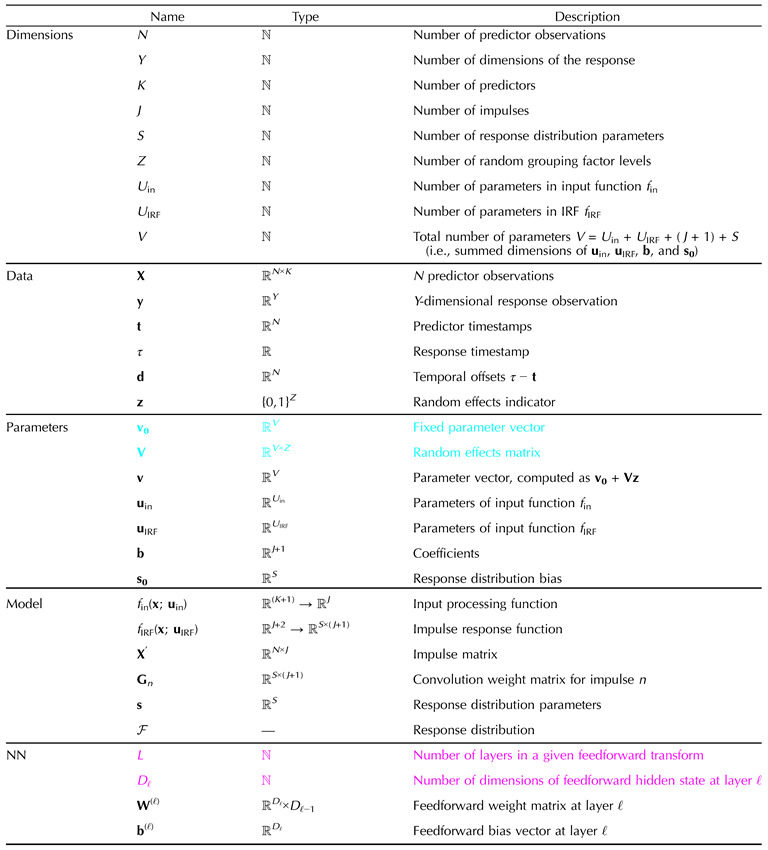
Summary of variables in CDR-NN model definition. Trainable parameters (**v**_**0**_ and **V**) are shown in 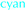. All other model quantities are inferred from these as described in the equations in The CDR-NN Model. Hyperparameters needed for the mathematical definition are shown in 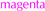. Implemented models require other hyperparameters (e.g., learning rate), as described in SI D.

The CDR-NN computation involves three stages (see SI B for detailed motivation and SI C for pseudocode). The first stage is **input processing**, which maps the sequence of predictors in the model into a sequence of *impulses* that may evoke changes in the parameters of the response distribution. Formally, the vector of timestamps **t** is horizontally concatenated with the predictor matrix **X** to form the input to the input processing function *f*_in_ ∈ ℝ^(*K*+1)^ → ℝ^*J*^ with parameters **u**_in_ ∈ ℝ^*U*_in_^. The output **X**′ ∈ ℝ^*N*×*J*^ is a matrix of *N* *J*-dimensional impulse vectors $x_{n}^{\prime }$, 1 ≤ *n* ≤ *N* computed independently by *f*_in_:

$$x_{n}^{\prime }\overset{\text{def}}{=}f_{\text{in}}\left(\left[\begin{array}{c} t_{n}\\ x_{n} \end{array}\right];u_{\text{in}}\right)$$ (7)

Note that *f*_in_ may simply return **x**_*n*_ unaltered, in which case there is no distinction between predictors and impulses (see below for discussion).

The second stage is the **impulse response function (IRF)**, which computes the expected change in the parameters of the response distribution due to each impulse in the time series, as a function of the properties of the impulse as well as its timing relative to the target response. Formally, the impulses **X**′ are horizontally concatenated with **d** and **t** to yield the inputs to IRF *f*_IRF_ ∈ ℝ^*J*+2^ → ℝ^*S*×(*J*+1)^ with parameters **u**_IRF_ ∈ ℝ^*U*_IRF_^. The additional dimension of the output (i.e., *J* + 1 rather than *J*) is included to support estimation of the base response of the system (*rate*, see Equation 9 and accompanying discussion below). The output of the IRF is a sequence of convolution weight matrices **G**_*n*_ ∈ ℝ^*S*×(*J*+1)^, 1 ≤ *n* ≤ *N*, where each **G**_*n*_ describes the effect of impulse $x_{n}^{\prime }$ (e.g., a word in a story) on the response distribution (e.g., a BOLD measure) at time *τ* (or, equivalently, at delay *d*_*n*_):

$$G_{n}\overset{\text{def}}{=}f_{\text{IRF}}\left(\left[\begin{array}{c} d_{n}\\ t_{n}\\ x_{n}^{\prime } \end{array}\right];u_{\text{IRF}}\right)$$ (8)

The third stage is **convolution**, which computes the expected overall change in the parameters of the response distribution as a function of its temporal context, by summing the individual influences of the impulses as computed by the IRF. Formally, the parameters **s** for the response distribution 𝓕 are computed as the sum of (i) the temporal convolution of **X**′ with **G**_1_, …, **G**_*N*_ and (ii) learned bias vector (intercept) **s**_**0**_, where each transposed row $x_{n}^{\prime }$, 1 ≤ *n* ≤ *N* of **X**′ is vertically concatenated with a bias. This bias, which we have called *rate* in prior work (Shain, 2021; Shain & Schuler, 2018, 2021), serves to capture general effects of stimulus timing, or, equivalently, the baseline response of the system to a stimulus, without regard to stimulus properties. *Rate* can therefore be regarded as a kind of “deconvolutional intercept”, i.e., a baseline response that is added to any stimulus-specific responses. *Rate* is distinct from the intercept **s**_**0**_ in that it is convolved with an impulse response. For extended discussion, see Shain and Schuler (2021). The IRF output **G**_*n*_ is weighted by learned coefficient vector **b** ∈ ℝ^*J*+1^, thus factoring the shape of the impulse response (**G**_*n*_) from its scale (**b**). This factorization enables flexible composition of deep neural and parametric response functions (e.g., linear terms or parametric kernels) within a single model:

$$s\overset{\text{def}}{=}s_{0}+\sum_{n=1}^{N}G_{n}\mathrm{diag}\left(b\right)\left[\begin{array}{c} 1\\ x_{n}^{\prime } \end{array}\right]$$ (9)

The impulses $x_{n}^{\prime }$ appear both in the inputs to the convolution weights **G**_*n*_ (Equation 8) and in the convolution itself (Equation 9) in order to allow **G**_*n*_ to be either nonlinear or linear on dimensions of $x_{n}^{\prime }$, depending on the goals of the analyst (see SI B for details).

Convolving over the entire predictor sequence, as in Equation 9, may seem to allow a causal influence of the future on the past, which motivates us to clarify two points in response. *First*, a CDR-NN is a regression model and thus not necessarily a model of causation. We seek a definition general enough to admit all relevant modeling problems, including unequivocally non-causal ones (e.g., reversing the directionality in order to decode stimulus features from the future response that they evoke). *Second*, temporal constraints (e.g., an arrow of time assumption) can be enforced when desired by the choice of *f*_IRF_ (e.g., setting its output to 0 when delay **d** is negative—indicating that the input is from the future).

Mixed effects CDR-NN models can be defined by letting the parameter vector **v** ∈ ℝ^*V*^:

$$v\overset{\text{def}}{=}\left[\begin{array}{c} u_{\text{in}}\\ u_{\text{IRF}}\\ b\\ s_{0} \end{array}\right]$$ (10)

be the sum of a fixed part **v**_**0**_ ∈ ℝ^*V*^ and random part **Vz**, where **V** ∈ ℝ^*V*×*Z*^ is a random effects matrix subject to shrinkage penalties (see SI D) whose rows sum to 0 and **z** ∈ {0, 1}^*Z*^ indicates which of *Z* random effects levels apply to **y** (**Vz** is thus mathematically equivalent to indexing and summing all applicable random deviations in the parameters for a given response):

$$v\overset{\text{def}}{=}v_{0}+Vz$$ (11)

Random effects **V** thus allow the model to capture sample-specific random deviation (for example, random deviation by participant), in any model parameter (or any subset of these parameters specified as random by the experimenter), including **s**_**0**_ (analogous to “random intercepts” from linear mixed models), **b** (analogous to “random slopes” from linear mixed models), and **u**_in_ and **u**_IRF_ (thus capturing e.g., random deviation in the IRF shape). This is intended to allow the fixed effects estimates **v**_**0**_ to better reflect central tendency in the population. The parameters of the model are therefore **v**_**0**_ and **V**, which may be fitted via maximum likelihood or Bayesian inference—the procedure for inferring **v**_**0**_ and **V** is orthogonal to the mathematical model definition; models in this study are fitted by variational Bayesian inference using a combination of variational expectation maximization (Tran et al., 2016) and Monte Carlo dropout (Gal & Ghahramani, 2016), as described in SI D. This definition assumes a singleton dataset 𝒟 = {<**X**, **t**, **y**, *τ*>}, but it extends without loss of generality to any finite dataset by applying Equation 9 independently to each of *M* elements in 𝒟 = {<**X**_*m*_, **t**_*m*_, **y**_*m*_, *τ*_*m*_>|1 ≤ *m* ≤ *M*}.

These equations generalize multiple existing time series models. If *f*_in_ is set to be identity and *f*_IRF_ is set to be a Dirac *δ* on **d**, the result is a linear model. If *f*_in_ is set to be a parametric spline function and *f*_IRF_ is set to be a Dirac *δ* on **d**, the result is a GAM. If, in addition, *f*_in_ has vector-valued output that defines all parameters of the response distribution, the result is a GAMLSS. If *f*_in_ is set to be identity and *f*_IRF_ is set to be a parametric kernel function, the result is a CDR model.

However, in this work, motivated by evidence that deep neural networks enable high-accuracy nonlinear function approximation across domains and tasks (LeCun et al., 2015), we focus on CDR-NNs, by which we mean any model that instantiates *f*_in_ or *f*_IRF_ as a deep neural network. Implementing CDR-NNs requires a novel neural network architecture. To see why, note that the convolution over time in Equation 9 imposes an important constraint on the regression problem, namely, that the contributions of *f*_IRF_ at timepoints 1 ≤ *n* ≤ *N* are *additive*. This constraint is central to CDR-NNs’ interpretability, since it allows *f*_IRF_ to define a valid impulse response function, such that evaluating **G**_*n*_ yields a complete description of the causal contribution of input *n* to the distribution 𝓕(**s**) generated by the model. Time series models widely used in deep learning—including recurrent neural networks (Elman, 1991), convolutional neural networks (LeCun et al., 1989), and transformers (Vaswani et al., 2017)—violate this constraint by integrating over time in a nonlinear fashion and therefore do not implement deconvolutional regression, continuous-time or otherwise.

**Figure 1.**
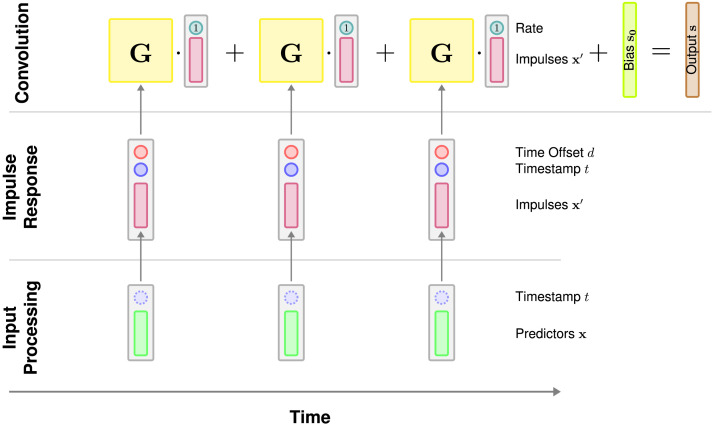
**CDR-NN architecture.** A grapical depiction of the CDR-NN forward pass for generating one prediction. Scalars are shown as circles, vectors are shown as narrow boxes, matrices are shown as wider boxes, and deep neural network transformations are shown as arrows. Computation proceeds in three stages (bottom-to-top): (i) processing the inputs, (ii) applying the impulse response, and (iii) convolving the impulses with the IRF (convolution weights) over time to generate a parameterization for the predictive distribution over the response. At the convolution stage, the impulses are augmented with bias term (*rate*) that allows the model to capture generalized effects of the rate of events in time. To reduce visual clutter, yellow matrices labeled **G** stand in for the product **G**_*n*_ diag (**b**) of Equation 9. Components shown with dotted lines are not used in the base CDR-NN implementation in this study (although they are explored in the full set of analyses, see SI).

Our proposed CDR-NN architecture is schematized in Figure 1, where arrows represent transformations implemented by a *feedforward neural network* (FFN). An FFN *f*_FF_ with *L* layers contains weights **W**^(ℓ)^ ∈ ℝ^*D*_ℓ_×*D*_ℓ−1_^ (where *D*_0_ = *K*), biases **b**^(ℓ)^ ∈ ℝ^*D*_ℓ_^ and activation functions *σ*^(ℓ)^, 1 ≤ ℓ ≤ *L*, and is defined recursively as follows (where $f_{\text{FF}}^{\left(0\right)}$(**x**) = **x**, the input vector):

$$f_{\text{FF}}^{\left(\ell \right)}\left(x\right)\overset{\text{def}}{=}\sigma^{\left(\ell \right)}\left(W^{\left(\ell \right)}f_{\text{FF}}^{\left(\ell -1\right)}\left(x\right)+b^{\left(\ell \right)}\right)$$ (12)

As shown in Figure 1, at each timepoint, our CDR-NN transforms the predictor vectors **x**_*n*_ (green) along with their timestamps *t*_*n*_ (blue) using an FFN implementing *f*_in_ (input processing). The outputs $x_{n}^{\prime }$ (red) are then concatenated with their timestamps *t*_*n*_ and their signed offset in time **d**_*n*_ from the prediction target and processed by a second FFN implementing *f*_IRF_ (impulse response). The IRF output **G**_*n*_ diag (**b**) of Equation 9, simplified in the figure as **G** (yellow), is a matrix of convolution weights mapping $x_{n}^{\prime }$ and a bias term (*rate*) to the estimated contribution of timepoint *n* to the response distribution. These estimated contributions are summed together with a bias term (an intercept **s**_**0**_, lime) to yield the output of the CDR-NN: a vector **s** (brown) of parameters for the distribution 𝓕 over a single response measure at a point in time (convolution). In our implementation, the parameters of the model are estimated using stochastic gradient descent, subject to standard deep neural network regularizers (e.g., weight penalties and dropout, see SI D).

Note that there is little reason in principle for both *f*_in_ and *f*_IRF_ to be deep neural networks in the general case, since effect nonlinearities and interactions can be directly estimated by *f*_IRF_. The primary interest of a deep neural *f*_in_ is to allow GAM-like estimation of effect nonlinearities and interactions when the impulses **X**′ are absent from the inputs to *f*_IRF_, thus decoupling the shape of effects in predictor space and the shape of the response in time. For this reason, in all analyses conducted in this study, *f*_in_ is assumed to be identity.

This simple network respects the constraints imposed by Equation 9 while also relaxing the key simplifying assumptions in Table 1. In particular, this network (i) relaxes the discrete-time assumption by modeling a continuous IRF via the dependence on **d**_*n*_, (ii) relaxes the stationarity assumption via the dependence on **t**_*n*_, (iii) relaxes the homoscedasticity assumption by parameterizing the entire distribution 𝓕(**s**) as a function of the inputs, and (iv) captures arbitrary nonlinearities and effect interactions by processing the entire predictor vector with an FFN. Unlike some related models of dynamical systems (e.g., vector autoregression, reservoir computers, and recurrent neural networks), CDR-NNs as defined above are stateless, modeling the system output solely by summing over the independent contributions of the inputs, without reference to a system state at a point in time. This property not only makes CDR-NNs more parallelizable (and thus, more computationally efficient) than stateful approaches, but it also allows the estimated IRF to be queried exactly, which is a key advantage for interpretability, as discussed above. However, this property also limits the model’s ability to integrate nonlinearly over context, which may be too constraining for some modeling problems. To address this limitation at some expense to interpretability, in SI E, we additionally propose a generalization of this model that uses recurrence to capture non-independence between inputs in their effects on the response.

As deep neural networks, CDR-NNs lack a transparent link between the values of parameters and their effects on the response. This presents a challenge for effect estimation, which is of critical interest in scientific applications. To address this, we employ a technique from deep learning known as *perturbation analysis* (Petsiuk et al., 2018; Ribeiro et al., 2016). In brief, we quantify the effect on network outputs of manipulating network inputs, permitting analysis of the network’s latent IRF. Full technical details about CDR-NN effect estimation and uncertainty quantification can be found in SI F. A documented software library for CDR-NN regression is available at https://github.com/coryshain/cdr.

## WHY MODELING ASSUMPTIONS MATTER: THE CASE OF HUMAN LANGUAGE PROCESSING

We have so far elaborated on the simplifying assumptions (discrete time dynamics, linearity, additivity, stationarity, and homoscedasticity) that are implicit in standard time series analysis methods in cognitive science (Table 1) and proposed a mathematical model of how these assumptions can be relaxed (Equation 9). But do these assumptions matter in practice? Should researchers care which assumptions are implicit in their analyses? In this section, we further motivate our proposal by reviewing prior reasons to think that each of these assumptions is systematically violated by human-generated data, at least for the domain of human language processing (see also Results for direct evidence that assumptions can affect statistical tests).

### Assumption: Discrete-Time Impulse Response

The impulse response functions within the solution spaces of LMs, GAMs, and GAMLSS are defined in discrete time. Delayed effects must be captured by some fixed number of lagged regressors to preceding events. There is a core difficulty in applying these models to time series generated by an underlyingly continuous system responding to variably spaced events: the discrete structure forces an indexical rather than continuous notion of time. Methods of coercing the model and/or data are needed in order to align the lags with preceding events, which either destroys temporal information or compromises model identifiability (for further discussion, see Shain & Schuler, 2021). CDR(NN) relaxes this assumption by permitting a continuous impulse response in the form of continuous kernels with trainable parameters.

Delayed effects are ubiquitous in human language comprehension (Kutas & Hillyard, 1980; Mitchell, 1984; Shain & Schuler, 2021; Smith & Levy, 2013; Van Dyke, 2007) and prior evidence indicates that this discrete-time assumption may be ill-suited to capture them. In particular, multiple lines of evidence indicate that, for diverse processing phenomena, the key determinant of delayed effects is how long ago the trigger word occurred *in time*, rather than how many words back it occurred. A large electrophysiological literature on human language processing investigates event-related potentials (ERPs, in electroencephalography, EEG) or event-related fields (ERFs, in magnetoencephalography, MEG), that is, IRFs that characterize the brain response to words in context. ERPs are described by their average peak delay in ms, such as the N400 (a negative deflection occurring around 400 ms after word onset) and the P600 (a positive deflection occurring around 600 ms after word onset). Studies consistently find effects consistent with well-known stereotyped clock-time ERPs in response to phonological (Connolly & Phillips, 1994; Kaan et al., 2007), morphological (Allen et al., 2003; Osterhout & Mobley, 1995), syntactic (Ainsworth-Darnell et al., 1998; Osterhout & Holcomb, 1992), and semantic (Kutas & Hillyard, 1980; Van Berkum et al., 1999) aspects of language, despite variable word presentation rates across experiments. This suggests that the relevant cognitive processes unfold in continuous time, rather than indexically (word-by-word).

Related work suggests that the human language processor may allow information processing to lag behind perception when processing load spikes (Bouma & De Voogd, 1974; Erlich & Rayner, 1983; Kliegl et al., 2006; Mollica & Piantadosi, 2017). If these lags are driven by rate-limited processing (Mollica & Piantadosi, 2017), this entails that the processing mechanisms that underlie them unfold in continuous time, rather than e.g., delaying processing until the next word is encountered, as implied by discrete-time models of reading behavior.

Evidence not only indicates that effect delays in human language comprehension are largely continuous-time rather than discrete-time, but also that discrete-time approximations to them are likely often poor quality due to extensive variability in word duration in natural language, whether spoken (Baker & Bradlow, 2009; Demberg et al., 2012) or read (Frank et al., 2013; Futrell et al., 2021). There is likely a substantial difference in the level of influence exerted by the preceding word depending on whether it occurred 100 ms vs. 1000 ms ago, a difference which is ignored by discrete-time models. Relaxing the discrete-time assumption using CDR leads to substantial improvements to model fit in reading and neuroimaging measures of human language processing relative to comparable discrete-time controls (Shain & Schuler, 2021), suggesting that these controls lack access to critical information about underlyingly continuous comprehension processes.^2^

### Assumption: Additive Linear Effects

LMs, GAMs, GAMLSS, and CDR all model the response as a weighted sum of the predictors, and LMs and CDR additionally assume that these weights scale linearly on the predictors (GAMs relax the latter assumption by deriving weights through nonlinear spline functions). The linearity assumptions of LMs and CDR can be problematic for model interpretation (the best-fit line may be a poor fit to a nonlinear function), and may prevent the discovery of theoretically-relevant nonlinearities.

Predictor interactions are subject to the same constraints, and can only be modeled if explicitly included by the analyst. As a result, each of these models has the following two properties:

1. *Correlated predictors are in zero-sum competition*. Increasing the effect of one covariate requires a corresponding decrease in the effect of the other. In cases of sufficiently high correlation, this can result in large-magnitude estimates of opposite sign (Wurm & Fisicaro, 2014), which are difficult to interpret.
2. *Interactions must be anticipated in advance*. This is of course a practical constraint: any LM or GAM could in principle include all possible interactions, and GAMs can further include multivariate spline functions of the full set of predictors. However, beyond a small handful of predictors, these approaches quickly produce problems for inference and computation due to combinatorial explosion. Analysts are therefore typically constrained by model identifiability considerations to a include small subset of interactions of interest based on prior evidence or other domain knowledge.

These two properties can be problematic for natural processes, which often involve many correlated and potentially interacting variables. They also prevent flexible inference of unanticipated interactions, which could serve as the basis for new discoveries.

The functional form and interaction structure of effects are of great interest to key questions in the study of human language processing. For example, one prominent debate concerns the functional form of predictability effects in reading (Brothers & Kuperberg, 2021; Levy & Jaeger, 2007; Smith & Levy, 2013; Wilcox et al., 2020), which has implications for extant theories of human language processing (Smith & Levy, 2013). Another debate concerns the existence of an interaction between word frequency and word predictability effects on incremental language comprehension effort (Ashby et al., 2005; Kretzschmar et al., 2015; Rayner et al., 2004), which also has implications for theories of human language processing (Coltheart et al., 2001; Levy, 2008; Norris, 2006; Reichle et al., 1998). These debates concentrate on known theoretical implications for the functional form and interaction structure of language processing effects, but, given the complexity of the task of inferring meaning from language, it is likely that there exist other kinds of nonlinearities and effect interactions not yet covered by existing theory. Discovering such patterns could advance the field, but this is not possible in standard analysis frameworks unless analysts deliberately look for them.

### Assumption: Stationarity (Time-Invariance)

Naively implemented, LM, GAM, GAMLSS, and CDR models of time series assume a stationary (time-invariant) function mapping predictors to responses. If the underlying response function is nonstationary (time-dependent), this can lead to poor fit and misleading estimates. Some control of nonstationarity is nonetheless possible under these approaches by including autoregressive terms (Baayen et al., 2017) or adding temporal features to the predictors (Baayen et al., 2018). The kinds of nonstationarity that models can capture is thus determined by the kinds of effects they can capture: LMs and CDR can capture nonstationarity in the form of linear trends along some representation of the time dimension, whereas GAMs and GAMLSS can also capture nonlinear effects of time via spline functions.^3^

Existing evidence indicates that responses in studies of human language processing are nonstationary, and in ways that arguably affect scientific inferences if not taken into account. For example, participants are known to habituate strongly to tasks in language processing experiments, such that e.g., response times decrease dramatically and nonlinearly over the course of the experiment (Baayen et al., 2018; Prasad & Linzen, 2021). Prasad and Linzen (2021) have even argued that this task adaptation effect may have driven previous reports of “syntactic priming” (Fine et al., 2013), and that syntactic priming effects may only be detectable with much larger sample sizes. Baayen et al. (2018) have likewise argued for an important influence of latent factors like attention and fatigue, which change over time, affect responses, and cannot be directly observed. The full extent of the impact of these kinds of nonstationarities on estimates of cognitive effects of interest is not yet well understood.

### Assumption: Homoscedasticity (Constant Variance)

LMs, GAMs, and CDR all assume a homoscedastic data-generating model: the predictors influence the mean response, but the variance (and/or any other distributional parameter) is treated as constant across time. The many ways in which this assumption can be violated by time series is the subject of a vast statistical literature (Cox & Isham, 1980; Engle, 1982; Koyck, 1954; Sims, 1980), as are the implications of such violations for statistical inferences (Cattaneo et al., 2018; Long & Ervin, 2000; Rosopa et al., 2013; Trenkler, 1984; You et al., 2007). These concerns take on special importance for (a) analyses in which the entire distribution over the response (not just the expectation) is a quantity of interest, or (b) likelihood-based out-of-sample comparisons between hypotheses, where poor fit between the modeled and true response distribution can lead to failure to generalize.

Both of these concerns are pertinent to the study of language processing. For example, prior work has argued that cognitive variables like word frequency and predictability have differential effects on different parameters of the distribution of eye gaze during reading, and thus correspond to distinct cognitive mechanisms (Staub, 2011; Staub et al., 2010). In addition, with growing interest in larger-scale naturalistic datasets for language processing research (Cop et al., 2017; Futrell et al., 2021; Kennedy et al., 2003; Luke & Christianson, 2016; Shain et al., 2020) comes the growing possibility of drawing conclusions from overfitted statistical models of these data. One approach to addressing this possibility is to perform statistical comparisons based on the likelihood assigned by models to out-of-sample data, ensuring that tests favor models with more generalizable descriptions of the modeled system (Shain et al., 2020; Shain & Schuler, 2021). This approach crucially relies not just on an accurate model of the expected response, but on an accurate model of the *distribution* of responses. Models that fail to capture the structure of that distribution will struggle in the out-of-sample evaluation, with poor likelihood at points where the variance is over- or underestimated.

## MATERIALS AND METHODS

In order to establish the validity and utility of our proposed approach, we analyze the properties of CDR-NN models fitted to a variety of datasets. We focus our discussion of results on data from human language processing experiments, but we also conduct extensive analyses on synthetic data as described in SI G. The purpose of these empirical analyses is to exemplify the inferential gains afforded by CDR-NNs over alternative methods, especially for observational, naturalistic data. Since our target contribution is primarily methodological, we do not intend these analyses or their interpretations to advance novel scientific claims or theories.

### Datasets

#### Eye-Tracking (Dundee).

Dundee (Kennedy et al., 2003) is an eye-tracking dataset containing newspaper editorials read by 10 participants. The dataset contains a total of 340,840 events (where one event is a single participant’s eyes entering and then exiting a single word region). Studies of language processing use measures derived from the eye-tracking record as indices of readers’ comprehension difficulty, in order to test theories about the underlying comprehension processes. A number of such measures exist in the literature (Rayner, 1998). In this work, we use the following three measures:

- *Scan path duration*: time elapsed between entering a word region and entering a different word region.
- *First pass duration*: time elapsed between entering a word region from the left and entering a different word region.
- *Go-past duration*: time elapsed between entering a word region from the left and entering a different word region to the right.

Following Shain and Schuler (2021), unfixated items were excluded as well as (a) items following saccades longer than 4 words, (b) starts and ends of sentences, screens, documents, and lines, and (c) items whose duration included a blink (Schotter et al., 2018).

#### Self-Paced Reading (Natural Stories).

Natural Stories (Futrell et al., 2021) is a crowd-sourced self-paced reading (SPR) dataset consisting of narratives and non-fiction passages read by 181 participants. In a self-paced reading task, participants step through words in the passage on a screen by pressing a button, and the time between button presses is recorded. The dataset contains a total of 1,013,290 events (where one event is a single participant viewing a single word token). Following Shain and Schuler (2021), items were excluded if they have fixations shorter than 100 ms or longer than 3000 ms, if they start or end a sentence, if the participant missed 4 or more subsequent comprehension questions, or if the participant had fewer than 100 responses after application of the other filters.

#### Functional Magnetic Resonance Imaging (Natural Stories).

The Natural Stories fMRI dataset (Shain et al., 2020) contains fMRI responses from 78 participants who listened to audio recordings of the Futrell et al. (2021) materials while in an MRI scanner. Following Shain and Schuler (2021), we modeled mean activity in the core language network: six left-hemisphere fronto-temporal functional regions of interest (fROIs) that were functionally identified in each individual participant, treating *fROI* as a random effect in addition to *Participant*.

#### Data Split.

Each dataset is partitioned into training (50%), exploratory (25%), and test (25%) sets, using the same partitioning scheme as Shain and Schuler (2021). For reading, the partition respects the non-independence of words within the same sentence, using modular arithmetic to cycle sentence IDs *e* across the partition with a different phase for each participant *u*: partition(*e*, *u*) = (*e* + *u*) mod 4, assigning outputs 0 and 1 to the training set, 2 to the exploratory set, and 3 to the test set. For fMRI, where the units of the response are images (TRs) acquired every 2 s, the partitioning strategy follows a similar approach, only cycling 30 s (15 TR) chunks of consecutive imaging data, rather than sentences (which cannot be cleanly separated in naturalistic fMRI): partition(*e*, *u*) = $\left\lfloor \frac{e+u}{15}\right\rfloor$ mod4, again assigning outputs 0 and 1 to the training set, 2 to the exploratory set, and 3 to the evaluation (test) set.

### Predictors

We use the same predictors as Shain and Schuler (2021), namely:

- **Rate** (ET, SPR, fMRI): a “deconvolutional intercept”; that is, a timestamped vector of 1’s that is convolved by the model to yield an IRF representing the baseline response to an event, so named because variability in the response is driven by the rate of stimulus events in time.
- **Unigram surprisal** (ET, SPR, fMRI): the negative log probability of a word derived from a KenLM unigram model (Heafield et al., 2013) trained on the Gigaword 3 corpus (Graff et al., 2007).
- **5-gram surprisal** (ET, SPR, fMRI): the negative log probability of a word in context derived from a KenLM 5-gram model trained on the Gigaword 3 corpus.
- **Word length** (ET, SPR): word length in characters.
- **Saccade length** (ET): incoming saccade length in words.
- **In regression** (ET): whether a fixation is part of a regressive (backward) eye movement.
- **Previous was fixated** (ET): boolean indicator for whether the preceding word was fixated.
- **Sound power** (fMRI): Root mean squared signal power of the audio recording as computed by the Librosa software library (McFee et al., 2015).

To account for the possibility of qualitatively different scan path responses to linguistic variables in regressive vs. non-regressive eye movements, in the Dundee scan path analyses we follow Shain and Schuler (2021) in partitioning all variables in the scan path analyses into +reg and −reg variants as a function of whether the fixation occurred within a regression (+reg) or not (−reg). Indexical predictors used by Shain and Schuler (2021), such as the position of the word within the experiment, are not needed in a CDR-NN framework due to nonstationarity, and are therefore omitted. For detailed motivation and interpretation of this set of language processing variables, see Shain and Schuler (2021).

### Model Estimation

The parameters **v**_**0**_ and **V** can be estimated using any supervised learning procedure. In our implementation, we implement the model as a computation graph in the TensorFlow library (Abadi et al., 2016) and estimate its parameters (including all layer weights and biases, as well as coefficients and biases for the response distribution, both fixed and random) using stochastic gradient descent (specifically, the Adam optimizer, Kingma & Ba, 2014).

### Model Design

We start from a “base” set of hyperparameters (see SI D) manually selected based on a combination of factors, including parsimony, training speed, validation set performance, and consistency of estimates/performance across replicates. To explore the influence of these hyperparameter choices, we perform a limited grid search over models that deviate (up or down) from the base configuration in one of the following dimensions: number of hidden layers in the IRF, number of units per hidden layer of the IRF, L2 penalty strength on the IRF weights, L2 penalty strength on random IRF effects by participant, dropout level, learning rate, and batch size.

### Model Convergence

Convergence diagnosis follows the time-loss criterion of Shain and Schuler (2021). In brief, the correlation of a performance metric with training time is tested statistically using *α* = 0.5 until at least half of the most recent 100 training epochs have failed to reject the null hypothesis of no correlation, indicating that performance has stopped increasing. For full details, see Shain and Schuler (2021). The fMRI models used to exemplify exploratory and confirmatory CDR-NN analysis used out-of-sample exploratory set likelihood (evaluated every 10 training epochs) as the diagnostic metric. All other models used in-sample training set likelihood (evaluated every epoch) as the diagnostic metric.

### Model Comparison

**Figure 2.**
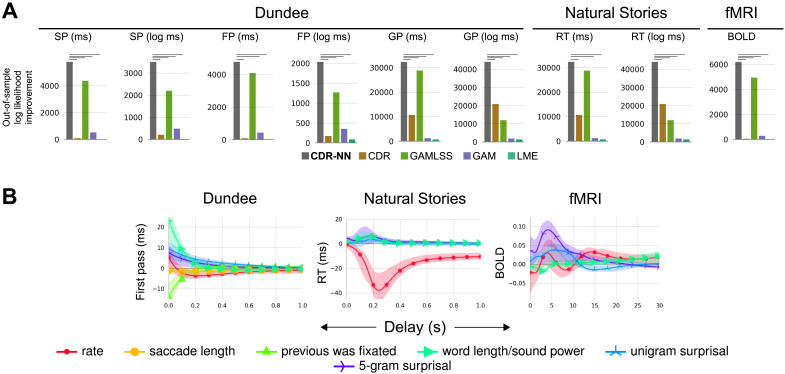
**Main result.** **A.** Out-of-sample (test set) log likelihood improvement of models over baseline using raw and log-transformed scan path (SP), first pass (FP), and go-past (GP) durations from the Dundee eye-tracking dataset, raw and log-transformed reading times (RT) from the Natural Stories self-paced reading dataset, and blood oxygen level dependent (BOLD) contrast from the Natural Stories functional magnetic resonance imaging (fMRI) dataset. See Methods for technical details. Significant improvements from CDR-NNs over alternatives are indicated by horizontal lines in each subplot. CDR-NNs generalize numerically better than all alternatives for all response variables, significantly so in all but two comarisons. **B.** CDR-NN-estimated impulse response functions. Curves represent the estimated change in response (*y*-axis) from one standard deviation increase in each predictor (line color) as a function of delay (in seconds) from word onset (*x*-axis).

Performance of CDR-NN models is statistically compared to that of LME (Bates et al., 2015), GAM (Wood, 2006), and GAMLSS (Rigby & Stasinopoulos, 2005) models. For Dundee and Natural Stories self-paced reading, we consider variants both with and without three additional lags per predictor to help capture delayed effects. For the LME/GAM Natural Stories fMRI baselines (but not for CDR or CDR-NN), we pre-convolve the predictors with the canonical HRF, following evidence from Shain and Schuler (2021) that this approach outperforms alternatives (linear interpolation, temporal binning, and Lanczos interpolation) that attempt to fit the HRF using discrete-time approaches. Lagged regressors are therefore not included in the fMRI models, since the delays are already taken into account by the assumed HRF. Performance gains in Figure 2A are relative to the least expressive model overall (LME with no lagged predictors). For the Dundee and Natural Stories SPR datasets, LME and GAM baseline results in Figure 2A reflect the performance of models with additional lagged predictors (which is why LME performance differs from baseline in these cases). For the fMRI dataset in which no lagged predictors were used, the LME performance gain is 0 because the LME model is identical to the baseline. CDR-NNs are also compared to (non-neural) CDR (Shain & Schuler, 2021).

Following Shain and Schuler (2021), LME and GAM models include by-participant random effects for every fixed effect in the model. We attempted to follow this protocol with GAMLSS but found this to result in a range of numerical problems and fatal crashes. GAMLSS models would only reliably run to completion when all random effects were removed except the by-participant intercept, which is the configuration used in all reported experiments. All GAMLSS nonlinearities assume penalized B-splines with default parameters.

All baseline models except the GAM and GAMLSS models for the fMRI dataset are the same as those used in Shain and Schuler (2021). All statistical comparisons use paired permutation tests (Demšar, 2006) of the conditional log likelihood assigned by each model to an out-of-sample test set. To avoid unnecessary statistical comparisons, only the reference implementation of CDR-NN (CDR-NN base, see SI D) is evaluated on the test set. For technical description of the permutation testing procedures, see SI J. For reader-friendly versions of the R-style model formulae used to define each baseline, see SI K. Full implementation details necessary for reproducing both the baseline and CDR-NN models are available in the public codebase: https://github.com/coryshain/cdr.

## RESULTS

### Model Validation

We first validate the model by establishing comparable or improved generalization performance to the alternative analysis approaches reviewed above (for confirmation that CDR-NNs successfully recover ground-truth models from synthetic data, see SI G). As stressed in Shain and Schuler (2021), the principal advantages of our proposal relative to existing alternatives are scientific, namely *insight* and *control*: CDR-NNs can be used to answer difficult questions while controlling for difficult potential confounds. Under these assumptions, generalization performance is of little intrinsic interest: unlike e.g., machine learning applications like image recognition, scientists are typically not interested in maximizing the predictive accuracy of a statistical model for its own sake. They are instead interested in how the statistical model can inform their *understanding* of the phenomenon they are studying. Our proposal stands to offer such insights thanks to its design principles. But can insights from a novel method be trusted? To address this question, it is helpful to establish a standard of reference against which the new approach can be compared. Here, we use established regression techniques to define that reference, and we show that the description of the data provided by our method is no worse (and indeed, substantially better) than that provided by standard tools.

The key finding of our validation study is shown in Figure 2A: CDR-NNs generalize substantially better to unseen data than comparable LME, GAM, GAMLSS, or CDR baselines, numerically improving conditional out-of-sample log likelihood in each comparison (often by thousands of points, significant in all but two comparisons). GAMLSS is the the best-performing alternative, suggesting that CDR-NNs’ gains in these analyses derive primarily from capturing heteroscedasticity, which standard implementations of the other models cannot do (see SI H for additional support for this conjecture). Nonetheless, CDR-NNs also yield consistent gains over GAMLSS, suggesting that CDR-NNs’ advantages go beyond heteroscedasticity. Full results and analysis, including detailed exploration of diverse hyperparameter choices, are reported in SI G (synthetic datasets), SI H (human behavioral and neuroimaging datasets), and SI I (consistency of performance across model replicates).

Not only do CDR-NNs provide a generalizable description of complex processes, but their estimates are also richly detailed. Figure 2B shows IRF plots describing the estimated change in the response associated with one standard deviation increase in a predictor, as a function of delay from word onset (*x*-axis). As shown, most effects in reading (left and center) decay to near-zero within a one second window of stimulus presentation, whereas the fMRI response (right) is more diffuse, extending over 20–30 s and showing the characteristic hemodynamic peak around 5 s delay (Boynton et al., 1996). As has been previously reported, self-paced reading (center) is dominated by a large negative effect of *rate* (the average effect of reading a word), suggesting that fast reading in the recent past engenders fast reading now, consistent with an inertia effect from repeated button pressing (Shain & Schuler, 2021). All three modalities show a large positive effect of *5-gram surprisal*, a measure of how predictable a word is from context. This indicates an increase in both reading time and brain activity for less predictable words, consistent with predictive coding (Levy, 2008; Wilcox et al., 2020; Shain et al., 2020).

### Pattern Discovery and Hypothesis Testing

**Figure 3.**
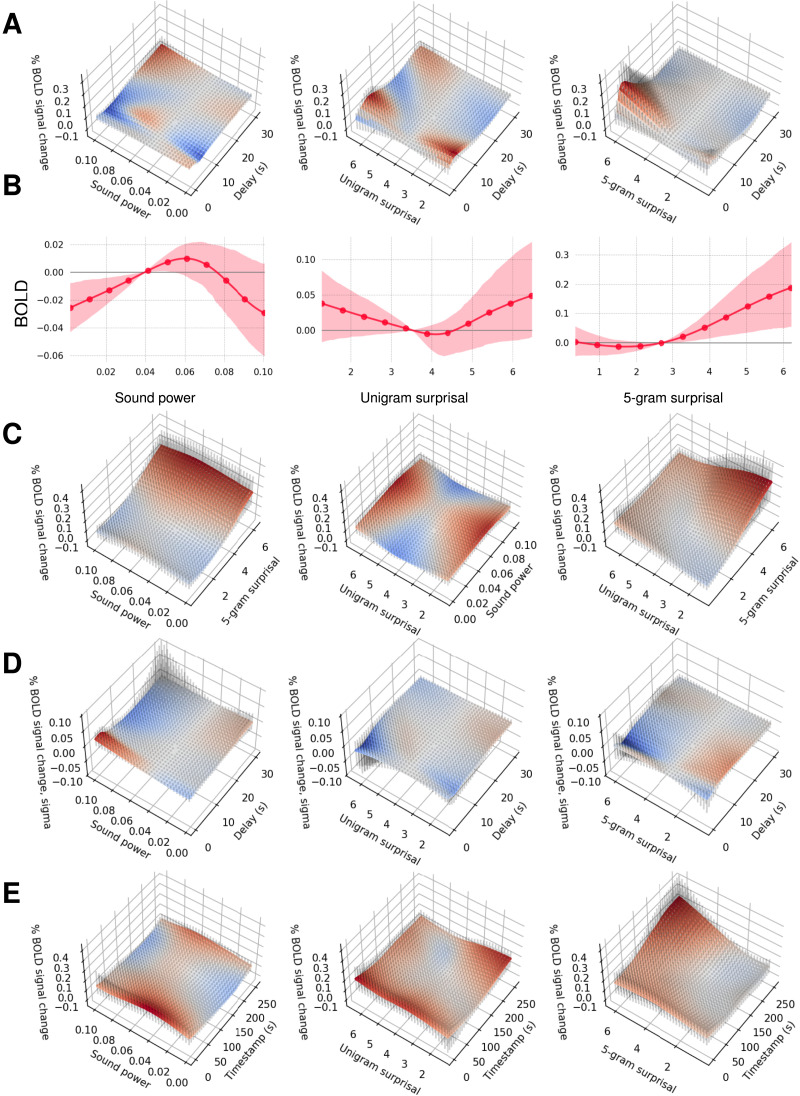
**CDR-NN estimates derived from the Natural Stories fMRI dataset.** Colored bands (line plots) and vertical error bars (surface plots) show Monte Carlo estimated 95% credible intervals. **A.** Univariate IRFs (hemodynamic responses). **B.** Functional form of effects at 5 s delay. **C.** Effect interactions at 5 s delay. **D.** Univariate IRFs of the *σ* parameter of the predictive distribution. **E.** Nonstationarity at 5 s delay.

We now exemplify applications of CDR-NNs for both exploratory research (e.g., discovery and visualization of novel patterns) and scientific hypothesis testing. In so doing, we show that, in addition to improving model fit and reducing dependence on standard simplifying assumptions, CDR-NNs broaden the space of questions that are feasible to investigate using a given dataset. We focus for simplicity on the Natural Stories fMRI dataset, since latencies are known to have a major influence on the fMRI BOLD signal (Boynton et al., 1996). To do so, we fit an ensemble of 10 CDR-NN models using the base configuration for fMRI (see SI D). From this single ensemble, we can obtain diverse estimates about the structure of the fMRI response (Figure 3), which we discuss below in five examples. We test these estimates statistically using out-of-sample model comparison, thereby grounding results in the generalizability of findings (for details, see SI J). We stress that the purpose of these analyses is to exemplify possible inferential gains afforded by our proposed method, rather than to advance scientific theories. Our discussion of these results is therefore cursory and restricted to either (i) sanity checks against bodies of existing evidence for prior claims or (ii) speculative interpretation of exploratory analyses.

#### Example A: The Existence of Effects.

Figure 3A shows the estimated change in blood oxygen level dependent (BOLD) contrast as a surface relating predictor value and delay from stimulus onset (the line plots in Figure 2B are obtained by “slicing” these surfaces along the *Delay* dimension at a fixed predictor value, i.e., one standard deviation above the training set mean). These plots reveal how the response is expected to change at a given delay after observing a predictor of a given value, holding all other variables constant. As shown, the language-selective regions whose activity is represented in this dataset are not very responsive to *sound power* (n.s.), a low-level auditory feature: the uncertainty interval includes zero over nearly the entire surface. There is an estimated effect of *unigram surprisal* (*p* < 0.0001)—a measure of how frequently a word is used—that better matches the expected hemodynamic shape (peaking around 5 s delay and then dipping), with an intriguing *u*-shaped nonlinearity such that both low and high unigram surprisal (that is, respectively, highly frequent and highly infrequent) words yield an increase in BOLD relative words with average frequency. The largest effects are associated with *5-gram surprisal* (*p* = 0.017).

#### Example B: Linearity of Effects.

The surfaces in Figure 3A visually suggest nonlinear effects of some predictors. These can be visualized more clearly by “slicing” along the predictor dimension at a fixed delay, as shown in Figure 3B (for simplicity, all plots at a fixed delay use 5 s, the approximate location of the peak response). Results of out-of-sample tests comparing models that enforce linear effects to models that allow nonlinear effects support the existence of nonlinearities, especially a nonlinear effect of *unigram surprisal* (*p* < 0.0001)—which is estimated to have a *u*-shaped effect (Figure 3B), and a nonlinear effect of *5-gram surprisal* (*p* < 0.0001)—which is estimated to have an inflection point, rising more steeply at higher values (Figure 3B).

#### Example C: Effect Interactions.

CDR-NNs implicitly model interactions between all predictors in the model. The three pairwise effect interactions at 5 s delay are plotted as surfaces in Figure 3C. As shown, CDR-NNs can discover both invariance and dependency between predictors. The effects of *unigram surprisal* and *5-gram surprisal* are largely invariant to *sound power*: the same basic response pattern holds regardless of the value of *sound power*. By contrast, *unigram surprisal* and *5-gram surprisal* appear to interact: the unigram effect flips direction (from increasing to decreasing) as one ascends the 5-gram continuum, and the 5-gram effect is more pronounced at lower values of unigram surprisal. This interaction is nonlinear, since it could not be well approximated by a coefficient on the product of the two predictors. However, this interaction does not improve test set likelihood and is therefore not significant. This outcome demonstrates that increased representational capacity (i.e., ability to model an interaction) does not automatically lead to improved generalization performance. This is an essential safeguard built into our proposed approach to hypothesis testing, which only admits effects that generalize robustly.

#### Example D: Distributional Regression.

Because CDR-NNs can quantify the effect of a predictor on all parameters of the response distribution, not just the mean, they can be used to address questions about the distribution of a given response measure. For example, word frequency and predictability have been argued to affect the location parameter but not the variance parameter of the distribution of fixation durations during reading (Staub et al., 2010; Staub, 2011). The IRFs relating each predictor to *σ* (the square root of the predictive variance) are plotted in Figure 3D. As shown, *sound power* has little effect on *σ*, and both *unigram surprisal* and *5-gram surprisal* are associated with a drop in *σ* near the peak hemodynamic response of about 5 s delay. Of these, only the *5-gram surprisal* effect is significant (*p* < 0.0001).

#### Example E: Nonstationarity.

By conditioning the IRF on a representation of elapsed time, CDR-NNs can capture nonstationarity in the response function. For example, the effect of word predictability may change nonlinearly over the course of story listening. Such nonstationarities are central to critical questions about adaptation and learning during language processing (Fine et al., 2013; Prasad & Linzen, 2021). Nonstationarity plots for the fMRI dataset are given in Figure 3E. These plots represent the effect of a predictor at 5 s delay as a function of its onset timestamp. As shown, *unigram surprisal* effects appear weaker toward the end of the story (but not significantly so, *p* = 0.060), whereas *5-gram surprisal* effects are stronger toward the end of the story (*p* = 0.0003).

#### Assumptions Influence Test Results.

Avoiding assumptions of linearity, stationary, and homoscedasticity can be critical for hypothesis testing, even if the research hypothesis does not directly concern these assumptions. For example, in Example C, we did not find a significant interaction between *unigram surprisal* and *5-gram surprisal*. However, when we enforce a homoscedasticity assumption, the interaction becomes significant (*p* < 0.0001). Likewise, in Example D, we did not find a significant effect of *unigram surprisal* on the *σ* parameter of the response distribution. However, when we enforce a linearity assumption, then the effect of *unigram surprisal* on *σ* becomes significant (*p* < 0.0001). These significant findings turn out to depend critically on implausible simplifying assumptions that CDR-NNs can relax. Precisely how a given simplifying assumption could affect a given experimental outcome is often difficult to anticipate. CDR-NNs help mitigate such concerns by avoiding these assumptions in the first place.

## DISCUSSION

We have proposed continuous-time deconvolutional regressive neural networks (CDR-NNs), a new approach to analyzing the kinds of observational time series data that are increasingly used to study the mind and brain. CDR-NNs leverage the flexibility of deep learning to relax standard assumptions in regression analyses of time series (discrete time, linearity, stationarity, and homoscedasticity) while remaining interpretable. This property enables flexible visualization and discovery of novel patterns in exploratory analyses and better control of confounds in confirmatory analyses.

### Model Quality, Flexibility, and Generality

We evaluated CDR-NNs on data from the domain of human language processing and showed that they substantially improve fit to unseen data over established alternatives (Figure 2A) while providing detailed and plausible estimates of the dynamics of the modeled system (Figure 2B). We then exemplified how a CDR-NN can be used to visualize and test diverse properties of the response, including the existence of effects (Figure 3A), the functional form of effects (Figure 3B), the possibility of arbitrary nonlinear effect interactions (Figure 3C), effects on the probability distribution over the response (Figure 3D), and changes in effects over time (Figure 3E). In fact, CDR-NNs can be used to visualize *any* property of the modeled system that can be cached out as a question about the response to input. Likewise, CDR-NNs can be used to test any null hypothesis that can be cached out as a model constraint (see SI J for discussion). CDR-NNs therefore constitute a highly general framework for estimating and testing the properties of continuous-time processes in nature.

Thanks to this generality, CDR-NNs are also appropriate for modeling time series in which the response is measured with high temporal resolution relative to the stimulus events. Examples of such domains include electroencephalography (EEG), magnetoencephalography (MEG), electrocorticography (ECoG), and single-unit spike recordings, which are widely applied to study brain responses in both human and non-human animals. For example, “event-related potentials” (ERPs, e.g., the N400) in electroencephalography reflect the estimated change in voltage at a particular location on the scalp following stimulus presentation as a function of delay; ERPs are are thus a special case of IRF and can be estimated using the CDR-NN methods we have proposed. We have chosen not to focus on such domains in this study because the high frequency of the brain signals (often hundreds of times the frequency of stimulus events like words or images) renders them more amenable to discrete-time analysis (FIR, epoching, etc; see e.g., Smith & Kutas, 2015) than the lower-resolution signals we have targeted here (behavioral and fMRI measures). Nonetheless, CDR-NNs stand in principle to offer advantages even in high-temporal-resolution domains. *First*, CDR-NNs permit improved control over nonlinearity, nonstationarity, and heteroscedasticity, as stressed above. *Second*, CDR-NNs enable deconvolution from overlapping responses in observational or naturalistic data, which are increasingly of interest. *Third*, as neural networks, CDR-NNs might be useful for nonlinear feature learning from multivariate input representations like pixel intensities or acoustic power spectra, in which the dimensions lack a semantic interpretation. For example, *f*_in_ could be defined so as to map pixel-level stimulus data nonlinearly into a small number of latent impulse dimensions, which are then convolved by the IRF to generate a response distribution. These latent dimensions would constitute a compression code representing the features of the stimulus time series that are most strongly related to brain activity, which could then be interpreted using black box interpretation techniques like perturbation analysis (Petsiuk et al., 2018; Ribeiro et al., 2016). We therefore see a wide range of candidate applications for our method in cognitive and brain sciences beyond those that we have directly explored here, which we leave to future work.

### Exploratory Insights Into Human Language Processing

The findings from these empirical evaluations also offer key insights about the brain response to language, some consistent with prior expectations, and some novel. First, consistent with prior expectations (Fedorenko et al., 2010), the high-level language network in the brain is sensitive to linguistic variables (*unigram surprisal*, a measure of word frequency, and *5-gram surprisal*, a measure of word predictability) but not a perceptual variable (*sound power*). This outcome also entails that effects of word frequency and predictability are at least partly dissociable in brain activity (since both effects together significantly improve on either effect individually), consistent with prior arguments for such a dissociation in reading (Staub, 2015).

Second, word frequency and predictability both have nonlinear effects on brain activity. The predictability effect is superlinear, with an inflection point near the mean below which effects are weak, and above which effects are strong. Although this is inconsistent with prior claims that processing cost is linear on our predictability measure (Smith & Levy, 2013; Wilcox et al., 2020), it should be taken with a grain of salt: BOLD is a complex signal that is not necessarily linear on neuronal activity (Logothetis, 2008), so it may be a problematic testbed for questions about functional form. To our knowledge, the finding of a *u*-shaped frequency effect (whereby BOLD increases when word frequency is *either* low or high) is novel and warrants further investigation: although BOLD may not be linear on neuronal activity, evidence indicates that it is monotonic (Logothetis et al., 2001), and thus our findings plausibly reflect a *u*-shaped effect at the neuronal level.

Third, results do not support a frequency-predictability interaction: although a nonlinear interaction appears in the estimates (Figure 3C), it does not generalize. Several prior studies have also failed to find such interactions, leading some to argue that frequency and predictability effects are driven by distinct cognitive mechanisms (for review, see Staub, 2015).

Fourth, results support an influence of word predictability on the scale parameter (*σ*) of the distribution over brain activity. This finding is novel: in a prior reading study, predictability did not affect *σ* (Staub, 2011). Our results do not contradict this earlier work, since the fMRI BOLD response is a different measure than fixation durations during reading, with different distributional properties. We speculate that predictability effects on the scale parameter may derive from less predictable words driving the BOLD response above the noise floor, thereby increasing model certainty about the expected BOLD value and thus decreasing *σ*.

Fifth, we find significant non-stationarity in the predictability response, such that less predictable words are associated with larger increases in BOLD as the story unfolds. To our knowledge, this finding is novel. It is possible that comprehenders increase their reliance on predictive processing later in the story, as they accumulate evidence toward a mental model of story content that might facilitate accurate prediction. We leave detailed follow up of all of the above findings to future work.

### Limitations

Like all modeling approaches, CDR-NNs have potential drawbacks. *First*, they often require more data and computation. However, in practice, given the complexity of the problem they are tasked to solve (arbitrary nonlinear and nonstationary continuous-time influences of all possible sets of predictors on all parameters of the response distribution), they can be quite efficient. For example, the Dundee model used in this study contained only 3484 trainable parameters—a tiny network by modern deep learning standards—and trained in a few hours. *Second*, CDR-NNs (like all deep neural networks) are vulnerable to local optima. However, in an out-of-sample testing paradigm, training set likelihood maximization is not the goal, but rather *generalization*. To this end, CDR-NNs can leverage the many existing techniques for robust generalization in deep neural networks (Srivastava et al., 2014), and variability in performance can be mitigated through ensembling (see SI J). Even in cases where lack of data prohibits out-of-sample evaluation, CDR-NNs can be used to complement results from existing approaches, since they can visualize how estimates differ when simplifying assumptions are relaxed. *Third*, due to their implementation as deep neural networks, precise mathematical analysis of CDR-NN models is generally intractable. This issue can usually be overcome by Monte Carlo simulations. However, the computational intensity of these simulations can vary considerably according to the question being asked. For example, approximate credible intervals for IRF estimates can be obtained cheaply from a single fitted model or model ensemble (SI F). Likewise, questions about model identifiability given data can be addressed relatively cheaply by fitting models to a simulated dataset in order to study how well ground-truth features of interest are recovered. This approach is exemplified by our synthetic analyses reported in SI G. By contrast, unlike e.g., simple linear models (whose power properties depend only on sample size and effect size), the power properties of CDR-NN models implicitly depend on a constellation of other factors. In particular, CDR-NNs do not merely estimate a vector of effect sizes, but a manifold over the vector space defined by the predictors. Power thus depends not only on effect sizes, but also on effect shapes (in predictor space and over time) and on the sizes and shapes of interactive relationships between predictors. In addition, due to the use of held-out testing for model comparison, the effect of sample size on power must be subdivided into (i) the fidelity of model identification from a given amount of training data and (ii) the likelihood of confirming a model on a given amount of testing data. Thus, questions of power can be answered in simulation but only at significant computational expense, since the simulations require fitting many models to many synthetic datasets. Developing and evaluating procedures to automate simulation-based power analyses for CDR-NNs is a target of future work. However, we stress that our primary target use case is large-scale observational data for which power considerations may be less of a concern. *Fourth*, like all deep neural networks, there are many choice points in CDR-NN model design, including the number of layers and hidden units, squashing functions, regularization and dropout strength, learning rate, and batch size. Choices along any of these dimensions can materially impact effect estimates and generalization performance. However, supplemental analyses show that model estimates are quite stable across diverse hyperparameters (SI H). Furthermore, model comparisons are based on *relative* performance between more and less constrained models within a given hyperparameterization, so it is not necessary to maximize absolute generalization performance in order to make comparisons. Our software implementation of CDR-NNs (https://github.com/coryshain/cdr) distributes with detailed documentation, provides default hyperparameters that are reasonable for many cases, and requires no familiarity with programming or deep learning. Finally, in case of discrepant results between two hyperparameterizations, there is a simple model selection principle: prediction likelihood. That is, the results from the hyperparameterization with the higher out-of-sample likelihood should be trusted more. This principle also permits the use of model selection based on validation set performance in order to adapt CDR-NNs to new domains, although such tuning may not be necessary in many cases, since the default parameters used in this study generalize well across diverse datasets (SI H). Note that out-of-sample model selection obviates the need for the heuristic penalties on model complexity assumed in commonly-used information criteria (Akaike, 1974; Schwarz, 1978), since it directly quantifies the prediction likelihood that information criteria implicitly estimate.

### Notes for Practitioners

Since we are not only proposing a mathematical model but also offering an implementation for practical use, here we highlight some important general considerations for future users of our software library (detailed user documentation is available online: https://github.com/coryshain/cdr). This list is not intended to exhaust the space of possible modeling issues, but instead to point out some of the key considerations that may not be immediately obvious to users of standard regression analyses.

*First*, as noted above, a major practical consequence of using neural networks is the large number of experimenter decisions (hyperparameters) that they introduce, relative to more familiar approaches like linear mixed models. Our own analyses already focus on what we consider to be the most important of these hyperparameters, namely depth, layer width, regularizer strength, dropout level, learning, rate, and batch size (see SI D), but these and other hyperparameters can be configured by the user, as described exhaustively in the documentation. We have attempted to provide reasonable default settings for the domains we have modeled in this study, but this does not guarantee hyperparameter optimality for arbitrary datasets.

*Second*, and by consequence, CDR-NN analyses may benefit from hyperparameter *tuning* (see e.g., Yang & Shami, 2020, for review), whereby different hyperparameter configurations are compared according to their fit to unseen data. If models converge with high uncertainty or poor generalization performance, suboptimal hyperparameterization could be to blame, and tuning should be considered. That said, we do not mean to imply here that it is always necessary to tune CDR-NN models or compare them to non-CDR-NN baselines, and we expect our default settings to generalize well on average given the diversity of real and synthetic data on which we have tried them here. As stressed above, the key criterion of interest for CDR-NN-based analyses will usually be the *relative* fit of models with different specifications, which can be assessed even if hyperparameters are suboptimal.

*Third*, as a consequence of the two preceding points, it is strongly recommended to partition datasets into training, validation, and test sets (as we have done here) prior to any model fitting. The use of a test set permits generalization-based tests, which are essential in this framework as previously discussed, and the distinction between the validation and test sets permits tuning without creating a multiple comparisons problem.

*Fourth*, as is common in neural network applications, it is a good idea to check models’ *learning curves* in order to diagnose common degeneracies like overfitting (which will be reflected as a simultaneous increase in training likelihood and decrease in validation likelihood). Overfitted models generalize poorly and thus may offer little reliable insight into the process under study. Overfitting can often be addressed with hyperparameters (e.g., increasing regularizer or dropout strength), but it can also be a sign that the model is not identifiable from a given dataset, and a simpler model or more data may be needed. Learning curve visualization is supported by our software implementation and can assist users in diagnosing these issues.

*Fifth*, modelers must assume the existence of nonlinear effects and arbitrary interactions between predictors unless models are expressly constrained to remove them. Examples of how to impose such constraints for testing purposes are provided in SI J.2.

### Conclusion

In conclusion, we have demonstrated that CDR-NNs obtain better estimates than standard regression analyses of observational time series and can directly capture complex nonlinear relationships between variables, permitting testing of fine-grained questions that are otherwise difficult to study. CDR-NNs therefore constitute an important advance for flexible and interpretable modeling of data that represent complex mental processes.

## ACKNOWLEDGMENTS

We would also like to thank Clara Meister and Tiago Pimentel for valuable discussion around bootstrap methods for hypothesis testing, and Ev Fedorenko for comments on the manuscript draft.

## FUNDING INFORMATION

C. S. was supported by a postdoctoral fellowship from the Simons Center for the Social Brain at MIT (via the Simons Foundation). W. S. was supported by the National Science Foundation grant #1816891. All views expressed are those of the authors and do not necessarily reflect the views of the National Science Foundation.

## DATA AVAILABILITY STATEMENT

All datasets analyzed in this study are publicly available and accessible online as described in Materials and Methods: Datasets. Code used to preprocess text and experiment data is available at https://github.com/modelblocks/modelblocks-release. Code to reproduce all analyses reported in this study is available at https://github.com/coryshain/cdr.

## Notes

^1^ Throughout this work, we refer to the dependent/endogenous variable as the *response* and its distribution as the *response distribution*.^2^ Note that because CDR subsumes linear mixed-effects (LME) models (since any LME model can be expressed as a CDR model where *g* of Equation 6 is fixed to be the Dirac *δ* function), discrete-time IRFs are still available when needed in a CDR framework, simply by including lagged regressors in the same way.^3^ Note that models thus defined are nonstationary only in that temporal features have been included in their inputs. The mathematical function mapping inputs to outputs remains stationary; that function can simply condition on a representation of time.

## Supplementary Material


