# Supplementary material for: A Deep Learning Approach to Analyzing Continuous-Time Cognitive Processes
Source: Open Mind (Camb). 2024 Mar 13;8:235–64. doi: 10.1162/opmi_a_00126 (PMC10962694; doi:10.1162/opmi_a_00126)
Supplement: Supplementary file 1 [file opmi-08-235-s001.pdf]

Supplementary Information for *A Deep Learning Approach to Analyzing Continuous-Time Cognitive Processes*

A Relationship to Shain (2021)

The central idea of this work—using a deep neural IRF kernel for deconvolutional regression—was proposed in a previous conference paper (Shain, 2021). However, the current study goes substantially beyond the previous one in several ways. First, unlike this work, Shain (2021) did not distinguish between the inputs and outputs to the deep neural IRF: every predictor was used as an input to the IRF and convolved using an output of the IRF. This distinction critically underlies our current CDR-NN definition's support for flexible hypothesis testing, since the IRF can now condition on predictors that it does not convolve or convolve predictors that it does not condition on. For example, to test linearity of effect A, a null model can now be built in which the IRF convolves but does not condition on A, thus enforcing linearity. The lack of this ability in Shain (2021) limited the scope of testable hypotheses relative to the current definition.

Second, we have improved the model design by factoring input processing from IRF computation. This permits dissociation of the IRF shape (over time) from the functional form of effects, allowing researchers to target these components of the response separately if desired. Such a factorization was not possible under the Shain (2021) design.

Third, we have broadened the scope of mixed effects modeling to encompass all trainable model parameters. By contrast, in Shain (2021), mixed effects were arbitrarily restricted to specific model components.

Fourth, we have expanded the feature set of the software implementation. For example, it is now possible to use a composite IRF consisting of multiple distinct deep neural transforms, each with their own parameterization. This feature is critical for testing hypotheses about whether a particular effect is e.g., stationary (by using two neural IRFs, a stationary one that convolves the critical predictor and a nonstationary one that convolves the other predictors) or specific to a certain distributional parameter (by using different neural IRFs for each distributional parameter). See **SI J** for additional examples.

Fifth, our mathematical definition (**Introduction: The CDR-NN Model** of the main article) has been simplified and clarified. It also now generalizes a range of related regression methods for time series, including linear models, generalized additive models, and kernel-based continuous-time deconvolutional regression models.

Finally, our empirical evaluation goes well beyond the cursory evaluation in Shain (2021), which only evaluated a handful of model designs, used a small subset of the full synthetic dataset, and did no hypothesis testing. By contrast, our study provides extensive evaluations on every dataset covered by Shain and Schuler (2021), permitting detailed comparison of the effects of different hyperparameterizations. In addition, we propose methods for hypothesis testing and demonstrate these methods for a diverse set of questions.

B Detailed Motivation for Proposed CDR-NN Architecture

Our proposed CDR-NN architecture (**Figure 1** of the main article) contains design elements whose motivation may not be immediately transparent to all readers. Therefore, in this section, we build up to our proposed architecture in a step-by-step conceptual progression, starting from a maximally simple multiple regression model. This progression is visualized in **Supplementary Figure S1**.

Supplementary Figure S1a depicts a simple multiple regression model consisting of a mapping from the predictor vector to the evoked effects on the expected response, relative to an intercept or bias term. If this mapping is linear, then **Supplementary Figure S1a** depicts a linear regression model. However, the mapping could be nonlinear, as in a generalized additive model or a neural network. Since (for reasons discussed in the

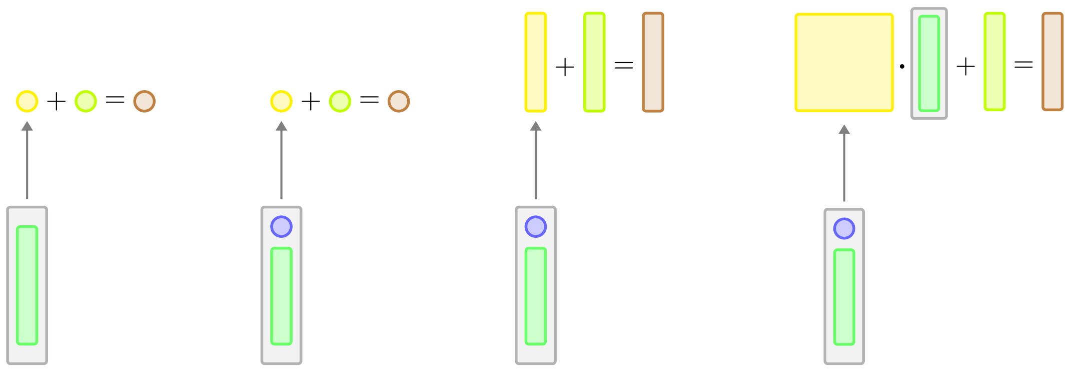

(a) A stationary, homoscedastic, non-deconvolutional nonlinear regression model

(b) A **nonstationary**, homoscedastic, non-deconvolutional nonlinear regression model

(c) A **nonstationary**, **heteroscedastic**, non-deconvolutional nonlinear regression model

(d) A **nonstationary**, **heteroscedastic**, non-deconvolutional nonlinear regression model that generates a *linear map* (yellow) from inputs to outputs, rather than outputs directly.

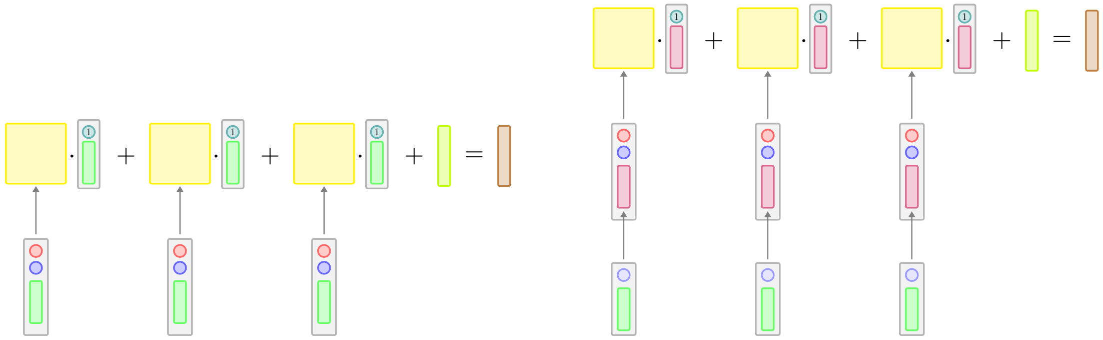

(e) A **nonstationary**, **heteroscedastic**, **deconvolutional** nonlinear regression model.

(f) A **nonstationary**, **heteroscedastic**, **deconvolutional** nonlinear regression model, that distinguishes *input processing* (bottom) from *impulse response modeling* (middle).

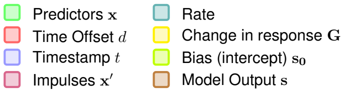

Figure S1: Step-by-step walk-through of the conceptual progression (top-left to bottom-right) from a simple (non-)linear regression modeling to our proposed CDR-NN architecture (Figure 1 of the main article). Scalars are shown as circles, vectors are shown as narrow boxes, matrices are shown as wider boxes, and deep neural network transformations are shown as arrows. A stationary, homoscedastic, non-deconvolutional regression model **(a)** generates the expected scalar change in a univariate response (relative to the intercept) through a (possibly nonlinear) transform of the predictor vector. This model cannot capture nonstationarity or heteroscedasticity, and it does not estimate a temporal IRF. The stationarity assumption can be relaxed as in **(b)** by allowing the model to condition on the timestamp of the input vector. The homoscedasticity assumption can be relaxed as in **(c)** by generating a vector of *parameters for the predictive distribution* over the response, rather than generating the expected response directly. However, this design makes it impossible to enforce linearity assumptions for the purposes of hypothesis testing, since the entire predictor vector is processed by a neural network. To address this, we instead use the neural network to generate a *linear map* from the predictor vector to the estimated change in each distributional parameter **(d)**. This design affords greater control by e.g., permitting ablation of predictors from the inputs to the neural network (only) while retaining them in the output, thereby enabling estimation of models that are strictly linear on one or more predictors. To equip models with the capacity to *deconvolve* by estimating a continuous-time impulse response, we (i) add the time offset t to the model inputs and (ii) add a bias term (*Rate*, a vector of 1's) that is convolved by the model **(e)**, allowing the model to estimate a multivariate IRF that captures the overall response to an input (*Rate*) as well as deflections from that overall response evoked by particular values of predictors, as a function of continuous time. However, this design makes it impossible to enforce a nonlinear Dirac δ IRF for the purposes of hypothesis testing. To address this, we distinguish between predictors $\mathbf{x}$ and impulses $\mathbf{x}'$ by allowing an additional neural network model f_{in} to generate $\mathbf{x}'$ from $\mathbf{x}$ **(f)**. This enables GAM-like nonlinear Dirac δ IRF estimation by e.g., retaining a predictor in the input to f_{in} but enforcing f_{IRF} to be Dirac δ.

main article) we seek to develop a neural network approach to time series regression, we allow this mapping to be instantiated as a neural network, and therefore to be potentially nonlinear on the predictors.

However, even assuming a neural network mapping, the model in **Supplementary Figure S1a** is highly constrained. In particular, it assumes stationarity over time, homoscedasticity, and an absence of delayed effects. To relax the stationarity assumption, we add the timestamp of the predictor vector to the inputs to the network, as shown in **Supplementary Figure S1b**. This allows the network to capture arbitrary nonlinear effects of time on the response evoked by the predictor vector. To relax the homoscedasticity assumption, we allow the model to generate a vector of parameters for the distribution over the response, rather than generating the expected response directly, as shown in **Supplementary Figure S1c**. In this way, uncertainty over the response is also modeled as a function of the predictors, thereby allowing for non-constant variance across the time series.

However, a major shortcoming of this more flexible model is that its flexibility cannot be constrained in any way. Predictors can be added to or removed from the model, but it would not be possible to e.g., force the model to respect a linear effect of a predictor. This severely restricts the range of hypotheses that can be tested (by restricting the range of constrained null hypotheses that can be implemented and compared to the alternative hypothesis of e.g., a nonlinear effect). To address this concern, we instead use the neural network to generate a *linear map* from predictor space to the parameter space of the response distribution (**Supplementary Figure S1d**). This permits greater control over the model by e.g., allowing a predictor to be removed from the input to the neural network while it is retained in the output, thereby enforcing a linear effect.

This model still lacks any capacity to estimate an IRF describing the change in evoked response over time. To address this, we additionally include the time offset d as an input to the network, and we add to the output of the linear map a bias term (*Rate*, a vector of 1's) capturing overall effects of stimulus timing (**Supplementary Figure S1e**). These modifications allow us to apply the network simultaneously to multiple inputs within some time window of the target response, resulting in a deconvolutional model that implicitly estimates an IRF describing the influence of an impulse as a function of (i) its properties and (ii) its distance in time from the target.

However, a major shortcoming of this design is that nonlinearity in predictor space cannot be estimated independently of the impulse response over time. This prevents independent statistical testing of these two aspects of the response. To allow the model to decouple nonlinearity in predictor space from the temporal dynamics of the response, we include an additional neural network component f_{in} for "input processing" (**Supplementary Figure S1f**). Nonlinearities in predictor space can be estimated by f_{in} while e.g., enforcing a Dirac δ IRF, allowing the model to approximate a GAM-like nonlinear regression model that lacks continuous-time temporal dynamics.

Note that the full model in **Supplementary Figure S1f** subsumes all submodels described in **Supplementary Figure S1**, and thus can be pared down to any of these submodels as motivated by the analyst's goals and domain knowledge. By manipulating information flow through this final network as exemplified above, a wide range of constrained null models can be implemented and statistically tested against their relaxations. Our proposed CDR-NN architecture thus provides a powerful and flexible framework for scientific inference.

C Algorithmic-Level Model Description

We have attempted to optimize our mathematical presentation (see **The CDR-NN Model** of the main article) for clarity, simplicity, and generality. As a result, our equations make few implementational commitments and are computationally inefficient if implemented naively (e.g., convolving over an entire dataset to generate a single response, or computing random effects via sparse matrix multiplication rather than indexing). This may pose difficulties for readers who seek a deeper understanding of our implementation, or who wish to build their own. Thus, for convenience, in this section we provide pseudocode presenting a high-level algorithmic description of a single minibatched training epoch (i.e., a single pass through the training data) in a CDR-NN model (**Algorithm 1**) **Algorithm 1** is not a formal definition (for that, see **The CDR-NN Model** of

Algorithm 1 Simplified example algorithm for a single training epoch in a batched CDR-NN implementation. We denote indexes or slices into arrays using subscript square brackets, with commas delimiting axes of the array. A slice between indices a and b (inclusive) is denoted with a colon $a : b$. A slice along an entire axis is denoted with $*$.

Require: $\mathbf{X} \in \mathbb{R}^{N \times K}$: predictors
Require: $\mathbf{t} \in \mathbb{R}^{N}$: predictor timestamps
Require: $\mathbf{Y} \in \mathbb{R}^{M \times Y}$: responses (matrix of M individual responses $\mathbf{y}$)
Require: $\tau_{1,\ldots,M}$: response timestamp for each of M responses
Require: $\mathbf{v_0} \in \mathbb{R}^{V}$: fixed effects parameter vector
Require: $\mathbf{V} \in \mathbb{R}^{V \times Z}$: random effects parameter matrix
Require: $\mathcal{Z}_{1,\ldots,M}$: set of random effects levels for each of M responses
Require: f_{in}: input processing function (feedforward neural network)
Require: f_{IRF}: impulse response function (feedforward neural network)
Require: $\mathcal{F}$: distributional family for the response
Require: H: number of preceding inputs to consider for each response
Require: B: minibatch size

$i \leftarrow 0$
while $i < M$ **do**
    ▷ *Data processing*
    $\mathbf{D}^{\text{batch}} \leftarrow \mathbf{0}^{B \times H}$ ▷ Batched time offsets between responses and predictors
    $\mathbf{X}^{\text{batch}} \leftarrow \mathbf{0}^{B \times H \times K}$ ▷ Batched tensor of predictors
    $\mathbf{T}^{\text{batch}} \leftarrow \mathbf{0}^{B \times H}$ ▷ Batched matrix of predictor timestamps
    $\mathbf{V}^{\text{batch}} \leftarrow \mathbf{0}^{B \times V}$ ▷ Batched matrix of random deviations in the parameters
    for $j = 1, \ldots, B$ **do**
        $k_e \leftarrow \max \left\{ 1 \leq k \leq N : \mathbf{t}_{[k]} \leq \tau_{i+j} \right\}$ ▷ Index of end of predictor window for response $i + j$
        $k_s \leftarrow k_e - H + 1$ ▷ Index of start of predictor window for response $i + j$
        $\mathbf{D}^{\text{batch}}_{[j,*]} \leftarrow \tau_{i+j} - \mathbf{t}_{[k_s : k_e]}$ ▷ Compute and store time offsets for response $i + j$
        $\mathbf{X}^{\text{batch}}_{[j,*,*]} \leftarrow \mathbf{X}_{[k_s : k_e, *]}$ ▷ Store windowed predictor sequence for response $i + j$
        $\mathbf{T}^{\text{batch}}_{[j,*]} \leftarrow \mathbf{t}_{[k_s : k_e]}$ ▷ Store windowed predictor timestamps for response $i + j$
        for all $z \in \mathcal{Z}_{i+j}$ **do**
            $\mathbf{V}^{\text{batch}}_{[j,*]} \leftarrow \mathbf{V}^{\text{batch}}_{[j,*]} + \mathbf{1}^{\top} \mathbf{V}_{[*,z]}$ ▷ Accumulate random deviations in the parameters
        end for
    end for
    $\mathbf{V}^{\text{batch}} \leftarrow \mathbf{V}^{\text{batch}} + \mathbf{1}^{\top} \mathbf{v_0}$ ▷ Batched parameters, sum fixed and random effects
    $[\mathbf{U}_{\text{in}} \ \mathbf{U}_{\text{IRF}} \ \mathbf{B} \ \mathbf{S_0}] \leftarrow \mathbf{V}^{\text{batch}}$ ▷ Split $\mathbf{V}^{\text{batch}}$ into its component parameter matrices

    ▷ *Forward pass to compute* $\mathbf{S}$ *(parameters of the response distribution)*
    $\mathbf{S}^{\text{tensor}} \leftarrow f_{\text{in}}(\left[\mathbf{X}^{\text{batch}} \ \mathbf{T}^{\text{batch}}\right]; \mathbf{U}_{\text{in}})$ ▷ Insert timestamps and apply input processing
    $\mathbf{S}^{\text{tensor}} \leftarrow f_{\text{IRF}}\left(\left[\mathbf{S}^{\text{tensor}} \ \mathbf{T}^{\text{batch}} \ \mathbf{D}^{\text{batch}}\right]; \mathbf{U}_{\text{IRF}}\right)$ ▷ Insert timestamps and time offsets and apply IRF
    $\mathbf{S} \leftarrow \sum_{h=1}^{H} \mathbf{S}^{\text{tensor}}{}_{[*,h,*]}$ ▷ Convolve (sum-reduce) along time (H) axis
    $\mathbf{S} \leftarrow \mathbf{S} \odot \mathbf{B}$ ▷ Rescale by Hadamard product with coefficients
    $\mathbf{S} \leftarrow \mathbf{S} + \mathbf{S_0}$ ▷ Sum with base response (intercept $\mathbf{S_0}$)

    ▷ *Update*
    $\ell \leftarrow -\ln P\left(\mathbf{Y}; \mathbf{S}\right)$ ▷ Compute loss, i.e., negative log likelihood
    $\mathbf{g} \leftarrow \nabla\left(\ell, [\mathbf{v_0} \ \mathbf{V}]\right)$ ▷ Compute gradient of loss with respect to parameters
    $[\mathbf{v_0} \ \mathbf{V}] \leftarrow$ optimize($[\mathbf{v_0} \ \mathbf{V}]$, $\mathbf{g}$) ▷ Apply optimizer update to parameters

    $i \leftarrow i + B$
end while

the main article), nor is it the only possible way of implementing a CDR-NN; it is provided merely for expository purposes. As such, **Algorithm 1** is a simplified abstraction that ignores various details, edge cases, and efficiency considerations that an implemented system must contend with. These details are handled by our open-source codebase, which exhaustively describes the implementation (`https://github.com/coryshain/cdr`).

As shown in **Algorithm 1**, we define a *batch* as a subset of B elements of the response variable $\mathbf{Y}$. To enable efficient computation, we require a finite window of H preceding inputs for each response (this allows the time complexity of the convolution step to be constant on dataset size). We compute these windowed inputs for each of the B elements in the batch, resulting in a $B \times H \times K$ tensor of batched predictors (lines 8-13). We also index and sum the random effects associated with each of these B elements, and add them to the fixed effects (lines 14-18). We then apply the sequential transformations that define the CDR-NN: f_{in} (line 21), f_{IRF} (line 22), convolution (summation over the second—H—axis, line 23), rescaling with $\mathbf{B}$ (batched random-effects-corrected $\mathbf{b}$, line 24), and shifting with $\mathbf{S_0}$ (batched random-effects-corrected $\mathbf{s_0}$, line 25). The result is a response distribution parameter matrix $\mathbf{S}$ for the batch, containing a different parameterization for each batch element. The loss is defined as the negative log likelihood of the data $\mathbf{Y}$ given $\mathbf{S}$ (line 27; for simplicity, regularization is omitted) and is used to compute gradients with respect to the parameters $\mathbf{v_0}, \mathbf{V}$ (line 28). The parameters are then updated accordingly (line 29) by an optimization protocol (e.g., the Adam optimizer, as in our implementation; Kingma and Ba, 2014).

D Implementation Details

These analyses use a publicly available implementation of the CDR(NN) framework (`https://github.com/coryshain/cdr`). The system is written in Python TensorFlow (Abadi et al., 2015), an open-source deep learning library. All models reported here use f_{in} = identity, allowing effect interactions and nonlinearities to be handled by f_{IRF}. The model of reference throughout this article uses a feedforward implementation of f_{IRF} with the following key hyperparameters:

- **Hidden layers:** 2
- **Units per hidden layer:** 32
- **L2 weight regularization penalty:** 5
- **L2 random effects regularization penalty:** 10
- **Dropout level:** 0.2
- **Learning rate:** 0.003
- **Batch size:** 1024

Thus, the inputs to the IRF are transformed through two internal feedforward layers (each with 32 artificial neurons) before being projected into the convolution weights $\mathbf{G}_n$. This is a very small network by modern deep learning standards, which we take to be a strength, since it makes the model both parsimonious and fast to train and evaluate. As we show below, the model is nonetheless capable of learning accurate and richly detailed IRFs, and increasing the size of the model provides little gain for the datasets analyzed here. The network is penalized in proportion to the square of its weights of its internal layers, and the random offsets $\mathbf{V}$ are also subject to a shrinkage penalty. This is to improve the reliability of population-level estimates by discouraging the model from using the random effects to capture properties of the group as a whole. For example, if participants fall into two equal groups and models include a fixed effect of *group*, then the *group* variable partitions the set of participants, potentially allowing unpenalized by-participant random effects to completely absorb the effect of *group*. Regularization helps discourage such outcomes.

We apply dropout (Srivastava et al., 2014) after the nonlinearity of each hidden layer. Dropout is a form of regularization that randomly sets neurons of the layer to 0 during training with probability p (per above,

$p = 0.2$ in the reference model) and rescales the remaining units by $1/(1-p)$ to preserve the average magnitude. Dropout approximates model averaging (Srivastava et al., 2014), and it has become a widely-used regularization technique in deep learning. In our case, the use of dropout critically underlies our ability to compute variational Bayesian estimates of uncertainty (see **SI F**).

All models of human subjects data include by-participant random effects, including for intercepts $\mathbf{s_0}$, coefficients $\mathbf{b}$, and IRF parameters. Although in principle each parameter of the neural IRF can have its own by-participant deviation, we found that it was simple, effective, and computationally efficient to restrict by-participant deviation to the bias terms of the internal IRF layers only, leaving all other neural network weights fixed across participants. The network is nonetheless able to capture substantial variation in IRF shape across participants.[4]

Unless otherwise specified, models assume a univariate normal response distribution and are estimated with black box variational Bayes (Ranganath et al., 2014), using a variant of gradient descent (the Adam optimizer, Kingma and Ba 2014) to maximize a regularized log likelihood. We found that explicitly placing a variational prior on the neural network weights and biases led to poor performance (see also e.g., Graves 2011; Gal and Ghahramani 2016). We therefore instead rely on dropout for uncertainty quantification in the deep neural component, since dropout has been shown to variationally approximate a Bayesian deep Gaussian process (Gal and Ghahramani, 2016). Following Shain and Schuler (2021), we additionally place independent normal variational priors on the fixed effects components of coefficients $\mathbf{b}$ and response distribution parameter biases $\mathbf{s}_0$, with standard deviation equal to the standard deviation of the response in the training set. Again following Shain and Schuler (2021), we also place independent normal variational priors on the random effects components of $\mathbf{b}$ and $\mathbf{s}_0$, with standard deviation equal to one tenth the standard deviation of the response in the training set, to encourage shrinkage.

Additional key implementation details common to all models are as follows:

- Adam optimizer (Kingma and Ba, 2014) with default Tensorflow parameters (aside from learning rate, which was directly investigated).
- RNN-internal activations follow LSTM defaults (tanh and sigmoid activations, see Hochreiter and Schmidhuber 1997).
- All other nonlinearities are defined as a computationally efficient approximation to the Gaussian error linear unit (GELU, Hendrycks and Gimpel 2016) used in current state of the art neural language models (Devlin et al., 2019; Radford et al., 2019):

$$\text{GELU}(v) \stackrel{\text{def}}{=} v\,\text{sigmoid}(1.702v) \tag{13}$$

- Models use iterate averaging (Polyak and Juditsky, 1992) with exponential moving average decay rate 0.999 (updates after each minibatch).
- Following Shain and Schuler (2021), for computational efficiency, the predictor history for each response is truncated to a finite number of preceding timesteps (in this study, 128 predictor timesteps, i.e., words). For responses that have fewer than 128 preceding timesteps, the predictor histories are masked to prevent gradient propagation through missing timesteps.
- Following Shain and Schuler (2021), constraints (e.g., variance > 0) are enforced by the softplus function, which has strictly positive output:

$$\text{softplus}(x) \stackrel{\text{def}}{=} \ln(\exp(x) + 1) \tag{14}$$

  Specific initialization values for constrained variables are computed by inverting the constraint to find the value that will yield the target value post-constraint. For example, to initialize a constrained variable

[4]Following Shain and Schuler (2021), by-word random effects were not included to due their tendency to induce overfitting (poor generalization to unseen data) in the cognitive datasets evaluated in this study.

to 1, the inverse softplus is applied to the value 1 to yield a value of approximately 0.54. Choosing this value for the initilization ensures an output value of 1 following application of the constraint function.

- To aid training, all numeric predictors and responses are underlyingly rescaled by their training set standard deviations. These transforms are inverted for evaluation, visualization, and likelihood computation, allowing model estimates to be queried on the original scale.
- Stable training behavior is particularly important in this application, where convergence is diagnosed automatically based on the sequence of losses and users may not always visually inspect learning curves. To this end, these experiments use a number of safeguards against loss spikes, catastrophic forgetting, and numerical instability.
  - The norm of the global gradient is clipped at 1.
  - A constant ϵ = 1e-5 is added to bounded parameters (e.g., standard deviation).
  - The outputs of the convolution $\mathbf{s}$ (eq. 9) are rescaled by $\frac{1}{TK}$ (i.e. divided by the number of timesteps times the number of features), since otherwise poor fit at initialization accumulates over both dimensions during convolution, leading to large early losses and training divergence.
  - Large outlier losses are diagnosed based on de-biased exponential moving averages of the first and second moments of the loss by batch with decay rate 0.999. If the loss at any batch exceeds 1,000 moving standard deviations above the moving mean, training restarts from the last checkpoint.
- 4 CPUs and 1 GPU per training run (specific hardware varied according to availability in our compute resource).

The hyperparameters enumerated above were selected based on exploratory analyses of synthetic and human data with respect to a combination of factors, including parsimony, training speed, exploratory set performance, and consistency of estimates/performance across replicates. No models were evaluated on the test set of any dataset until all analyses were completed.

The same CDR-NN implementation details used for eye-tracking and self-paced reading data work well on the noisier fMRI domain except that the random effects are underregularized, leading to poorer generalization error. We find substantial improvement from increasing the regularizer strength on the random effects, and we therefore define CDR-NN base to use a random effects regularization strength of 1000 rather than 10 in f_{IRF} and a prior over the random effects components of $\mathbf{s_0}$ and $\mathbf{b}$ of one hundredth rather than one tenth the standard deviation of the response in the training set. The fact that different regularization levels are optimal for different datasets is an established pattern in deep learning research. However, the degree of implementational overlap in CDR-NN models across synthetic, reading, and fMRI data suggests that the base hyperparameters are a good starting point that may need little to no tuning when applied to novel domains.

The hyperparameters above define the "base" model in all analyses below. To explore the influence of these hyperparameter choices, we additionally perform a limited grid search over models that deviate (up or down) from the base configuration in one of the following dimensions: number of hidden layers in the IRF, number of units per hidden layer of the IRF, L2 penalty strength on the IRF weights, L2 penalty strength on random IRF effects by participant, dropout level, learning rate, and batch size. We show that results are relatively consistent across a range of parameterizations, suggesting that models are not deeply sensitive to particular choices for these values.

E Extended CDR-NN Model

The simple CDR-NN model defined in the main article assumes independence between impulses in their effects on the response. This assumption is helpful for model interpretation, since the causal effects of input features on estimated responses can be queried directly without reference to context. However, this assumption can also be violated in practice: the response evoked by an impulse may depend in part on

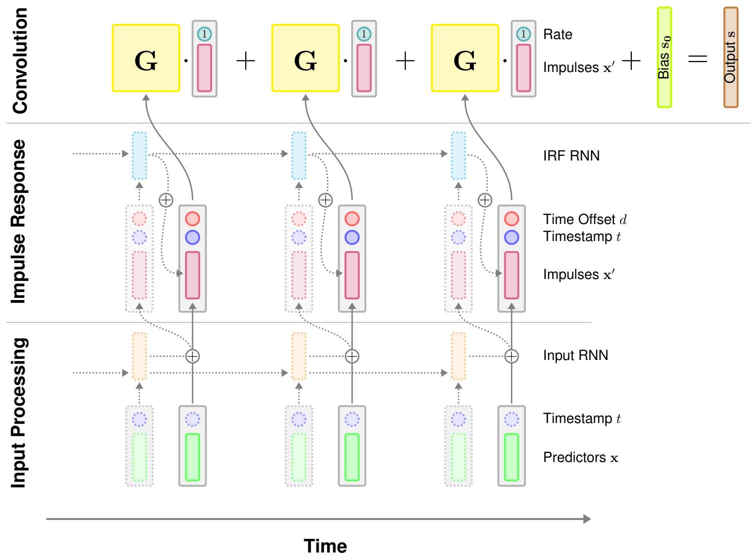

Figure S2: CDR-NN (extended) architecture. A graphical depiction of the extended CDR-NN forward pass (including recurrent connections for context-dependence) for generating one prediction. Scalars are shown as circles, vectors are shown as narrow boxes, matrices are shown as wider boxes, and deep neural network transformations are shown as arrows. Computation proceeds in three stages (bottom-to-top): (i) processing the inputs, (ii) applying the impulse response, and (iii) convolving the impulses with the IRF (convolution weights) over time to generate a parameterization for the predictive distribution over the response. At the convolution stage, the impulses are augmented with bias term (*rate*) that allows the model to capture generalized effects of the rate of events in time. Components shown with dotted lines are not used in the base CDR-NN implementation in this study (although they are explored in the full set of analyses).

the nature and timing of prior impulses. Relaxing this assumption requires a generalized definition of the regression model to allow f_{in} and f_{IRF} to condition on a *sequence* of impulses, rather than on a single impulse, as follows:

$$\mathbf{X}' \stackrel{\text{def}}{=} f_{\text{in}}\left(\begin{bmatrix}\mathbf{t} & \mathbf{X}\end{bmatrix}; \mathbf{u}_{\text{in}}\right) \tag{15}$$

$$\mathbf{G}_1, \ldots, \mathbf{G}_N \stackrel{\text{def}}{=} f_{\text{IRF}}\left(\begin{bmatrix}\mathbf{d} & \mathbf{t} & \mathbf{X}'\end{bmatrix}; \mathbf{u}_{\text{IRF}}\right) \tag{16}$$

This additional flexibility presents a danger to interpretability, since, by conditioning on the entire design matrix, it can leverage context in uninterpretable ways, potentially decoupling the IRF outputs $\mathbf{G}_n$ from their inputs $\mathbf{x}_n$. This property would make it impractical or impossible to reliably query impulse response functions from the model's estimates. There are thus many possible implementations of eq. 16 that would not constitute an interpretable deconvolutional model—e.g., a transformer (Vaswani et al., 2017) implementation of f_{IRF}.

However, as explained below, at least one kind of neural network time series model can relax independence over time while largely retaining a capacity for interpretable IRF estimation: a recurrent neural network (RNN; Elman 1991). An RNN f_{RNN} with transition function r and parameters $\mathbf{w} \in \mathbb{R}^W$ is a time series model that applies recursively to sequential inputs $\mathbf{x}_t, 1 \leq t \leq T$ as follows (where $f_{\text{RNN}}(\mathbf{x}_0)$ is an "initial state", typically $\mathbf{0}$):

$$f_{\text{RNN}}(\mathbf{x}_t) \stackrel{\text{def}}{=} r\left(\mathbf{x}_t, f_{\text{RNN}}(\mathbf{x}_{t-1}); \mathbf{w}\right) \tag{17}$$

Many possible definitions of r have been proposed in the deep learning literature. In this work, r is assumed to be the transition function of a long short-term memory (LSTM) network (Hochreiter and Schmidhuber, 1997).

We incorporate RNNs into the CDR-NN model design as schematized in **Supplementary Figure S2**. As shown, RNNs can be used to introduce context-dependence into either f_{in} or f_{IRF}. In both cases, the RNN

hidden states additively are mixed into the feedforward hidden states, allowing the input processing and/or impulse response functions to change for the same predictor vector as a function of context.

The advantage of an RNN for CDR-NN modeling is that—unlike convolutional neural networks (LeCun et al., 1989) or transformers (Vaswani et al., 2017)—it is *stateful*: all contextual influences on model behavior must be mediated through a fixed dimensional state vector that evolves over time. The IRF can thus be queried from the model *without supplying explicit context* simply by fixing the state at some value. For example, our CDR-NN implementation collects an exponential moving average of RNN hidden state values during training and uses this to parameterize the network for average-case IRF estimation. This procedure results in qualitatively similar average-case IRF estimates and predictive performance to feedforward-only models (**SI G & H**). Thus, we consider our RNN-based approach to be an effective compromise between (i) flexibility in relaxing independence assumptions and (ii) capacity for interpretable IRF estimation.

F Effect Estimation

To query the model's estimates, an input configuration $\mathcal{C} = \langle \mathbf{x}, t_\mathbf{x}, d_\mathbf{x} \rangle$ is constructed and fed to the model, which generates an expected response. By systematically manipulating $\mathcal{C}$ and repeating this procedure, a wide range of estimates can be extracted *post hoc* from the fitted model. For example, to compute the model's estimated baseline IRF (the "deconvolutional intercept", also called *rate* by Shain and Schuler 2021), a reference stimulus $\mathcal{C}_{\text{ref}}$ can be constructed and fed to the model f to yield an estimated response $f(\mathcal{C}_{\text{ref}})$. Stimulus-driven deviation from this reference (e.g., the effect of increasing a predictor by c) can be measured by constructing an alternative configuration $\mathcal{C}_{\text{alt}}$ and computing the difference $f(\mathcal{C}_{\text{alt}}) - f(\mathcal{C}_{\text{ref}})$. Note that f can return any statistic of the response distribution $\mathcal{F}(\mathbf{s})$, including each element of parameter vector $\mathbf{s}$, which the IRF directly models, but also e.g., moments or quantiles. This design therefore provides a highly general procedure for effect estimation. It can be used to run any model query that can be instantiated as a set of input configurations paired with a response statistic of interest, regardless of the internal structure of the model.

Given the nonlinear, interactive, and (in some cases) context-dependent nature of CDR-NN-estimated IRFs, the choice of $\mathcal{C}_{\text{ref}}$ plays an important role in effect estimation: different effects might be obtained relative to different reference inputs. We consider the mean predictor vector (in the training set) to be an appropriate default choice for $\mathcal{C}_{\text{ref}}$, since it constitutes an unbiased estimate of the expected values of the predictors, and we therefore use this approach in all analyses presented here. However, other research questions might motivate a different choice for $\mathcal{C}_{\text{ref}}$. Note that, as in linear models, estimates for nonsensical or unattested predictor values should be treated with caution: the model continuously interpolates/extrapolates as a result of its design, but it is the analyst's responsibility to avoid over-interpreting effects derived from inappropriate regions of predictor space (e.g., estimates for non-integer values of a boolean indicator variable, or estimates outside the range of values attested in the training data). For these reasons, $\mathbf{0}$ is not an appropriate choice for $\mathcal{C}_{\text{ref}}$ in the general case, since depending on the modeling problem it may never be attested in training.

Approximate uncertainty intervals around any effect estimate can be obtained by resampling the model many times from its variational posterior and performing perturbation analysis on each resampled model. This is straightforward for parameters with parametric variational posteriors (e.g., $\mathbf{s_0}$), but it can still be done for the parameters of f_{in} and f_{IRF} as long as the model is fitted using dropout, thanks to Gal and Ghahramani (2016), who showed that resampling models by resampling their dropout masks provides a variational approximation to the posterior of a deep Gaussian process (Damianou and Lawrence, 2013). We therefore resample f_{IRF} by continuing to apply dropout at evaluation time for *post hoc* effect estimation/visualization, which provides a range of possible responses to $\mathcal{C}$ under different dropout masks.[5] Because the intervals thus computed derive from a variational approximating distribution rather than the true posterior, they should not be used for scientific hypothesis testing unless no alternative is feasible.

[5]Note that some queries involve responses at many different values of $\mathcal{C}$, e.g., estimating the IRF shape by evaluating it at many values of $d_\mathbf{x}$. In these cases, the entire query should be run over each resampled model (rather than resampling the model for each point in the query, which will mix multiple models in computing a single sample). Our software implementation enforces this behavior by resampling the model once before each full query.

G Full Results: Synthetic Experiments

Here we present results from the synthetic datasets in Shain and Schuler (2021). **Supplementary Figures S3–S6** vary the signal-to-noise ratio (injected noise with standard deviation 0, 0.1, 1, and 10). **Supplementary Figures S7–S12** vary temporal structure in the data (fixed vs. random intervals between events, short vs. long intervals between events, and synchronous vs. asynchronous predictors and responses). **Supplementary Figures S13–S18** vary pairwise predictor multicollinearity (pairwise correlation level r = 0, 0.25, 0.5, 0.75, 0.9, and 0.95). **Supplementary Figures S19–S21** vary the underlying shape of the true response function (the true kernel is the probability density function of an exponential, normal, or shifted gamma distribution). See Shain and Schuler (2021) for full details about the synthetic data.

Across datasets and model configurations, CDR-NNs accurately recover the ground truth IRFs. Unsurprisingly, certain adverse conditions harm the accuracy of estimates, especially high levels of noise (e.g., error SD = 100) and high levels of predictor multicollinearity (e.g., all pairs of predictors correlated at $r = 0.9$). Unlike the CDR models of Shain and Schuler (2021), which benefited in these evaluations from valid assumptions of IRF kernel family, linearity, stationarity, and homoscedasticity, the CDR-NN models evaluated here had no such foreknowledge, and instead had to learn these features of the response from data. The fact that they do so successfully supports the general applicability of the CDR-NN approach to analyzing continuous-time dynamical systems, even if the full power of a CDR-NN may not be needed for a given modeling task.

Because CDR-NNs are neural networks that stochastically optimize a highly non-convex objective, we additionally use the synthetic datasets to investigate the degree to which estimates differ across replicates of the CDR-NN base model with different random seeds. As shown, estimates are almost indistinguishable in each synthetic task, supporting a high degree of consistency across replicates.

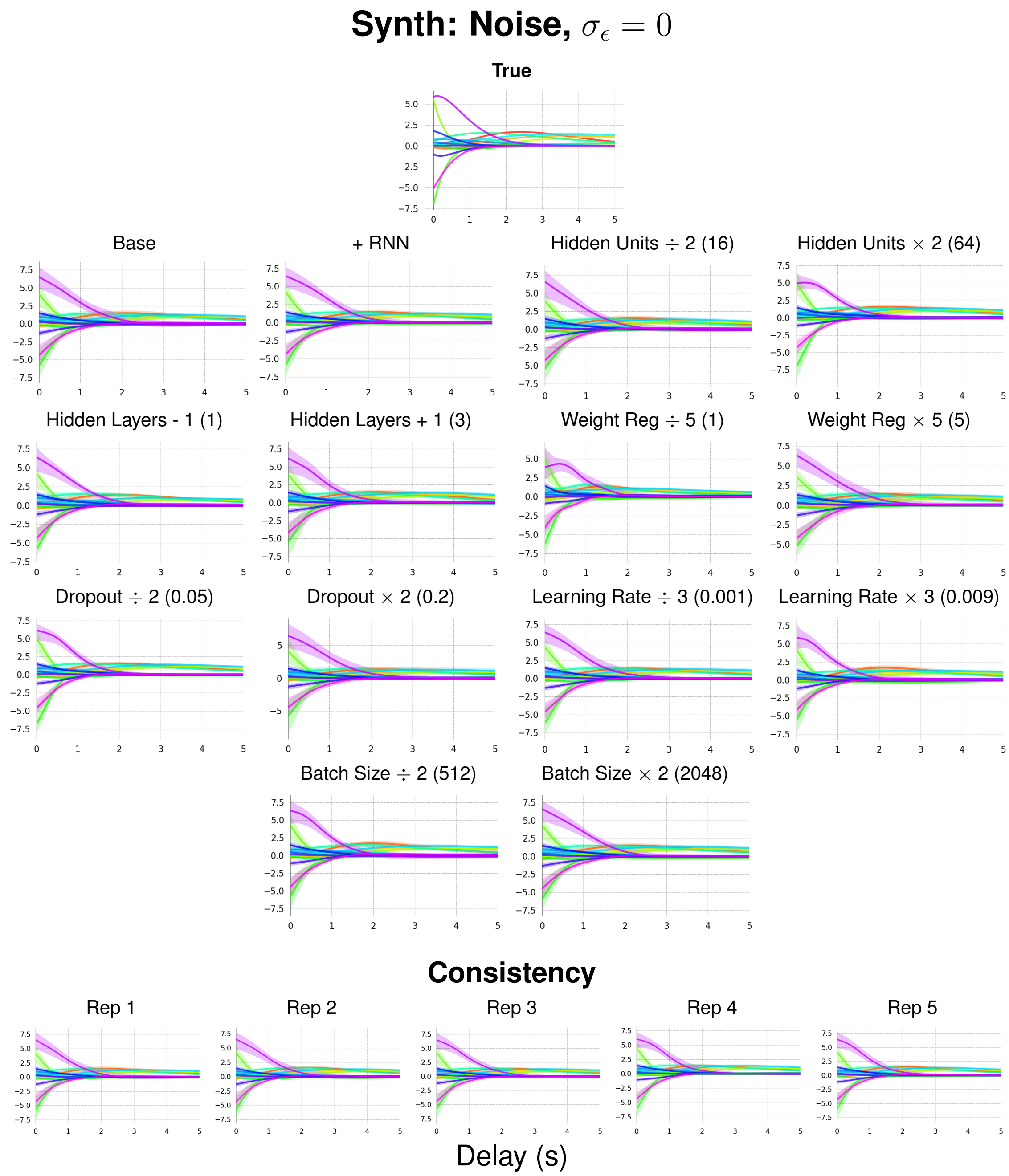

Figure S3: CDR-NN estimated responses to synthetic data with **noise standard deviation** $\sigma_\epsilon = 0$ (i.e. noise free). Estimates using base hyperparameters are compared to estimates from models that deviate from the base in some dimension. Plots under "Consistency" show estimates from five replicates of the "base" configuration, where "Rep 1" is the same model as "base" above, replotted for ease of comparison.

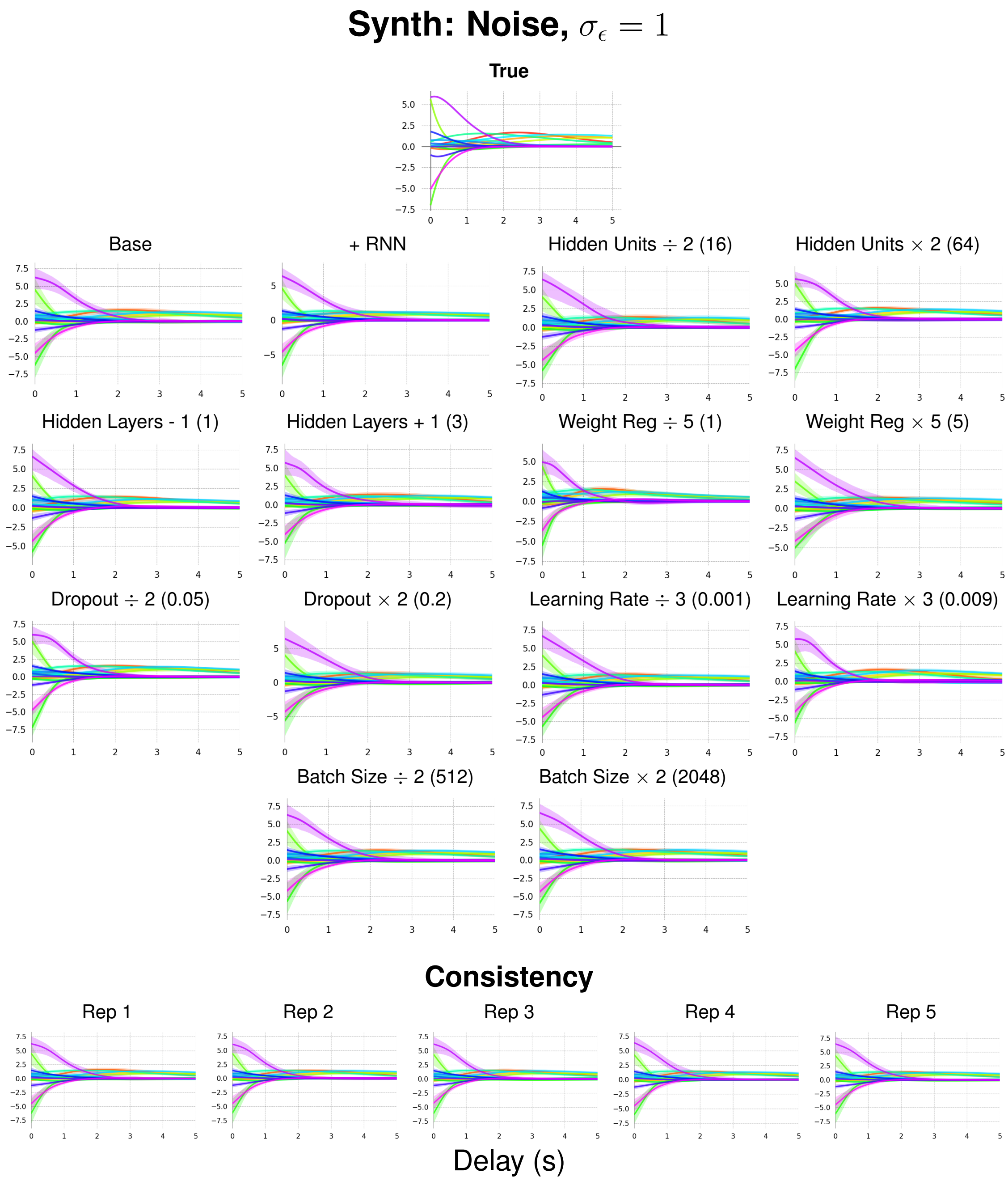

Figure S4: CDR-NN estimated responses to synthetic data with **noise standard deviation** $\sigma_\epsilon = 1$. Estimates using base hyperparameters are compared to estimates from models that deviate from the base in some dimension. Plots under "Consistency" show estimates from five replicates of the "base" configuration, where "Rep 1" is the same model as "base" above, replotted for ease of comparison.

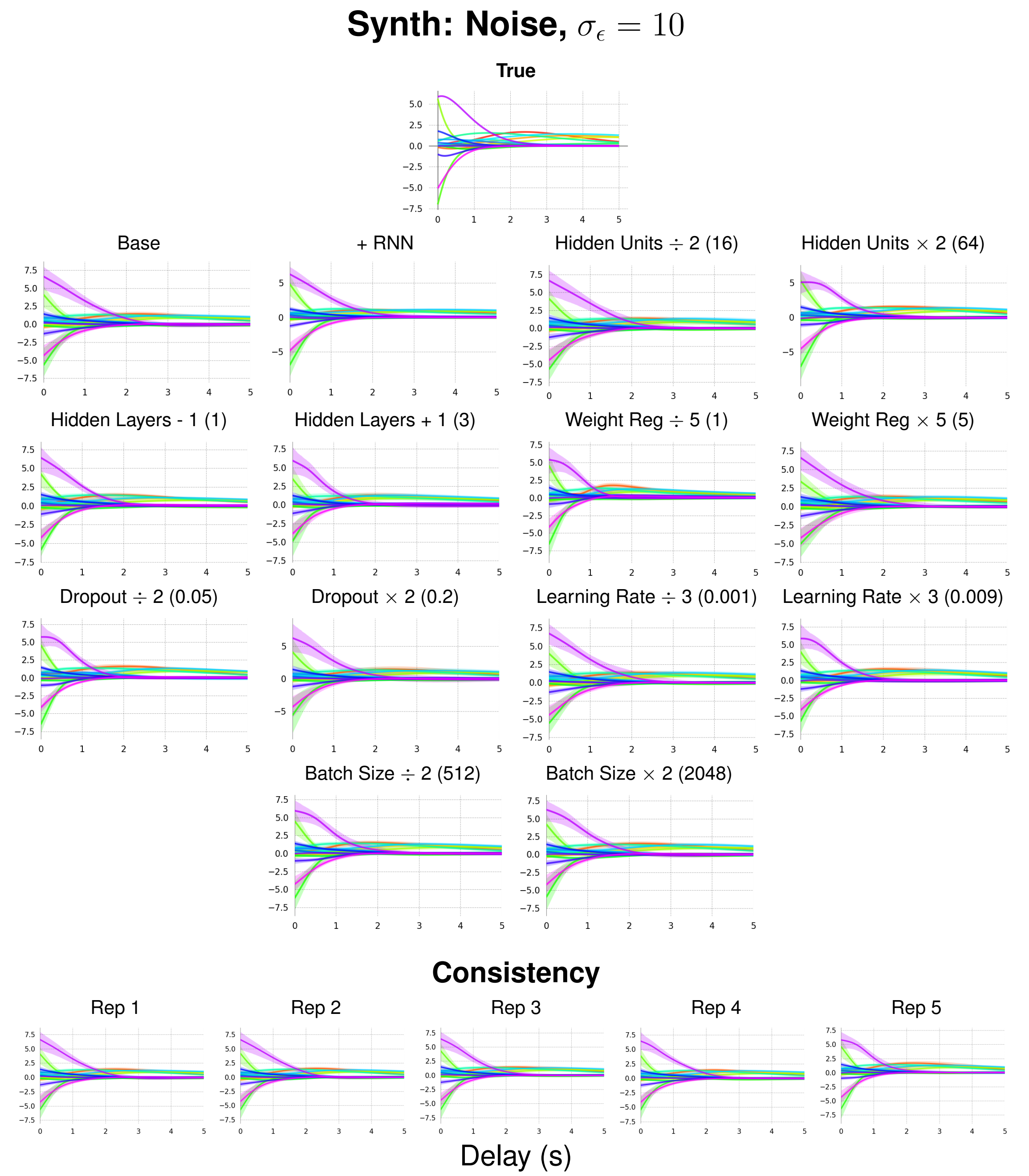

Figure S5: CDR-NN estimated responses to synthetic data with **noise standard deviation** $\sigma_\epsilon = 10$. Estimates using base hyperparameters are compared to estimates from models that deviate from the base in some dimension. Plots under "Consistency" show estimates from five replicates of the "base" configuration, where "Rep 1" is the same model as "base" above, replotted for ease of comparison.

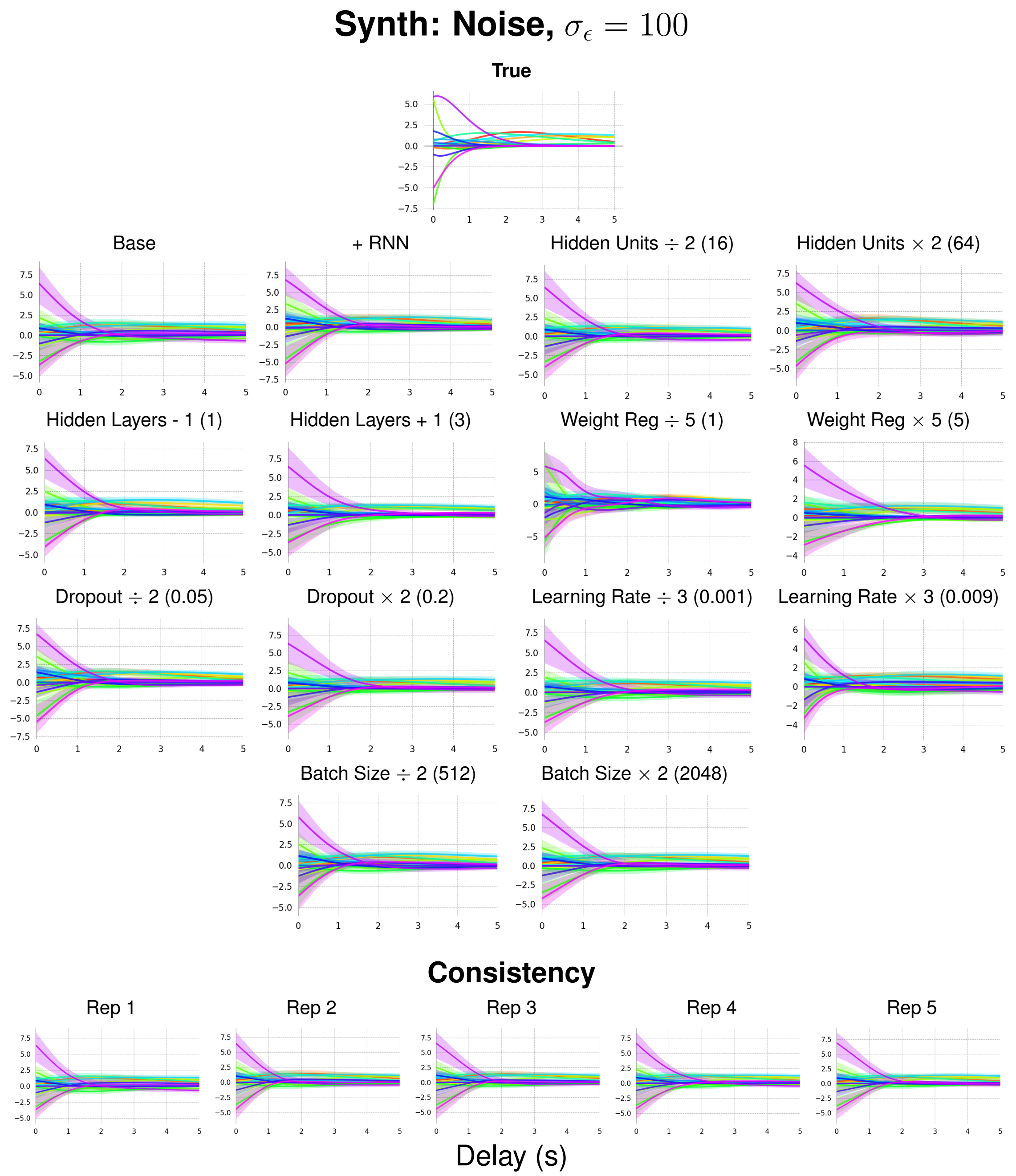

Figure S6: CDR-NN estimated responses to synthetic data with **noise standard deviation** $\sigma_\epsilon = 100$. Estimates using base hyperparameters are compared to estimates from models that deviate from the base in some dimension. Plots under "Consistency" show estimates from five replicates of the "base" configuration, where "Rep 1" is the same model as "base" above, replotted for ease of comparison.

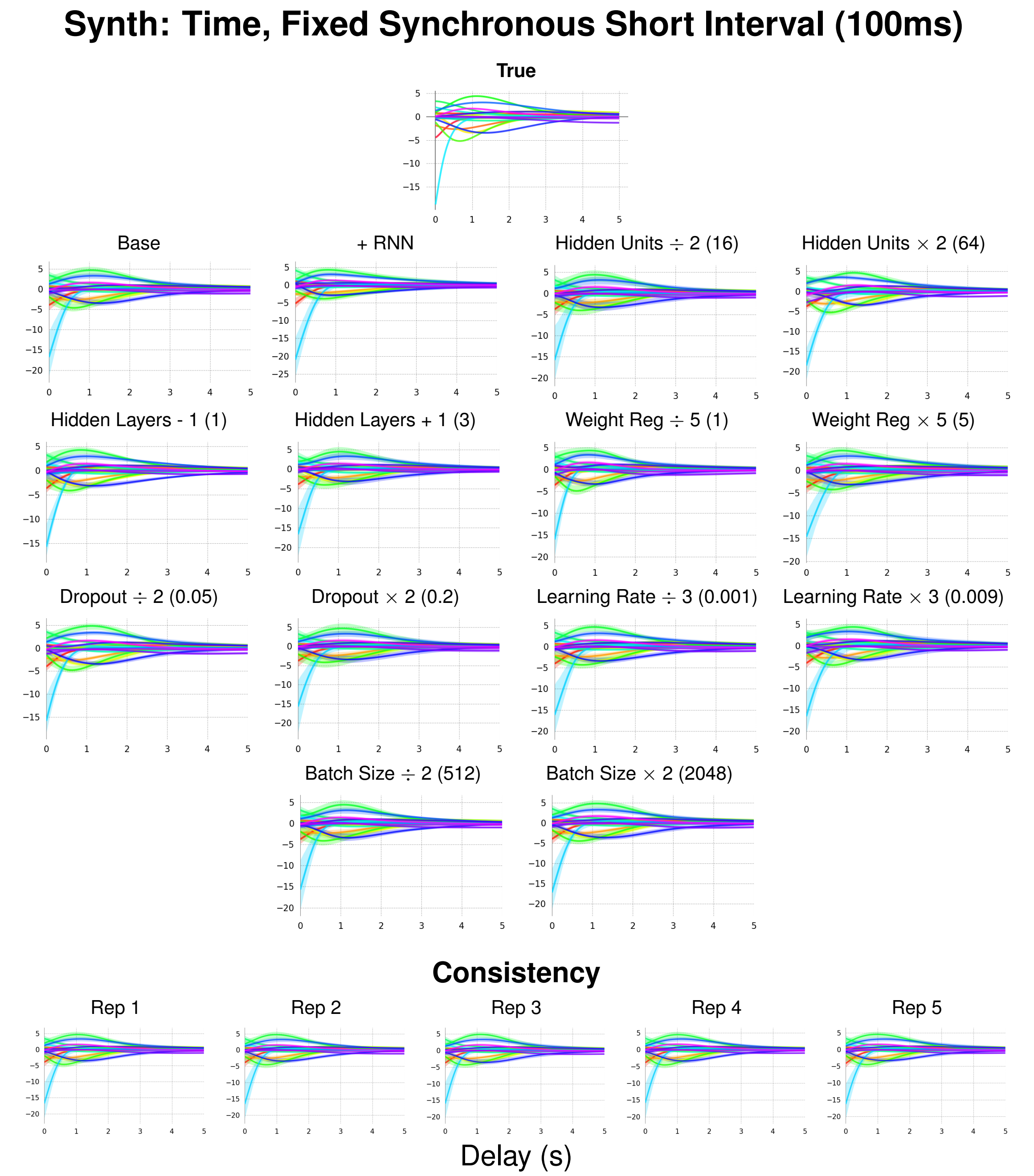

Figure S7: CDR-NN estimated responses to synthetic data with a **synchronously measured predictors and responses with a fixed interval of 100ms between them**. Estimates using base hyperparameters are compared to estimates from models that deviate from the base in some dimension. Plots under “Consistency” show estimates from five replicates of the “base” configuration, where “Rep 1” is the same model as “base” above, replotted for ease of comparison.

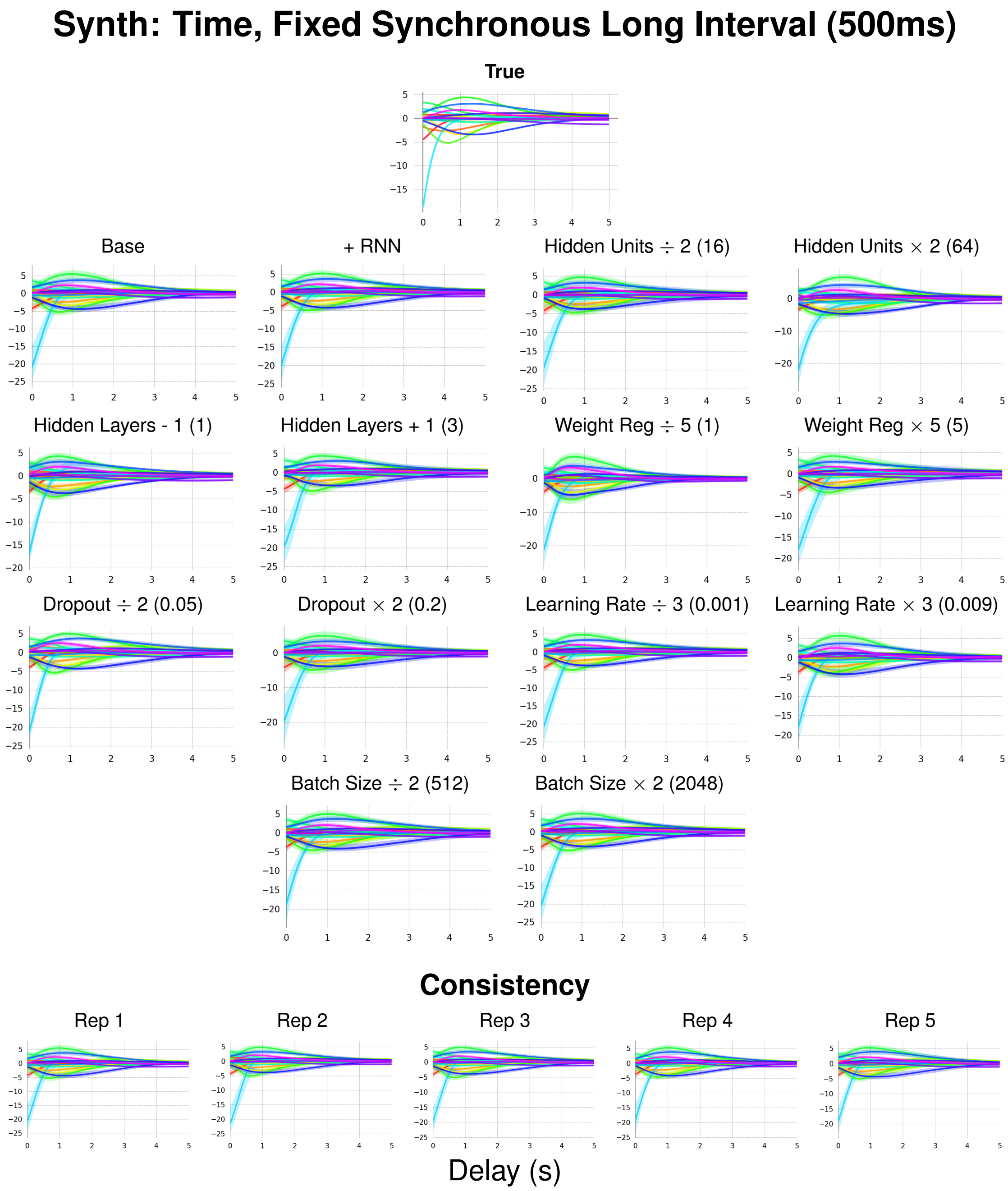

Figure S8: CDR-NN estimated responses to synthetic data with a **synchronously measured predictors and responses with a fixed interval of 500ms between them**. Estimates using base hyperparameters are compared to estimates from models that deviate from the base in some dimension. Plots under “Consistency” show estimates from five replicates of the “base” configuration, where “Rep 1” is the same model as “base” above, replotted for ease of comparison.

Synth: Time, Random Synchronous Short Interval (100ms)

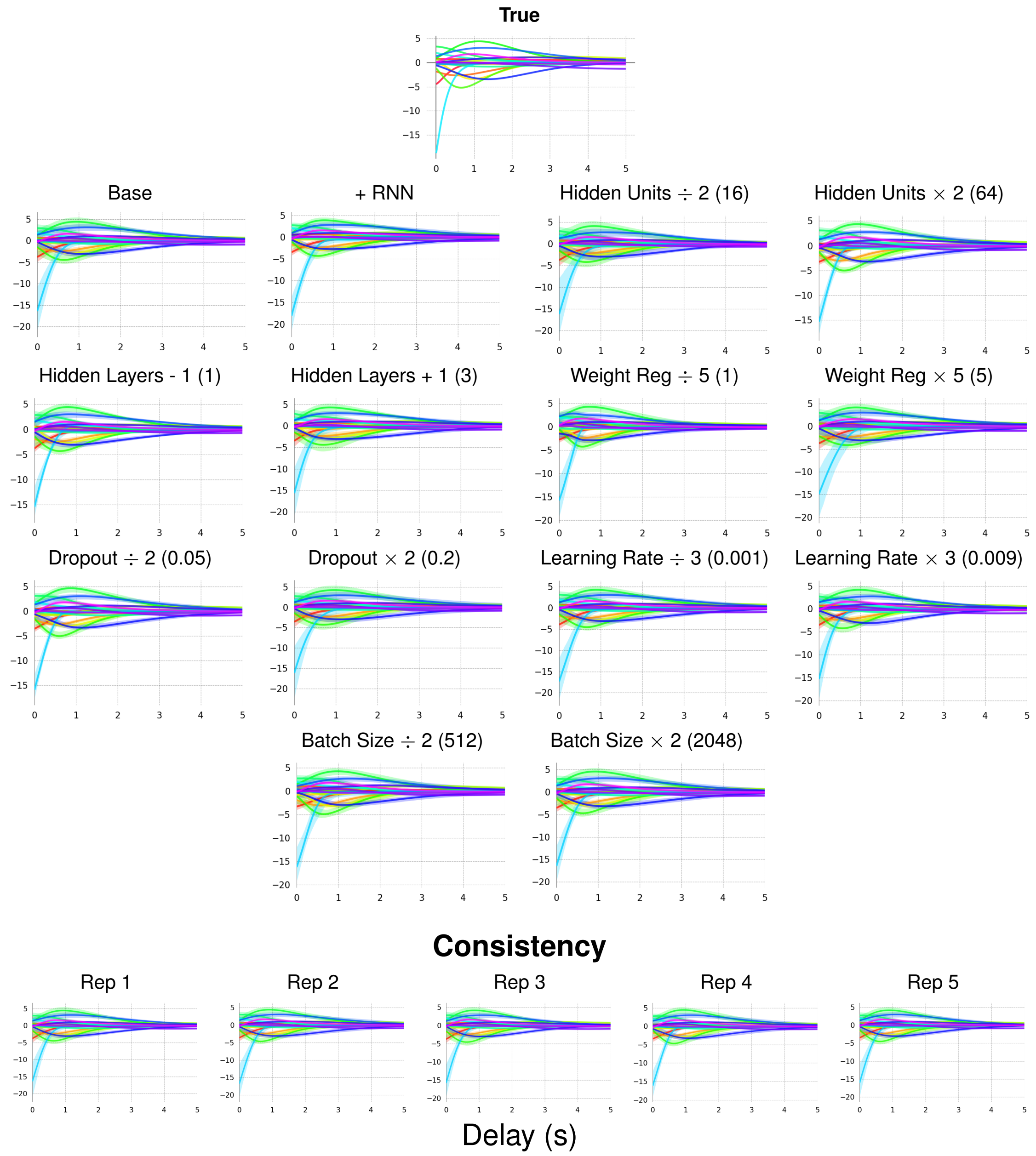

Figure S9: CDR-NN estimated responses to synthetic data with a **synchronously measured predictors and responses with a variable intervals between them (mean interval 100ms)**. Estimates using base hyperparameters are compared to estimates from models that deviate from the base in some dimension. Plots under “Consistency” show estimates from five replicates of the “base” configuration, where “Rep 1” is the same model as “base” above, replotted for ease of comparison.

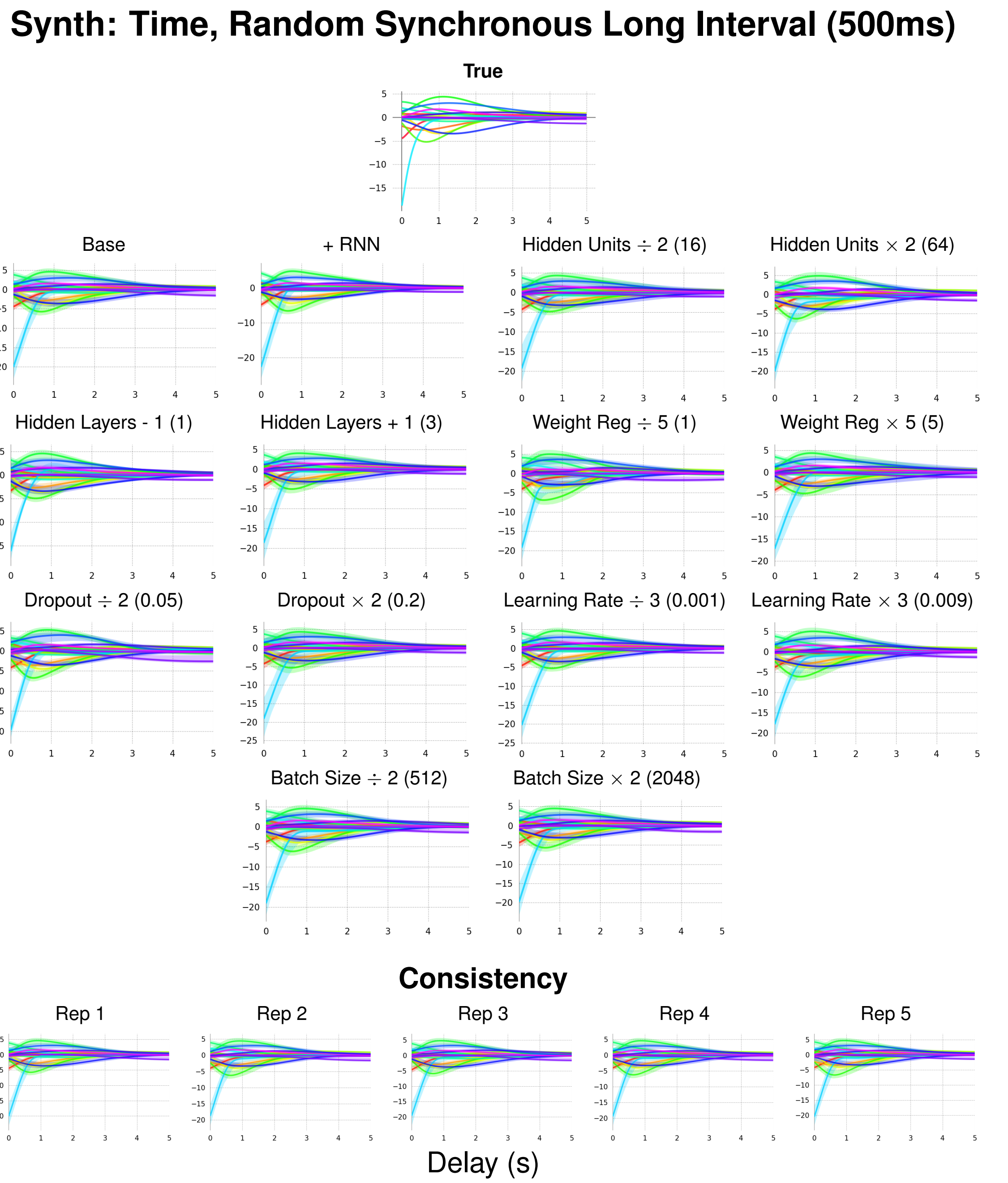

Figure S10: CDR-NN estimated responses to synthetic data with a **synchronously measured predictors and responses with a variable intervals between them (mean interval 500ms)**. Estimates using base hyperparameters are compared to estimates from models that deviate from the base in some dimension. Plots under "Consistency" show estimates from five replicates of the "base" configuration, where "Rep 1" is the same model as "base" above, replotted for ease of comparison.

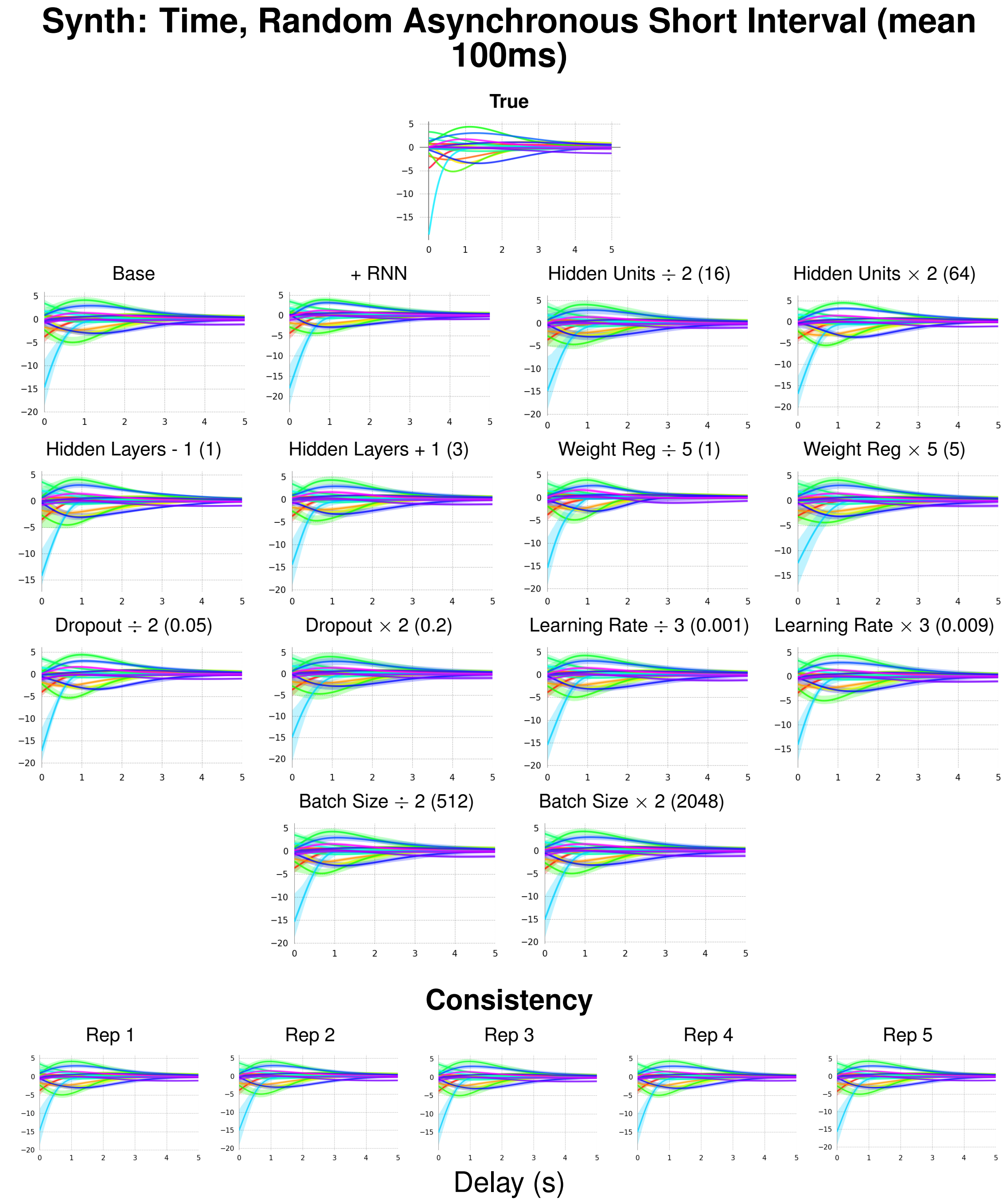

Figure S11: CDR-NN estimated responses to synthetic data with a **asynchronously measured predictors and responses with variable intervals between them (mean interval 100ms)**. Estimates using base hyperparameters are compared to estimates from models that deviate from the base in some dimension. Plots under “Consistency” show estimates from five replicates of the “base” configuration, where “Rep 1” is the same model as “base” above, replotted for ease of comparison.

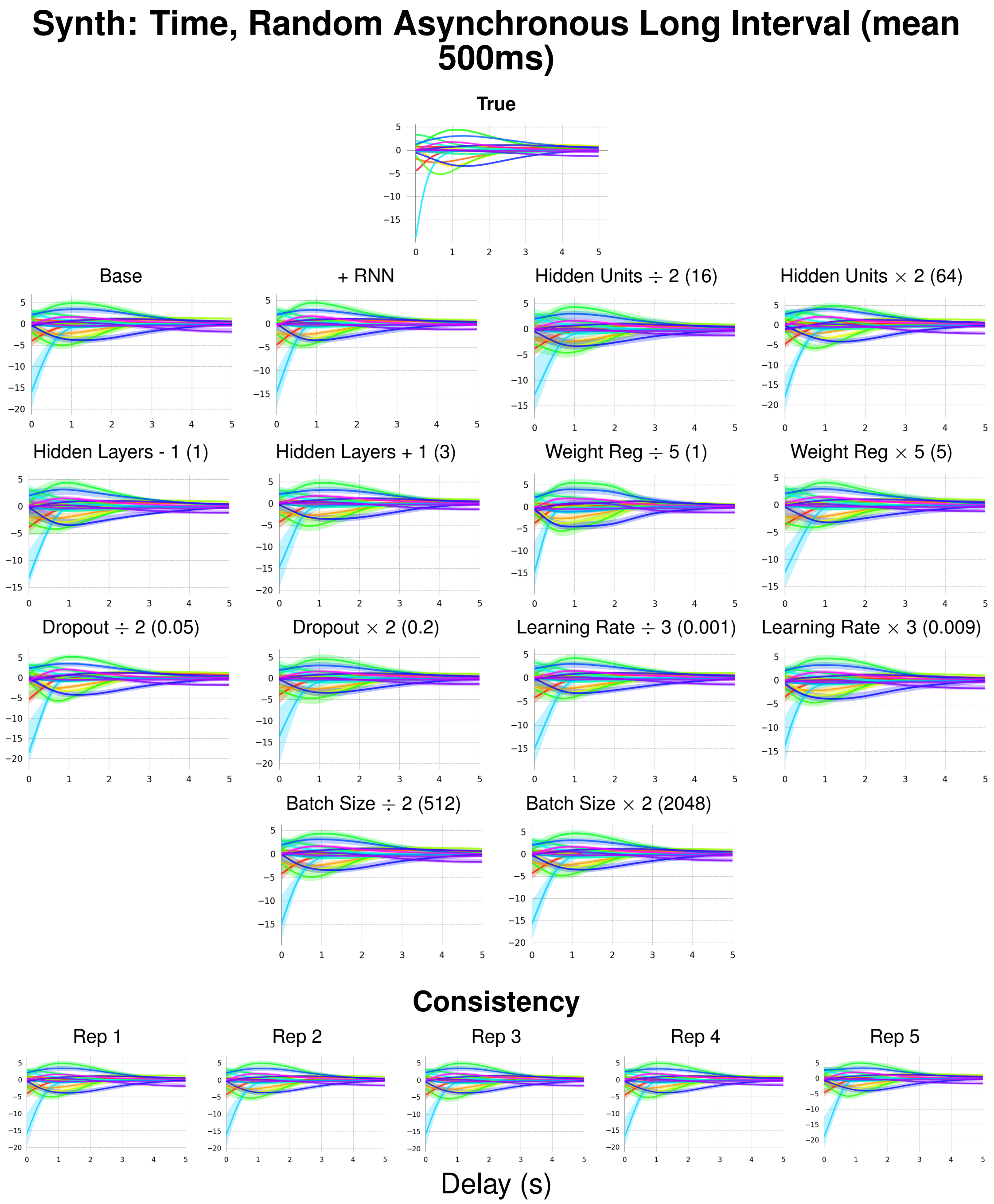

Figure S12: CDR-NN estimated responses to synthetic data with a **asynchronously measured predictors and responses with variable intervals between them (mean interval 500ms)**. Estimates using base hyperparameters are compared to estimates from models that deviate from the base in some dimension. Plots under “Consistency” show estimates from five replicates of the “base” configuration, where “Rep 1” is the same model as “base” above, replotted for ease of comparison.

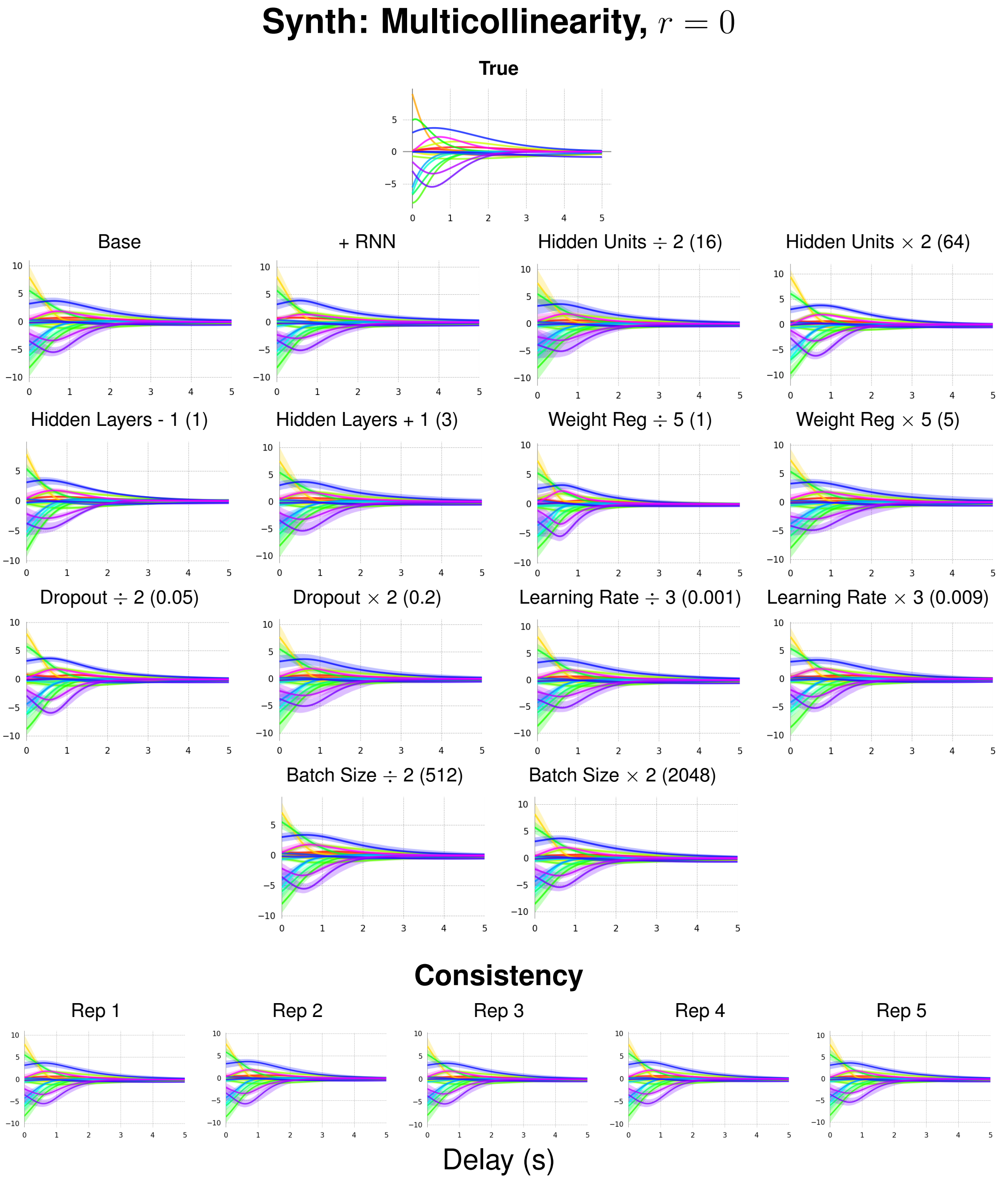

Figure S13: CDR-NN estimated responses to synthetic data with **pairwise predictor multicollinearity of** $r = 0$. Estimates using base hyperparameters are compared to estimates from models that deviate from the base in some dimension. Plots under "Consistency" show estimates from five replicates of the "base" configuration, where "Rep 1" is the same model as "base" above, replotted for ease of comparison.

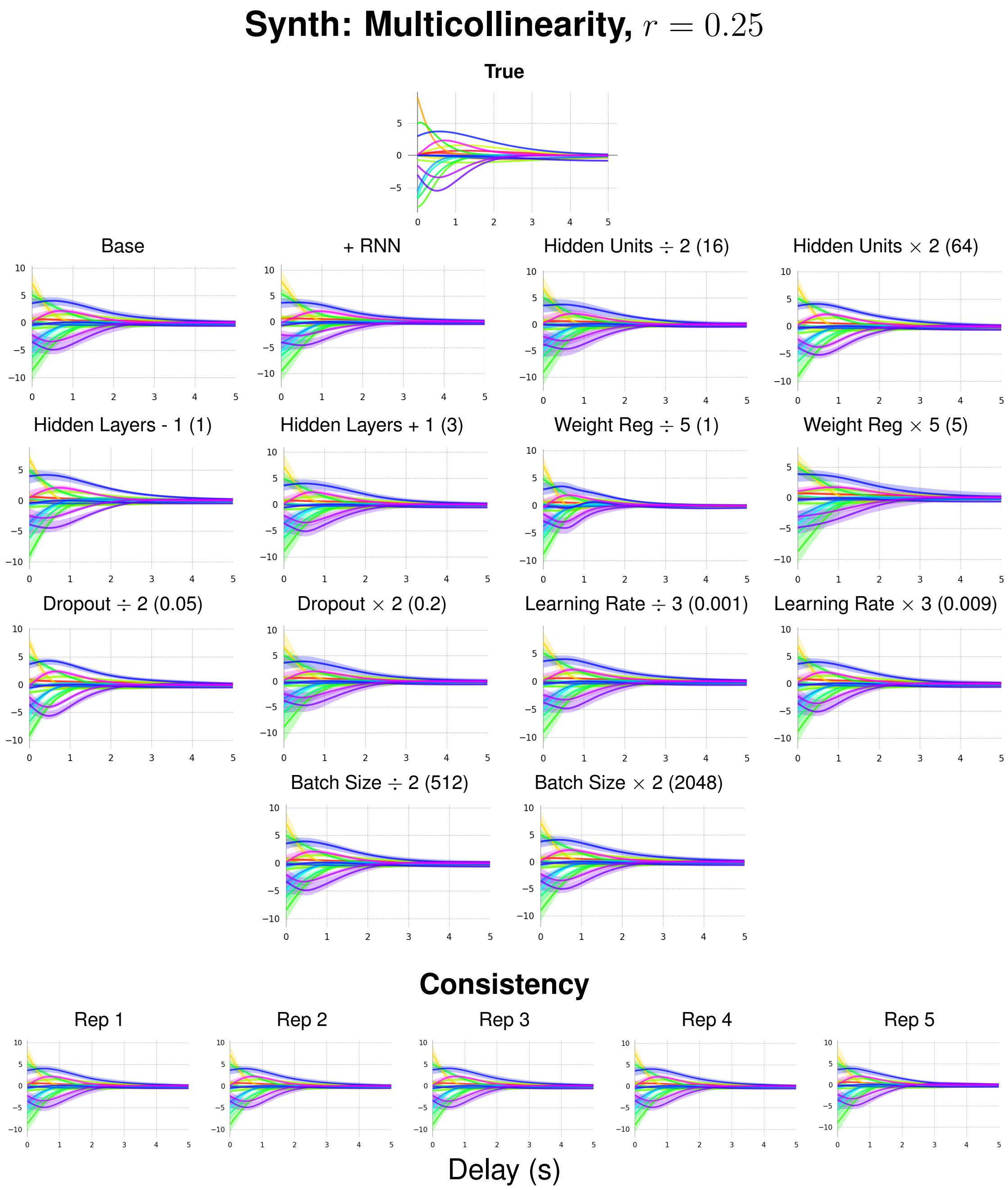

Figure S14: CDR-NN estimated responses to synthetic data with **pairwise predictor multicollinearity of** $r = 0.25$. Estimates using base hyperparameters are compared to estimates from models that deviate from the base in some dimension. Plots under “Consistency” show estimates from five replicates of the “base” configuration, where “Rep 1” is the same model as “base” above, replotted for ease of comparison.

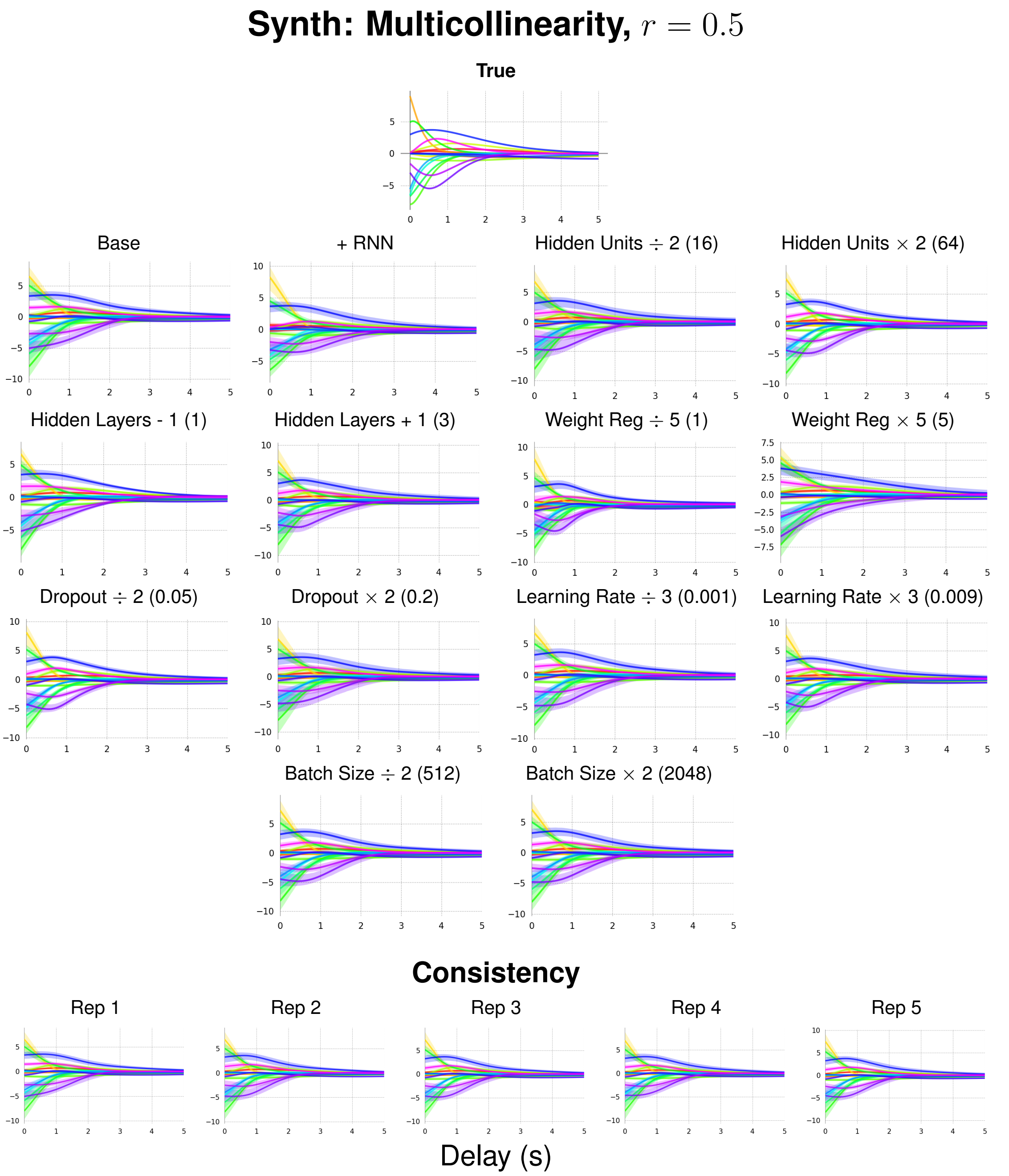

Figure S15: CDR-NN estimated responses to synthetic data with **pairwise predictor multicollinearity of** $r = 0.5$. Estimates using base hyperparameters are compared to estimates from models that deviate from the base in some dimension. Plots under "Consistency" show estimates from five replicates of the "base" configuration, where "Rep 1" is the same model as "base" above, replotted for ease of comparison.

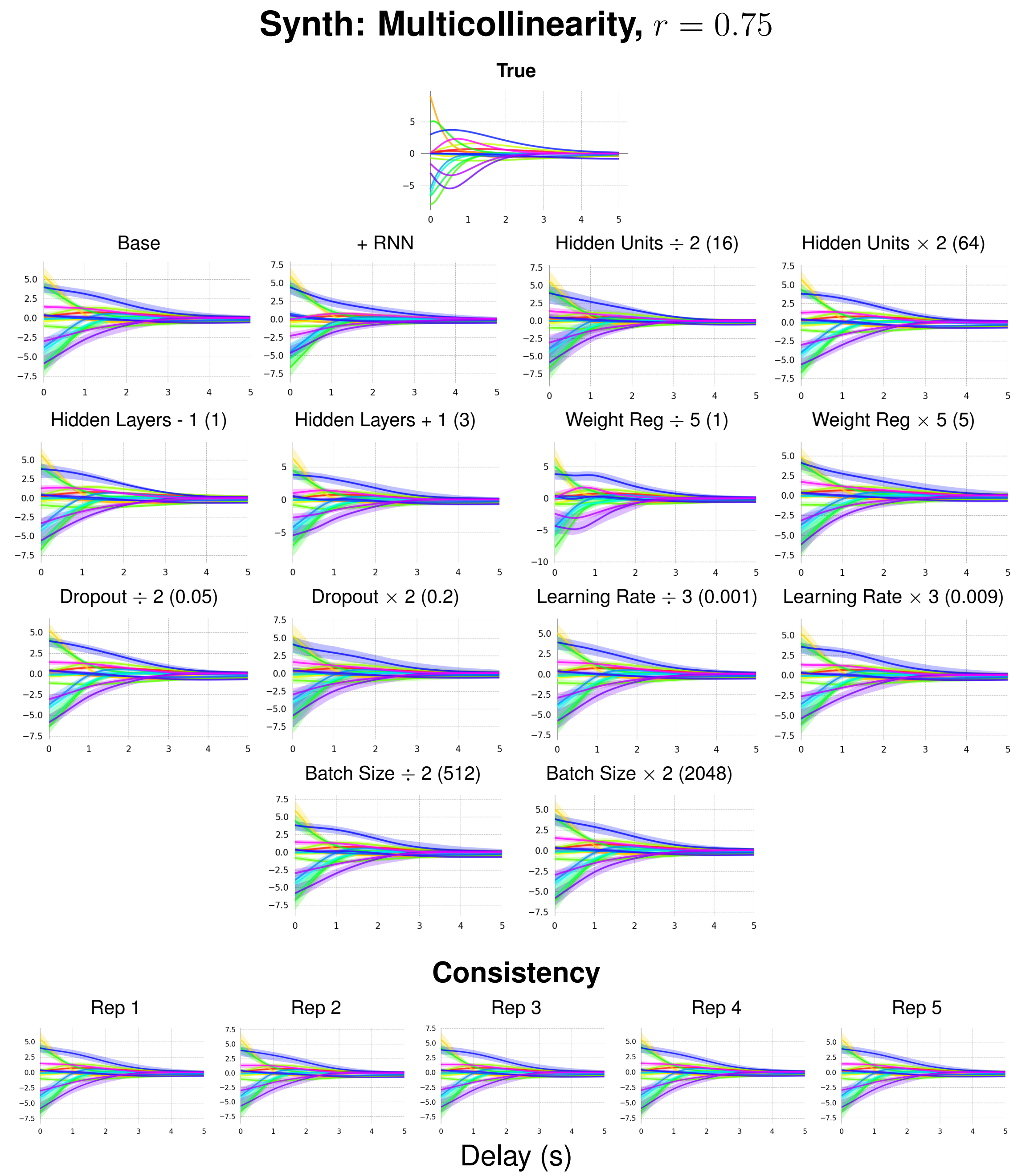

Figure S16: CDR-NN estimated responses to synthetic data with **pairwise predictor multicollinearity of** $r = 0.75$. Estimates using base hyperparameters are compared to estimates from models that deviate from the base in some dimension. Plots under "Consistency" show estimates from five replicates of the "base" configuration, where "Rep 1" is the same model as "base" above, replotted for ease of comparison.

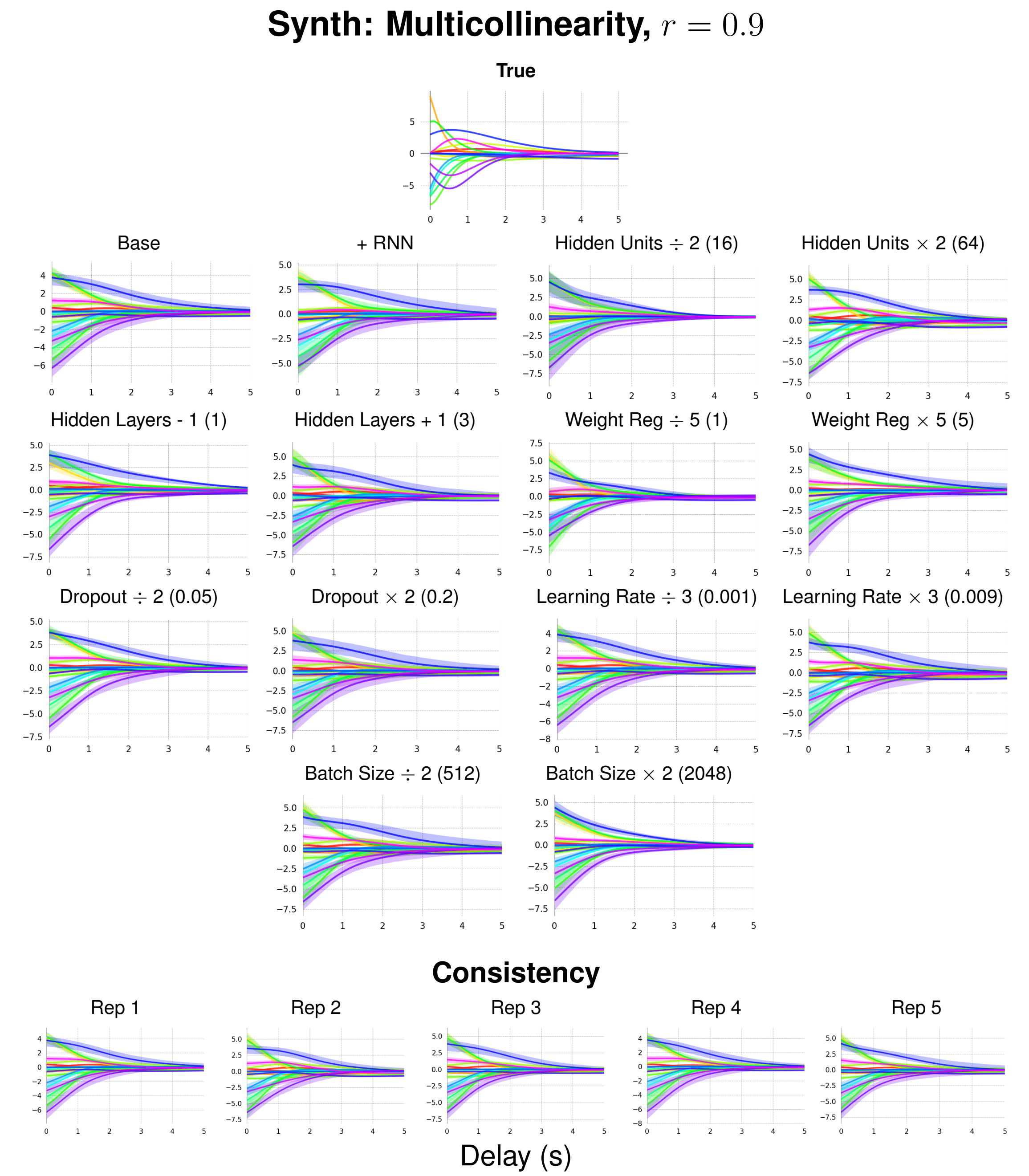

Figure S17: CDR-NN estimated responses to synthetic data with **pairwise predictor multicollinearity of** $r = 0.9$. Estimates using base hyperparameters are compared to estimates from models that deviate from the base in some dimension. Plots under “Consistency” show estimates from five replicates of the “base” configuration, where “Rep 1” is the same model as “base” above, replotted for ease of comparison.

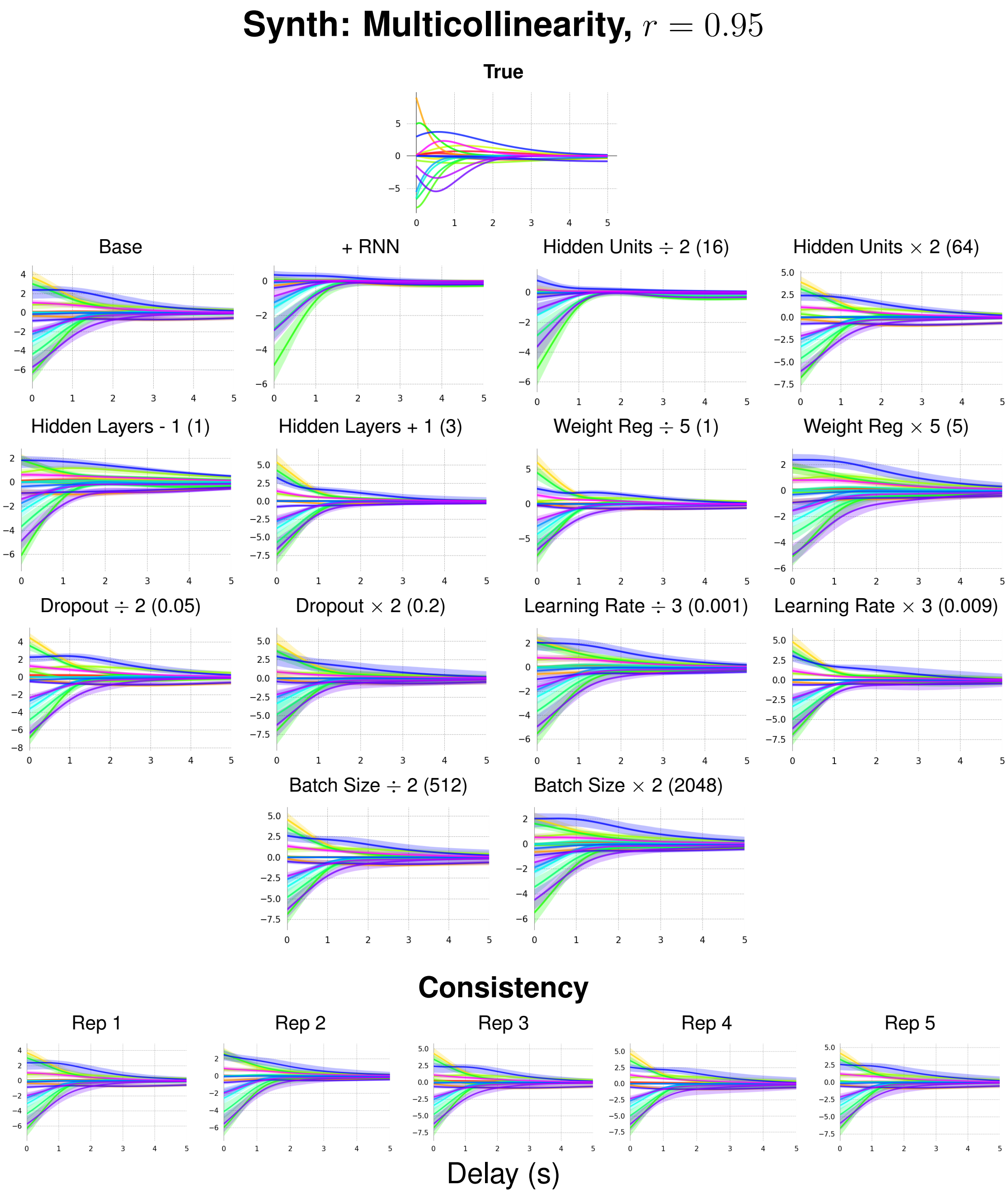

Figure S18: CDR-NN estimated responses to synthetic data with **pairwise predictor multicollinearity of** $r = 0.95$. Estimates using base hyperparameters are compared to estimates from models that deviate from the base in some dimension. Plots under “Consistency” show estimates from five replicates of the “base” configuration, where “Rep 1” is the same model as “base” above, replotted for ease of comparison.

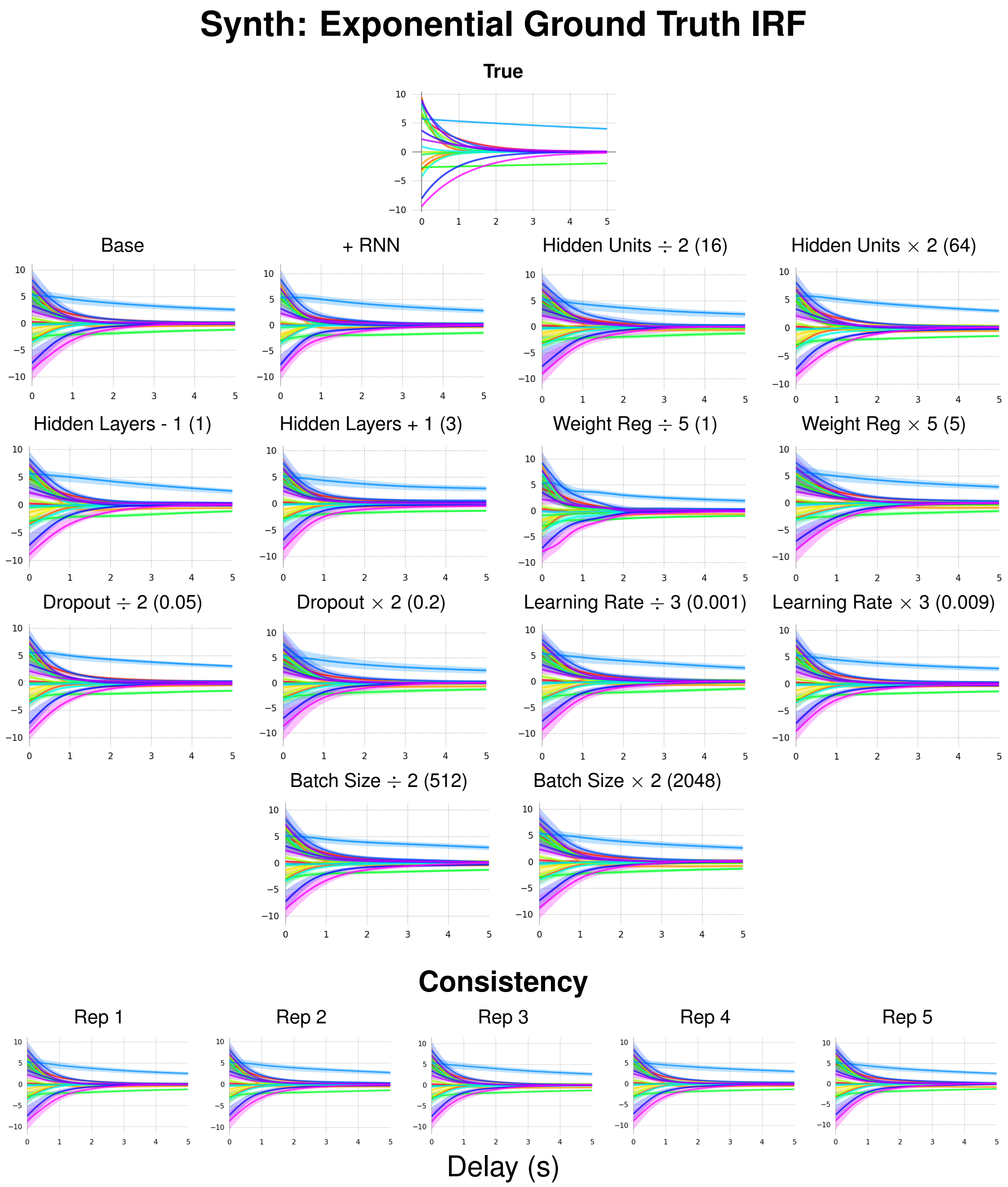

Figure S19: CDR-NN estimated responses to synthetic data convolved using an **exponential IRF**. Estimates using base hyperparameters are compared to estimates from models that deviate from the base in some dimension. Plots under "Consistency" show estimates from five replicates of the "base" configuration, where "Rep 1" is the same model as "base" above, replotted for ease of comparison.

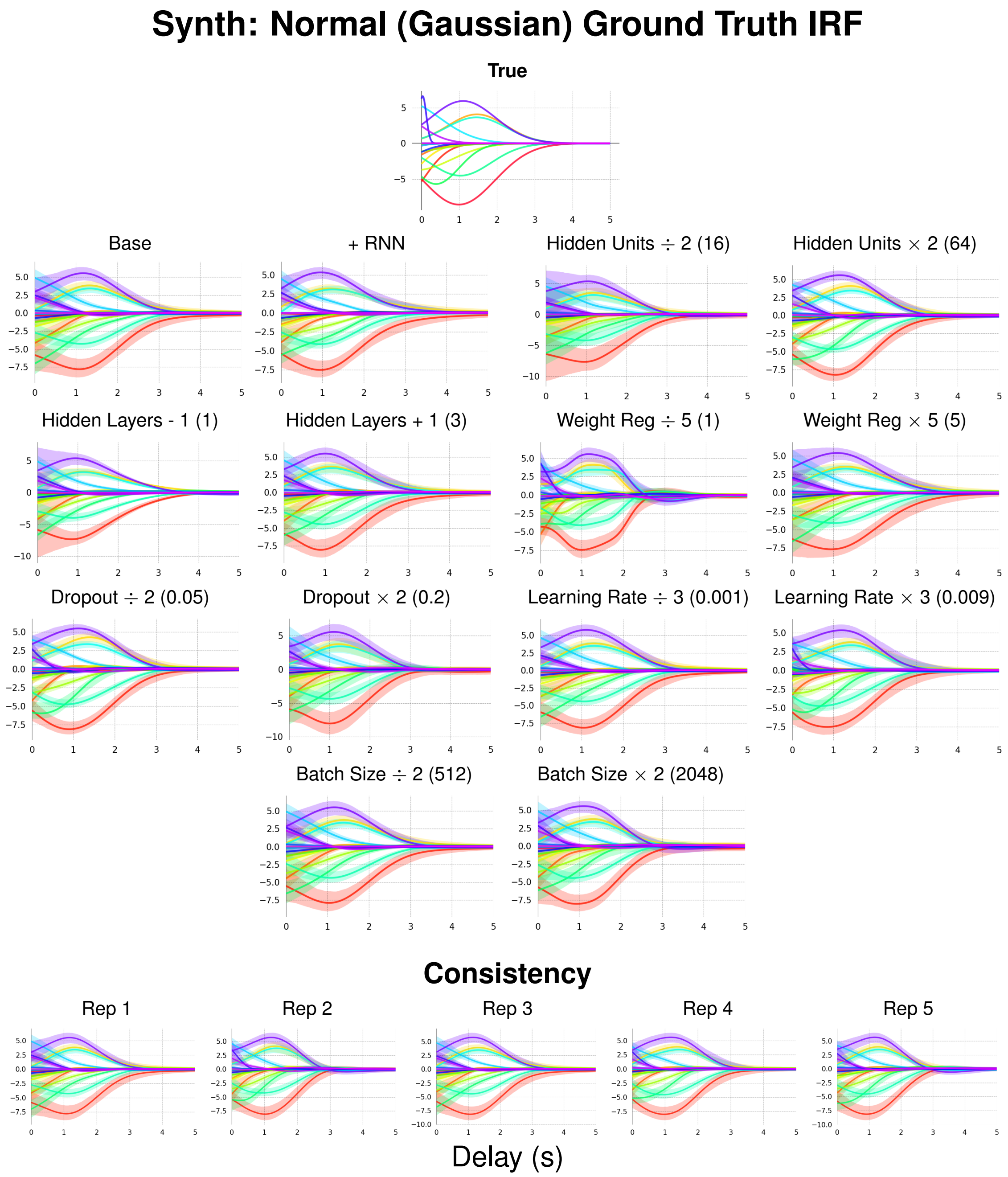

Figure S20: CDR-NN estimated responses to synthetic data convolved using a **normal (Gaussian) IRF**. Estimates using base hyperparameters are compared to estimates from models that deviate from the base in some dimension. Plots under "Consistency" show estimates from five replicates of the "base" configuration, where "Rep 1" is the same model as "base" above, replotted for ease of comparison.

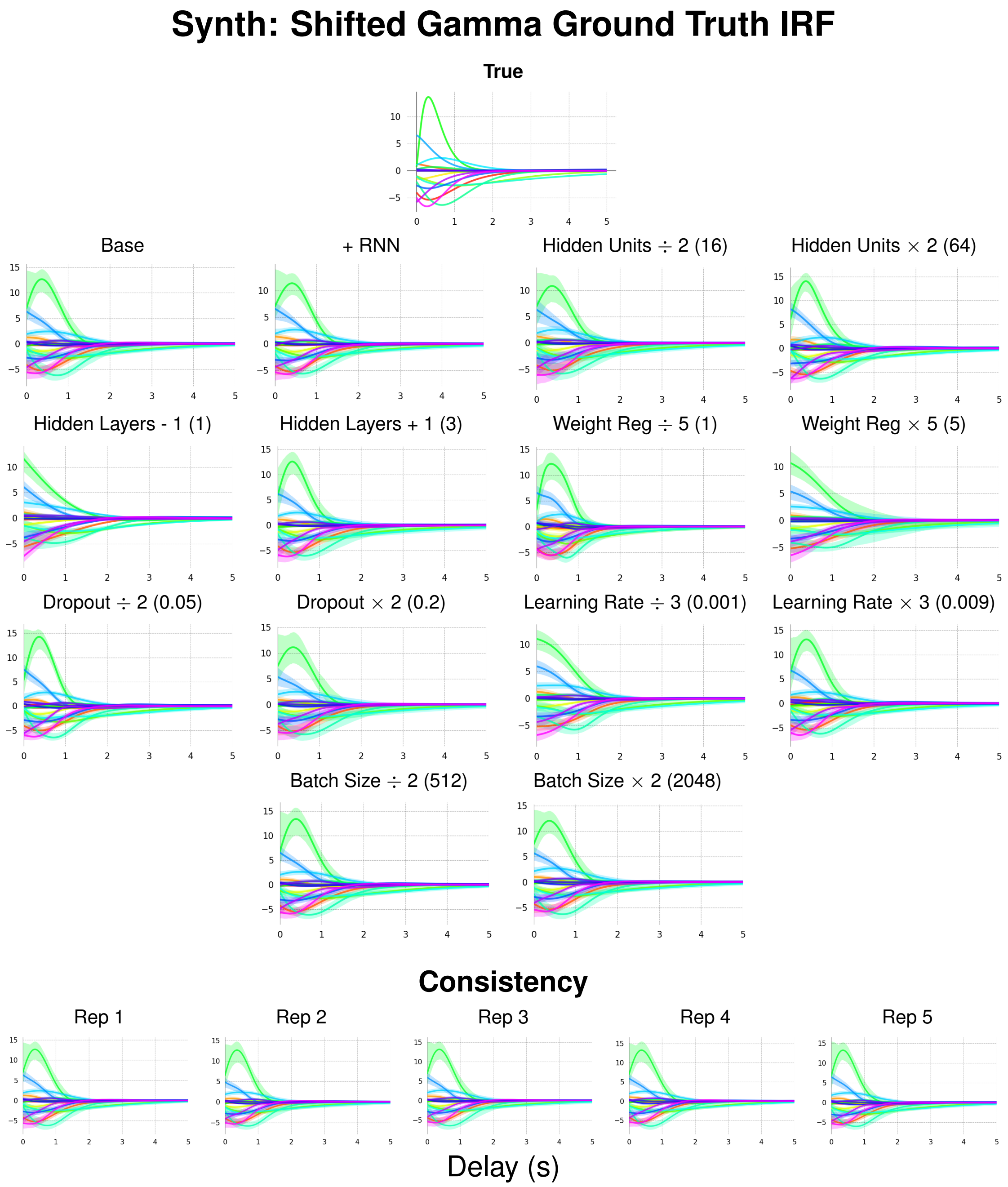

Figure S21: CDR-NN estimated responses to synthetic data convolved using a **Shifted Gamma IRF**. Estimates using base hyperparameters are compared to estimates from models that deviate from the base in some dimension. Plots under "Consistency" show estimates from five replicates of the "base" configuration, where "Rep 1" is the same model as "base" above, replotted for ease of comparison.

	Dundee Scan Path Duration (ms)			Dundee Scan Path Duration (log ms)		
Model	Train	Expl	Test	Train	Expl	Test
LME	-779299†	-391437†	-391996†	-70203†	-35380†	-35331†
LME-S	—	—	—	—	—	—
GAM	-778411	-391105	-391653	-69660	-35169	-35151
GAM-S	-777822	-390867	-391461	-68889	-34839	-34840
GAMLSS	-769269	-387376	-387433	-65818	-33319	-33341
GAMLSS-S	*-766609*	*-386190*	*-387617*	*-65203*	*-33065*	*-33130*
CDR	-778839	-391217	-391891	-69559	-35066	-35118
CDR-NN base	-759754	-385571	**-386209**	-62253	-31809	**-31832**
–Nonlinear	-764022	-385926	—	-63625	-32488	—
–Nonstationary	-760876	-384995	—	-62414	-31858	—
–Heteroscedastic	-777779	-390825	—	-68305	-34588	—
+RNN	-756290	-385808	—	-61663	-31765	—
Units ÷ 2 (16)	-762452	-384804	—	-63172	-32168	—
Units × 2 (64)	***-755227***	-386670	—	-62006	-31770	—
Layers - 1 (1)	-760365	-384948	—	-62841	-32178	—
Layers + 1 (3)	-756745	-385449	—	-62689	-31963	—
Weight Reg ÷ 5 (1)	-756150	-386446	—	-61631	-31900	—
Weight Reg × 5 (25)	-764699	-384783	—	-63320	-32141	—
Ranef Reg ÷ 10 (1)	-758189	-386038	—	-61740	-31896	—
Ranef Reg × 10 (100)	-760369	-385477	—	-62212	***-31750***	—
Dropout ÷ 2 (0.05)	-755535	-387555	—	***-61508***	-31826	—
Dropout × 2 (0.2)	-766793	-386002	—	-64406	-32649	—
Learning Rate ÷ 3 (0.001)	-758376	-386336	—	-62048	-31998	—
Learning Rate × 3 (0.009)	-762212	***-384560***	—	-62661	-31898	—
Batch Size ÷ 2 (512)	-760408	-384876	—	-62282	-31881	—
Batch Size × 2 (2048)	-758487	-384933	—	-62168	-31879	—

Table S1: Dundee (scan path duration likelihood). Log likelihood from CDR-NN vs. linear mixed-effects (LME), generalized additive model (GAM), and generalized additive model for location, scale, and shape (GAMLSS) baselines with and without three additional spillover positions (-S) to help capture delayed effects, as well as kernel-based (non-neural) CDR (LME, GAM, and CDR performance as reported in Shain and Schuler 2021). LME baselines show the marginal likelihood for the training set (the default likelihood implemented by the `lme4` package). All other likelihoods are conditional on the fitted model. Estimates from LME-S are omitted because training exceeded the two-week maximum runtime permitted by our compute resource. CDR-NN variants add recurrence (+RNN) and modify the number of Units (Units), number of hidden layers (Layers), weight regularization strength (Reg), random effects regularization strength (RanReg), dropout rate (Dropout), learning rate (LR), and batch size (Batch). Of the CDR-NN models, only CDR-NN base is evaluated on the test set. Best-performing models within the sets of baseline and CDR-NN models are shown in *italics*. Best-performing overall models are shown in **bold**. Daggers (†) indicate convergence failures.

H Full Results: Cognitive Experiments

Here we present the full results from all cognitive datasets evaluated in this study: Dundee (eye-tracking, ET), Natural Stories (self-paced reading, SPR), and Natural Stories (fMRI).

H.1 Dundee (Eye-Tracking)

In-sample and out-of-sample predictive performance on scan path, first pass, and go-past durations in Dundee are given in **Supplementary Tables S1–S3**. Effect estimates in Dundee are plotted in **Supplementary Figures S22–S27**. Estimates are plausible and highly consistent across model variants.

Model	Dundee First Pass Duration (ms)			Dundee First Pass Duration (log ms)		
	Train	Expl	Test	Train	Expl	Test
LME	-581489†	-292478†	-292359†	-45022	-22847	-22689
LME-S	-581426†	-292408†	-292357†	-44707†	-22694†	-22603†
GAM	-580851	-292246	-292097	-44706	-22744	-22601
GAM-S	-580394	-292048	-291926	-44115	-22479	-22334
GAMLSS	-572205	-288244	*-288091*	-42622	-21720	-21588
GAMLSS-S	*-571176*	*-288228*	-288270	*-42117*	*-21529*	*-21416*
CDR	-581093	-292368	-292287	-44560	-22805	-22514
CDR-NN base	-568093	-287574	**-287588**	-40389	-20736	**-20655**
–Nonlinear	-571742	-288394	—	-41358	-21139	—
–Nonstationary	-567573	-287672	—	-40313	-20726	—
–Heteroscedastic	-579919	-292049	—	-43886	-22348	—
+RNN	-565905	-288309	—	-39750	***-20604***	—
Units ÷ 2 (16)	-569042	-288025	—	-40743	-20838	—
Units × 2 (64)	-565389	-287831	—	-40387	-20732	—
Layers - 1 (1)	-568460	-287740	—	-40335	-20863	—
Layers + 1 (3)	-568386	-287866	—	-40414	-20730	—
Weight Reg ÷ 5 (1)	-564244	-288287	—	***-39712***	-20722	—
Weight Reg × 5 (25)	-569491	***-287459***	—	-40913	-20901	—
Ranef Reg ÷ 10 (1)	***-564069***	-288306	—	-39734	-20706	—
Ranef Reg × 10 (100)	-566034	-288112	—	-40328	-20747	—
Dropout ÷ 2 (0.05)	-565890	-287747	—	-39924	-20707	—
Dropout × 2 (0.2)	-570617	-287467	—	-41311	-21007	—
Learning Rate ÷ 3 (0.001)	-568584	-287635	—	-40174	-20715	—
Learning Rate × 3 (0.009)	-569433	-287480	—	-40800	-20852	—
Batch Size ÷ 2 (512)	-569016	-287829	—	-40497	-20788	—
Batch Size × 2 (2048)	-567679	-288099	—	-40083	-20685	—

Table S2: Dundee (first pass duration likelihood). Log likelihood from CDR-NN vs. linear mixed-effects (LME), generalized additive model (GAM), and generalized additive model for location, scale, and shape (GAMLSS) baselines with and without three additional spillover positions (-S) to help capture delayed effects, as well as kernel-based (non-neural) CDR (LME, GAM, and CDR performance as reported in Shain and Schuler 2021). LME baselines show the marginal likelihood for the training set (the default likelihood implemented by the `lme4` package). All other likelihoods are conditional on the fitted model. CDR-NN variants add recurrence (+RNN) and modify the number of Units (Units), number of hidden layers (Layers), weight regularization strength (Reg), random effects regularization strength (RanReg), dropout rate (Dropout), learning rate (LR), and batch size (Batch). Of the CDR-NN models, only CDR-NN base is evaluated on the test set. Best-performing models within the sets of baseline and CDR-NN models are shown in *italics*. Best-performing overall models are shown in **bold**. Daggers (†) indicate convergence failures.

Model	Dundee Go-Past Duration (ms) Train	Expl	Test	Dundee Go-Past Duration (log ms) Train	Expl	Test
LME	-636316	-315308†	-317511†	-59556†	-29690†	-29689†
LME-S	-636199†	-315280†	-317546†	-59220†	-29522†	-29557†
GAM	-635991	-315169	-317376	-59212	-29562	-29573
GAM-S	-635454	-315285	-317086	-58549	-29277	-29287
GAMLSS	-624191	-309765	-311138	-57494	-28715	-28728
GAMLSS-S	*-623189*	*-309534*	*-310788*	*-56876*	*-28476*	*-28533*
CDR	-635180	-316198	-315934	-59006	-29447	-29484
CDR-NN base	-608836	-306662	**-308434**	-54552	-27509	**-27551**
–Nonlinear	-615024	-306674	—	-55768	-27947	—
–Nonstationary	-608761	-305773	—	-54602	***-27468***	—
–Heteroscedastic	-634122	-313797	—	-58199	-29067	—
+RNN	-597059	-307338	—	-54365	-27494	—
Units $\div$ 2 (16)	-609577	-306199	—	-55064	-27576	—
Units $\times$ 2 (64)	-599103	-308324	—	-54837	-27556	—
Layers - 1 (1)	-609947	-306182	—	-54984	-27718	—
Layers + 1 (3)	-608017	-305609	—	-54953	-27578	—
Weight Reg $\div$ 5 (1)	***-600313***	-308170	—	***-53776***	-27514	—
Weight Reg $\times$ 5 (25)	-610347	-305665	—	-55389	-27752	—
Ranef Reg $\div$ 10 (1)	-605305	-306491	—	-54180	-27540	—
Ranef Reg $\times$ 10 (100)	-606665	-305867	—	-54793	-27600	—
Dropout $\div$ 2 (0.05)	-600600	-308092	—	-54111	-27503	—
Dropout $\times$ 2 (0.2)	-612388	-305737	—	-55724	-27842	—
Learning Rate $\div$ 3 (0.001)	-605416	-308270	—	-54534	-27526	—
Learning Rate $\times$ 3 (0.009)	-609569	***-305508***	—	-55052	-27638	—
Batch Size $\div$ 2 (512)	-606452	-306474	—	-54796	-27547	—
Batch Size $\times$ 2 (2048)	-605107	-306633	—	-54729	-27597	—

Table S3: Dundee (go-past duration likelihood). Log likelihood from CDR-NN vs. linear mixed-effects (LME), generalized additive model (GAM), and generalized additive model for location, scale, and shape (GAMLSS) baselines with and without three additional spillover positions (-S) to help capture delayed effects, as well as kernel-based (non-neural) CDR (LME, GAM, and CDR performance as reported in Shain and Schuler 2021). LME baselines show the marginal likelihood for the training set (the default likelihood implemented by the `lme4` package). All other likelihoods are conditional on the fitted model. CDR-NN variants add recurrence (+RNN) and modify the number of Units (Units), number of hidden layers (Layers), weight regularization strength (Reg), random effects regularization strength (RanReg), dropout rate (Dropout), learning rate (LR), and batch size (Batch). Of the CDR-NN models, only CDR-NN base is evaluated on the test set. Best-performing models within the sets of baseline and CDR-NN models are shown in *italics*. Best-performing overall models are shown in **bold**. Daggers (†) indicate convergence failures.

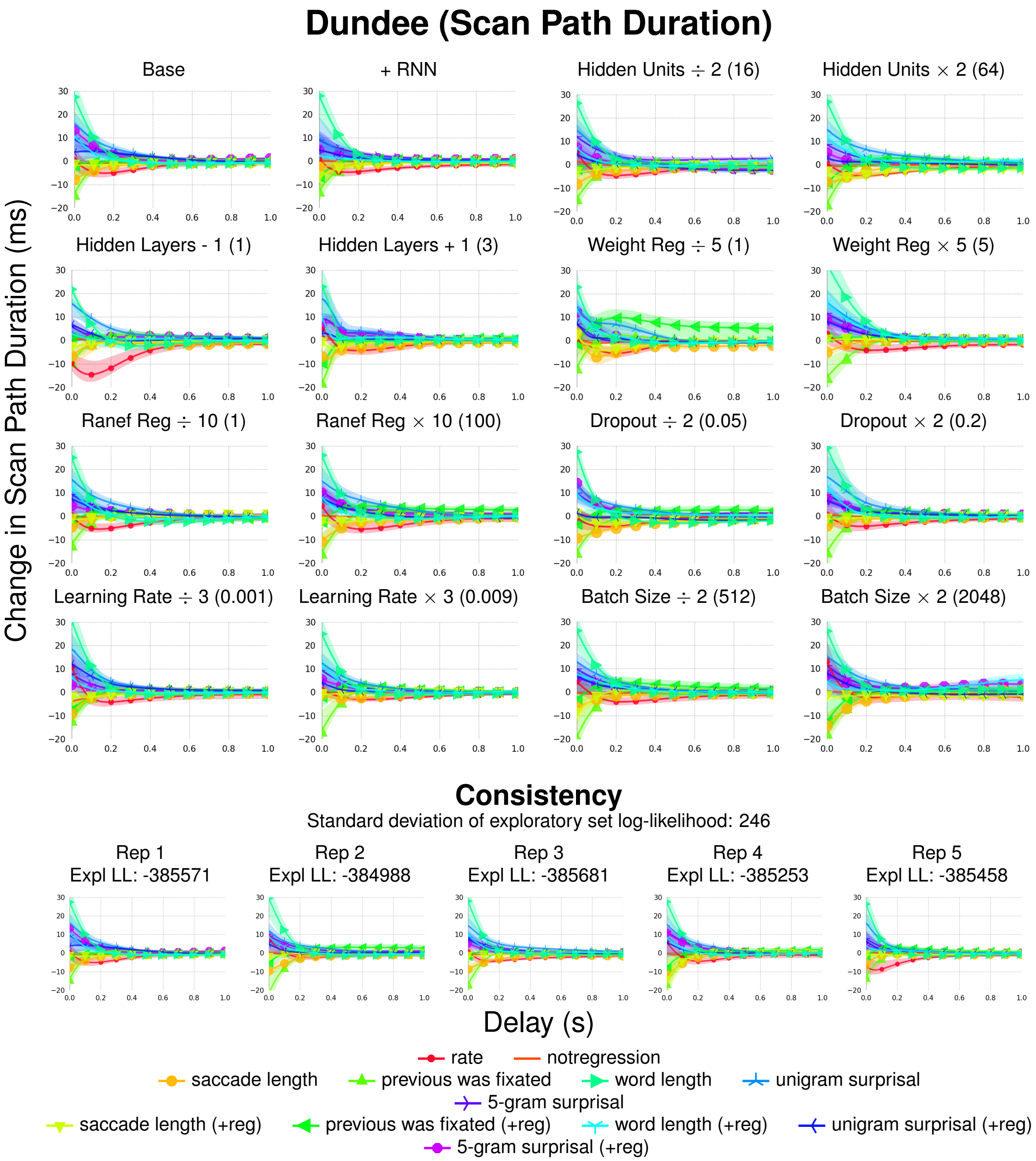

Figure S22: Dundee (scan path duration): Univariate CDR-NN IRF estimates from the Dundee eye-tracking corpus (scan path duration). Results using base hyperparameters are compared to estimates from models that deviate from the base in some dimension. Plots under "Consistency" show estimates from five replicates of the "base" configuration, where "Rep 1" is the same model as "base" above, replotted for ease of comparison.

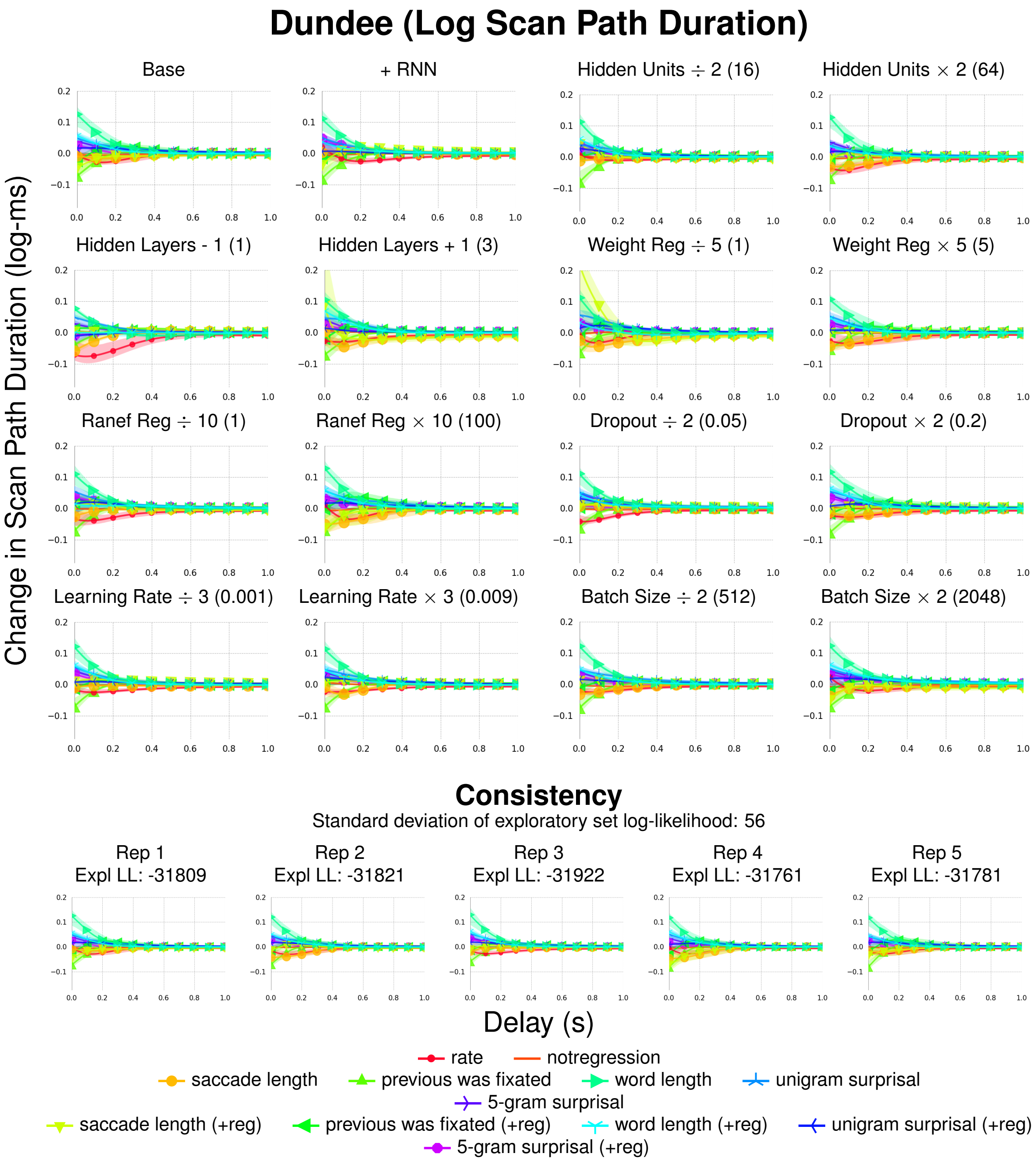

Figure S23: Dundee (log scan path duration): Univariate CDR-NN IRF estimates from the Dundee eye-tracking corpus (log-transformed scan path duration). Results using base hyperparameters are compared to estimates from models that deviate from the base in some dimension. Plots under "Consistency" show estimates from five replicates of the "base" configuration, where "Rep 1" is the same model as "base" above, replotted for ease of comparison.

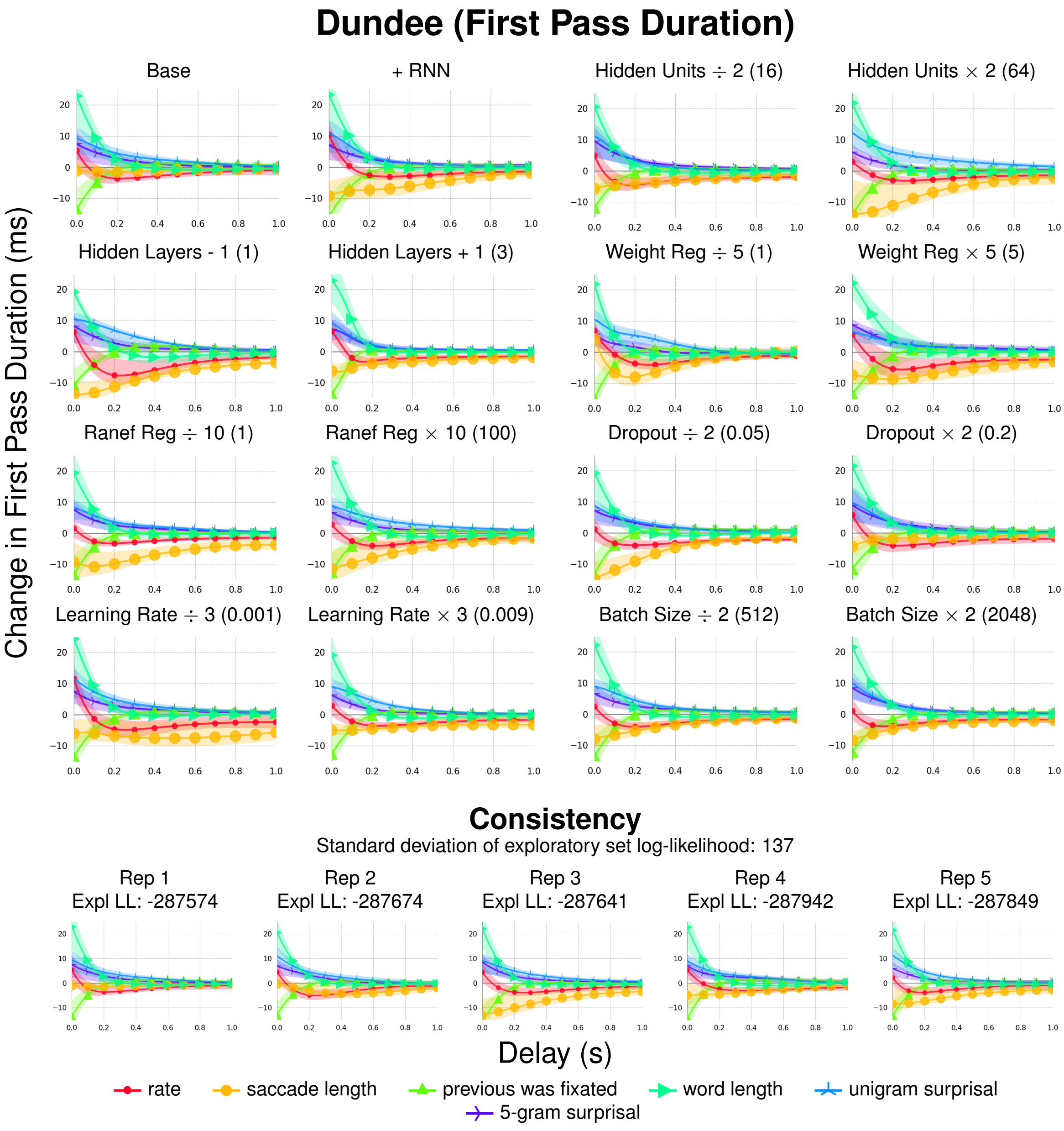

Figure S24: Dundee (first pass duration): Univariate CDR-NN IRF estimates from the Dundee eye-tracking corpus (first pass duration). Results using base hyperparameters are compared to estimates from models that deviate from the base in some dimension. Plots under “Consistency” show estimates from five replicates of the “base” configuration, where “Rep 1” is the same model as “base” above, replotted for ease of comparison.

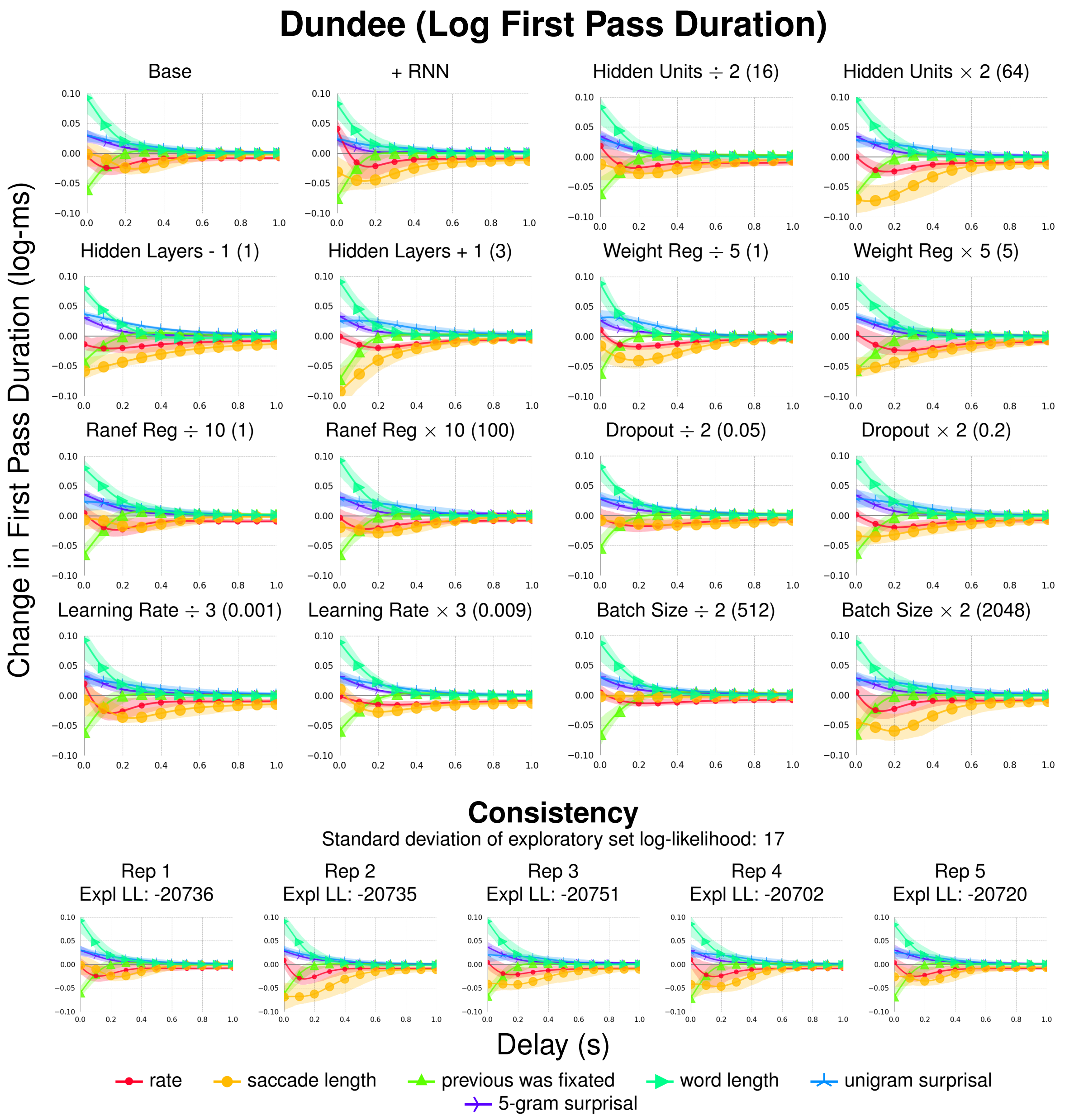

Figure S25: Dundee (log first pass duration): Univariate CDR-NN IRF estimates from the Dundee eye-tracking corpus (log-transformed first pass duration). Results using base hyperparameters are compared to estimates from models that deviate from the base in some dimension. Plots under "Consistency" show estimates from five replicates of the "base" configuration, where "Rep 1" is the same model as "base" above, replotted for ease of comparison.

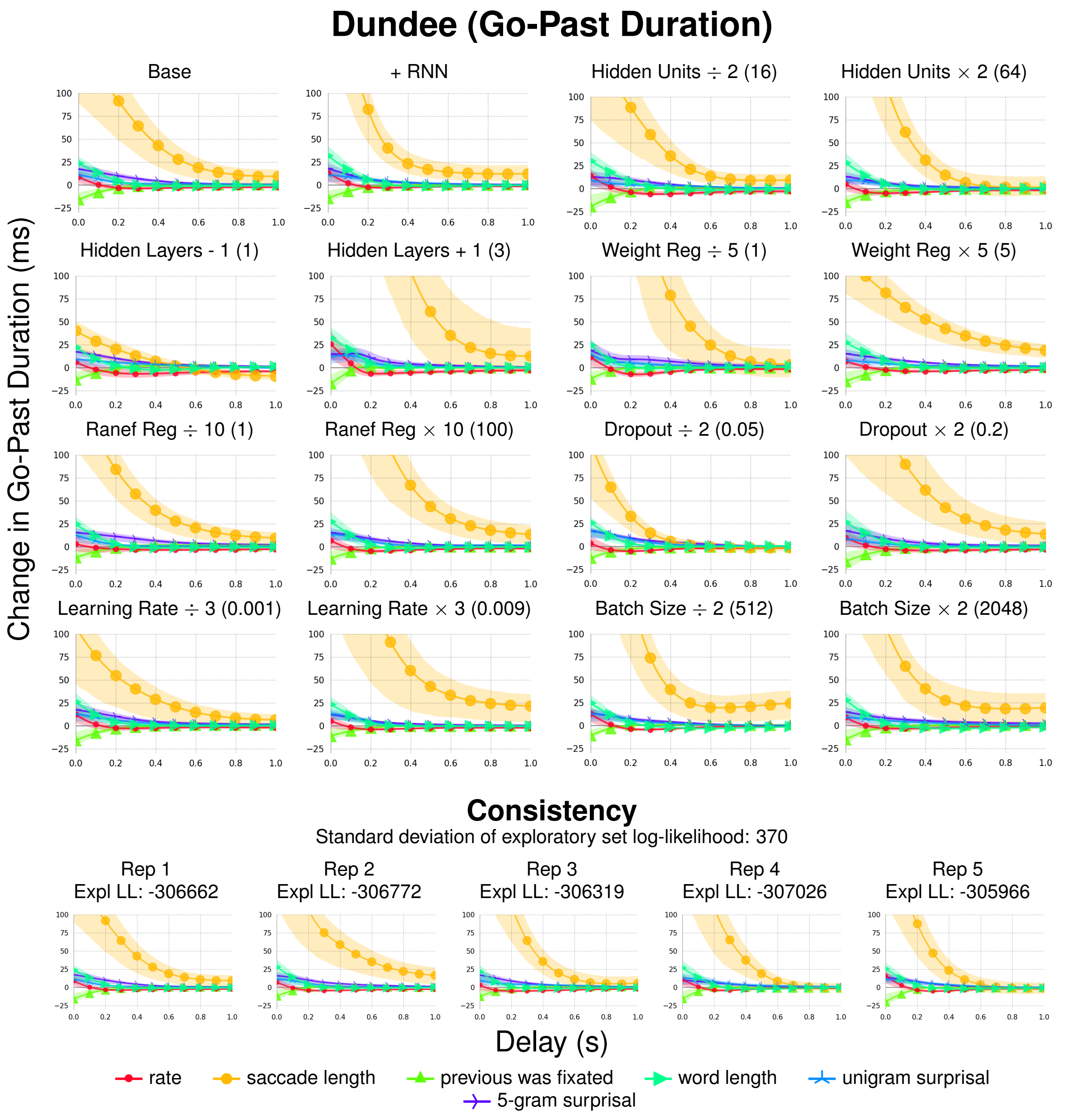

Figure S26: Dundee (go-past duration): Univariate CDR-NN IRF estimates from the Dundee eye-tracking corpus (go-past duration). Results using base hyperparameters are compared to estimates from models that deviate from the base in some dimension. Plots under “Consistency” show estimates from five replicates of the “base” configuration, where “Rep 1” is the same model as “base” above, replotted for ease of comparison. Some large estimates are clipped to preserve readability.

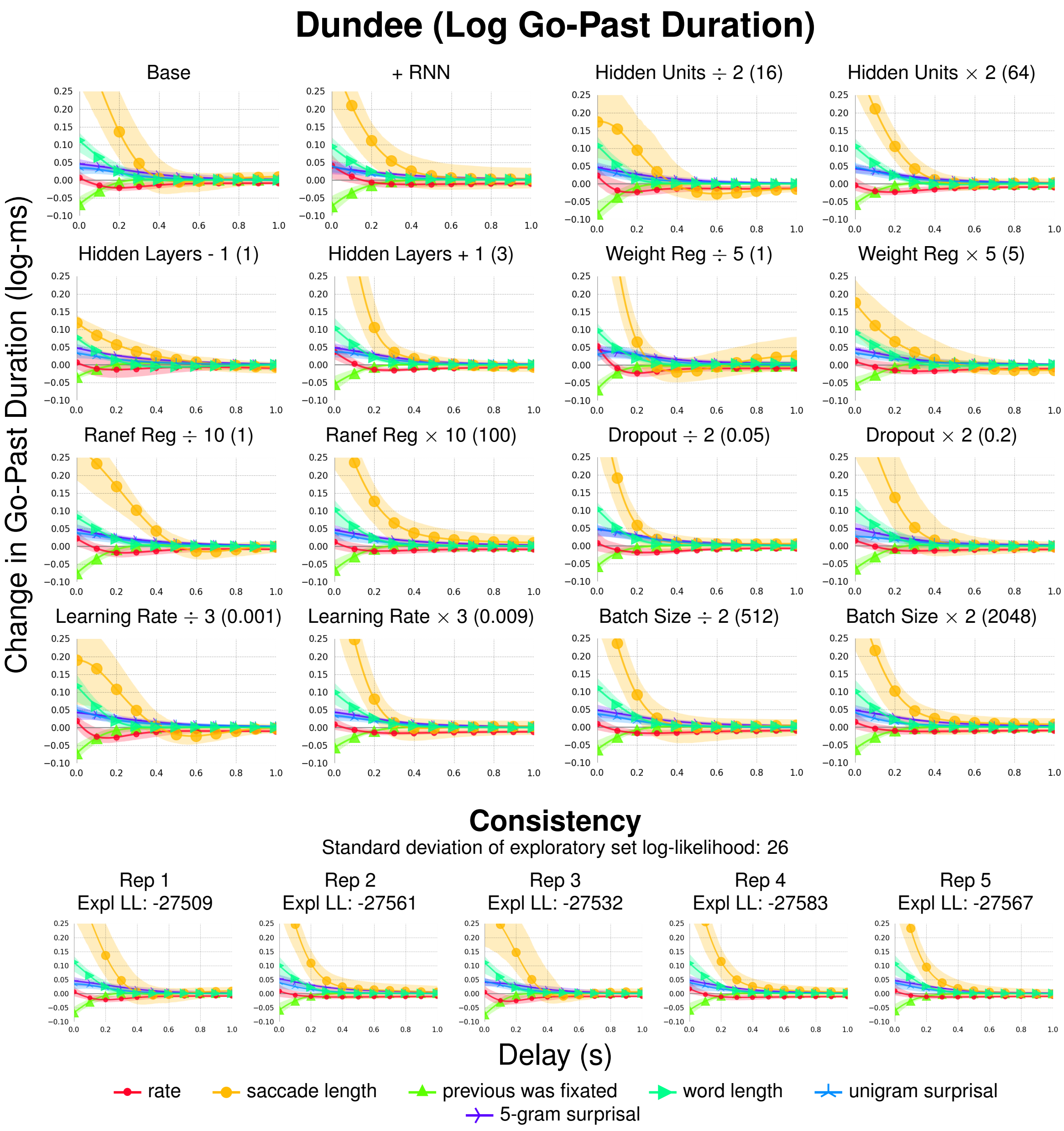

Figure S27: Dundee (log go-past duration): Univariate CDR-NN IRF estimates from the Dundee eye-tracking corpus (log-transformed go-past duration). Results using base hyperparameters are compared to estimates from models that deviate from the base in some dimension. Plots under “Consistency” show estimates from five replicates of the “base” configuration, where “Rep 1” is the same model as “base” above, replotted for ease of comparison. Some large estimates are clipped to preserve readability.

Model	Natural Stories Reading Time (ms) Train	Expl	Test	Natural Stories Reading Time (log ms) Train	Expl	Test
LME	-2444089	-1231466	-1224073	-62248	-31964	-31579
LME-S	-2442652†	-1230711†	-1223393†	-60232†	-30596†	-30220†
GAM	-2441347	-1230885	-1223532	-59305	-31454	-31004
GAM-S	-2439456	-1230163	-1222835	-55926	-30069	-29660
GAMLSS	-2386588	-1203461	-1196525	-39605	-21024	-20608
GAMLSS-S	*-2383131*	*-1202004*	*-1195295*	-37320	-19867	-19539
CDR	-2421724	-1220612	-1213359	*-18839*	*-10430*	*-10641*
CDR-NN base	-2263597	-1224555	**-1191747**	40237	10986	**12521**
–Nonlinear	-2275008	-1224562	—	36589	10515	—
–Nonstationary	-2266085	-1220449	—	36023	11530	—
–Heteroscedastic	-2414607	-1217627	—	-10311	-6838	—
+RNN	***-2206627***	-1364111	—	***49360***	-166401	—
Units ÷ 2 (16)	-2294417	-1208977	—	35453	12122	—
Units × 2 (64)	-2235490	-1264557	—	48125	8478	—
Layers - 1 (1)	-2304313	-1201513	—	30965	10535	—
Layers + 1 (3)	-2245651	-1237794	—	24978	220	—
Weight Reg ÷ 5 (1)	-2250353	-1209774	—	44075	12403	—
Weight Reg × 5 (25)	-2292479	-1226248	—	25585	7387	—
Ranef Reg ÷ 10 (1)	-2240188	-1247420	—	58557	7333	—
Ranef Reg × 10 (100)	-2260072	-1256309	—	40365	10835	—
Dropout ÷ 2 (0.05)	-2242987	-1240444	—	52666	10747	—
Dropout × 2 (0.2)	-2328802	-1190181	—	19053	7226	—
Learning Rate ÷ 3 (0.001)	-2261631	-1288272	—	44836	***12769***	—
Learning Rate × 3 (0.009)	-2288092	***-1189849***	—	32165	12346	—
Batch Size ÷ 2 (512)	-2276237	-1206397	—	37294	11336	—
Batch Size × 2 (2048)	-2256670	-1237186	—	42204	11307	—

Table S4: Natural Stories (reading time likelihood). Log likelihood from CDR-NN vs. linear mixed-effects (LME), generalized additive model (GAM), and generalized additive model for location, scale, and shape (GAMLSS) baselines with and without three additional spillover positions (-S) to help capture delayed effects, as well as kernel-based (non-neural) CDR (LME, GAM, and CDR performance as reported in Shain and Schuler 2021). LME baselines show the marginal likelihood for the training set (the default likelihood implemented by the `lme4` package). All other likelihoods are conditional on the fitted model. CDR-NN variants add recurrence (+RNN) and modify the number of Units (Units), number of hidden layers (Layers), weight regularization strength (Reg), random effects regularization strength (RanReg), dropout rate (Dropout), learning rate (LR), and batch size (Batch). Of the CDR-NN models, only CDR-NN base is evaluated on the test set. Best-performing models within the sets of baseline and CDR-NN models are shown in *italics*. Best-performing overall models are shown in **bold**. Daggers (†) indicate convergence failures.

H.2 Natural Stories (Self-Paced Reading)

In-sample and out-of-sample predictive performance on the Natural Stories SPR dataset is reported in **Supplementary Table S4**. Effect estimates in Natural Stories SPR are plotted in **Supplementary Figures S28–S29**. Estimates are plausible and highly consistent across model variants.

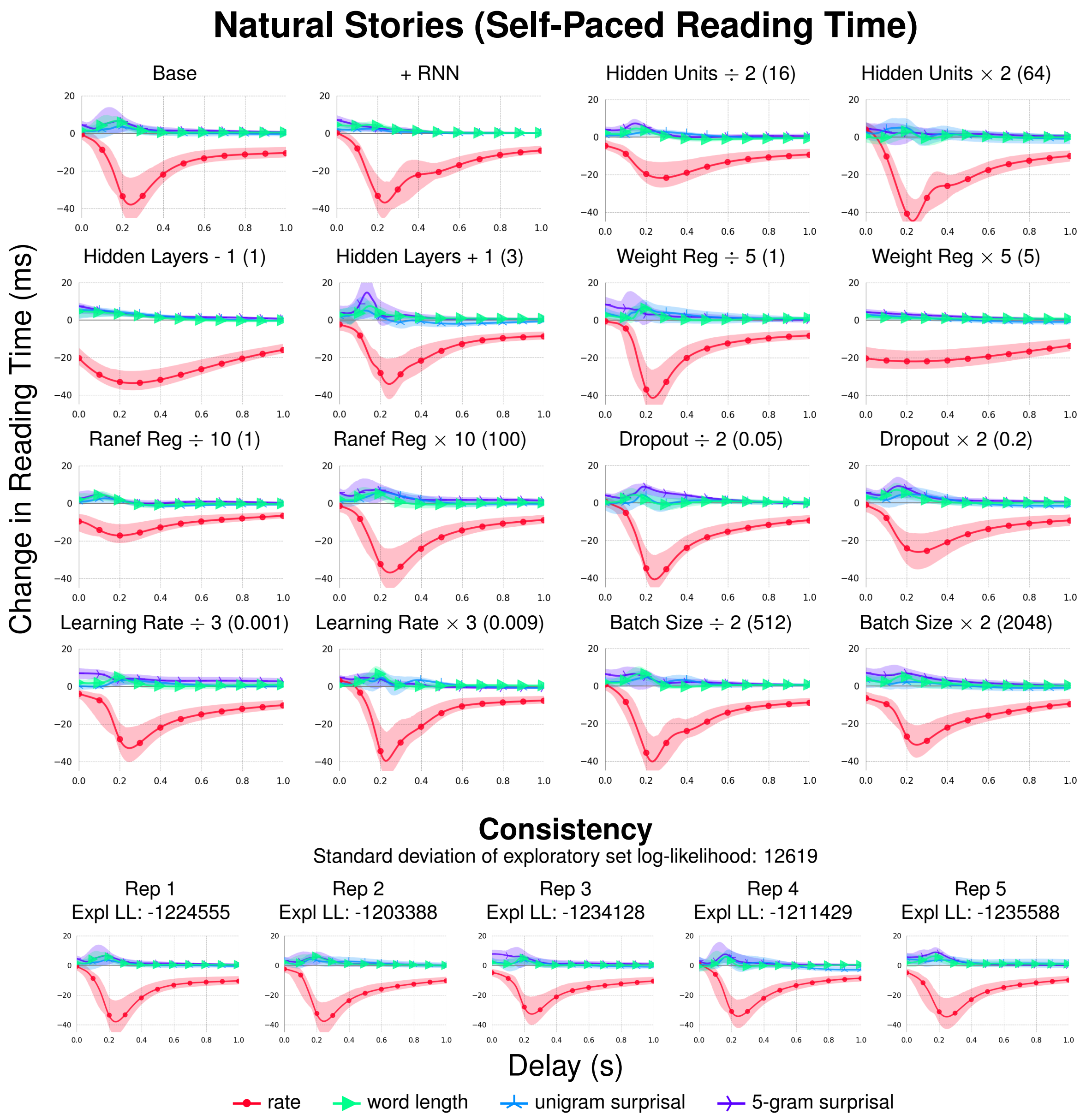

Figure S28: Natural Stories (reading time): Univariate CDR-NN IRF estimates from the Natural Stories self-paced reading corpus. Results using base hyperparameters are compared to estimates from models that deviate from the base in some dimension. Plots under "Consistency" show estimates from five replicates of the "base" configuration, where "Rep 1" is the same model as "base" above, replotted for ease of comparison.

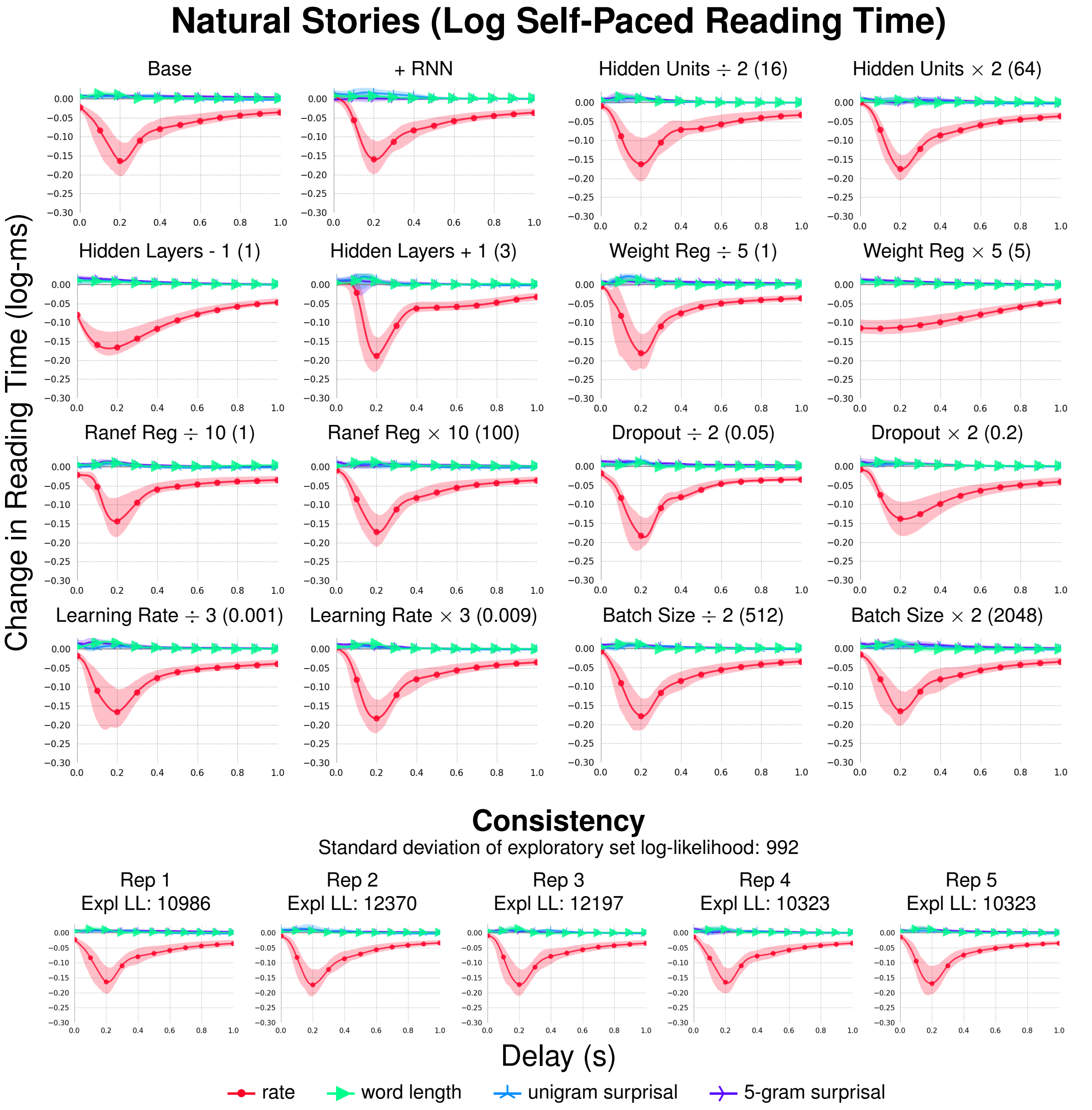

Figure S29: Natural Stories (log reading time): Univariate CDR-NN IRF estimates from the Natural Stories self-paced reading corpus (log transformed). Results using base hyperparameters are compared to estimates from models that deviate from the base in some dimension. Plots under "Consistency" show estimates from five replicates of the "base" configuration, where "Rep 1" is the same model as "base" above, replotted for ease of comparison.

Model	Natural Stories fMRI BOLD Train	Expl	Test
LME	-264358†	-137291†	-132788†
GAM	-263474	-137209	-132495
GAMLSS	*-253869*	*-131987*	*-127839*
CDR	-263889	-137012	-132730
CDR-NN base	-250013	-130579	**-126590**
–Nonlinear	-249531	-130140	—
–Nonstationary	-248835	***-129948***	—
–Heteroscedastic	-261503	-136442	—
+RNN	***-237930***	-160340	—
Units $\div$ 2 (16)	-250772	-130873	—
Units $\times$ 2 (64)	-247813	-129789	—
Layers - 1 (1)	-252764	-131610	—
Layers + 1 (3)	-249815	-130801	—
Weight Reg $\div$ 5 (1)	-249345	-130486	—
Weight Reg $\times$ 5 (25)	-253495	-131802	—
Ranef Reg $\times$ 10 (100)	-244538	-130107	—
Ranef Reg $\times$ 10 (100)00	-252097	-131434	—
Dropout $\div$ 2 (0.05)	-248638	-130129	—
Dropout $\times$ 2 (0.2)	-253291	-131724	—
Learning Rate $\div$ 3 (0.001)	-249397	-130629	—
Learning Rate $\times$ 3 (0.009)	-249432	-130120	—
Batch Size $\div$ 2 (512)	-248414	-129862	—
Batch Size $\times$ 2 (2048)	-251670	-131464	—

Table S5: Natural Stories (fMRI BOLD response in language-selective regions). Log likelihood from CDR-NN vs. linear mixed-effects (LME) fitted to fMRI data treated with different established preprocessing techniques, including pre-convolution with the canonical HRF, linear interpolation, averaging predictor values between acquisition times, and Lanczos interpolation, as well as generalized additive models (GAMs) and generalized additive models for location, scale, and shape (GAMLSS) fitted to data pre-convolved with the canonical HRF (LME and CDR performance as reported in Shain and Schuler 2021). LME baselines show the marginal likelihood for the training set (the default likelihood implemented by the `lme4` package). All other likelihoods are conditional on the fitted model. CDR-NN variants add recurrence (+RNN) and modify the number of Units (Units), number of hidden layers (Layers), weight regularization strength (Reg), random effects regularization strength (RanReg), dropout rate (Dropout), learning rate (LR), and batch size (Batch). Of the CDR-NN models, only CDR-NN base is evaluated on the test set. Best-performing models within the sets of baseline and CDR-NN models are shown in *italics*. Best-performing overall models are shown in **bold**. Daggers (†) indicate convergence failures.

H.3 Natural Stories (fMRI)

In-sample and out-of-sample predictive performance on the Natural Stories fMRI dataset is reported in **Supplementary Table S5**. Effect estimates in Natural Stories fMRI are plotted in **Supplementary Figure S30**. Estimates are plausible and broadly similar across model variants, although they vary in their details substantially more than the estimates for the reading datasets, likely due to higher levels of intrinsic noise in this domain.

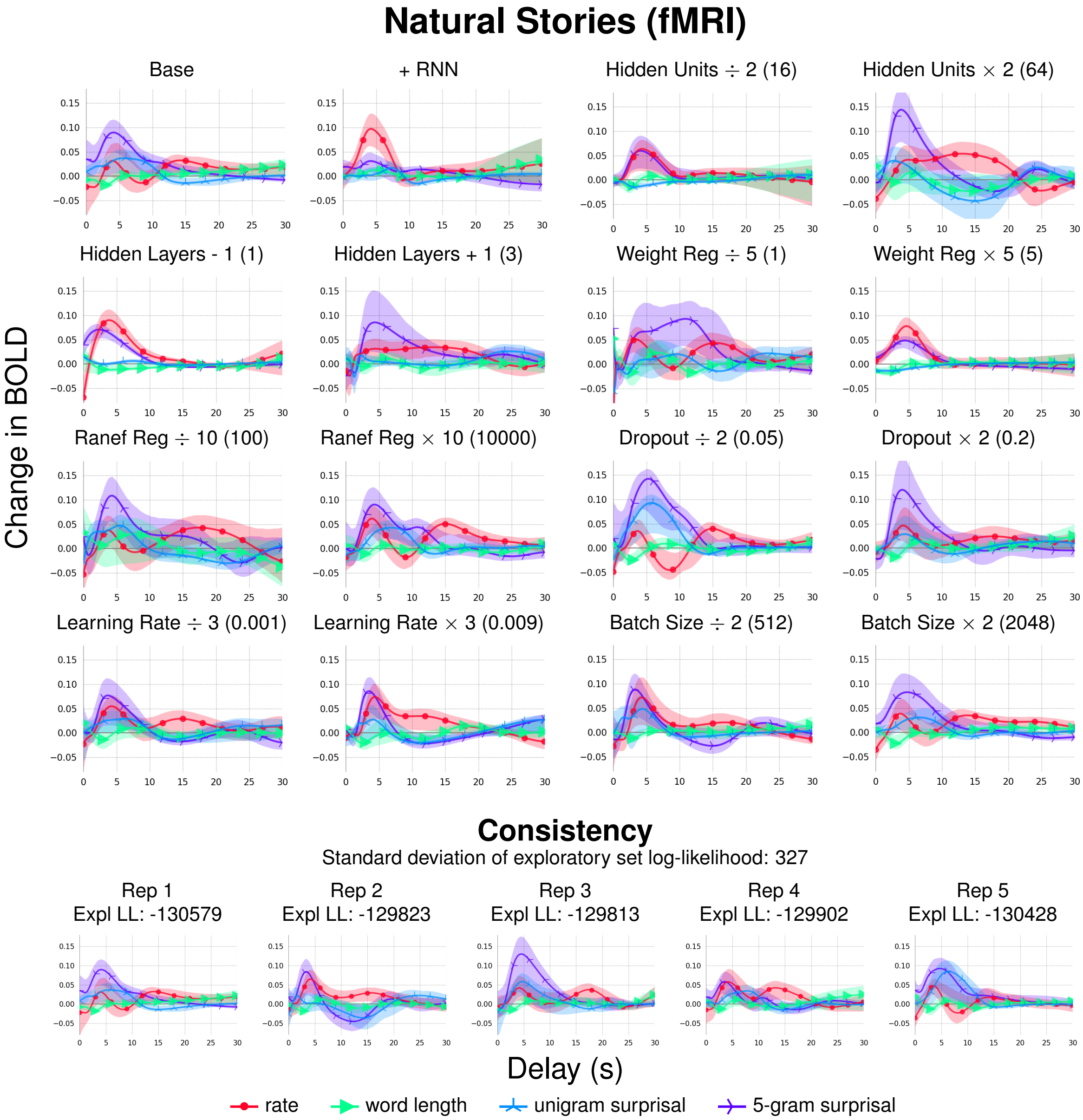

Figure S30: Natural Stories (fMRI): Univariate CDR-NN IRF estimates from the language-selective brain regions in the Natural Stories fMRI dataset. Results using base hyperparameters are compared to estimates from models that deviate from the base in some dimension. Plots under "Consistency" show estimates from five replicates of the "base" configuration, where "Rep 1" is the same model as "base" above, replotted for ease of comparison.

	Loglik							
	Train				Expl			
Dataset	Median	Min	Max	Spread	Median	Min	Max	Spread
Synth E0	-47624	-47671	-47532	139	-48869	-49000	-48792	208
Synth E1	-47609	-47673	-47470	203	-48867	-48963	-48791	172
Synth E10	-48076	-48133	-48023	110	-49385	-49512	-49311	201
Synth E100	-59908	-59968	-59750	218	-61233	-61333	-61148	185
Synth FSS	-52739	-52751	-52561	189	-53254	-53424	-53231	194
Synth FSL	-45813	-45888	-45720	168	-48691	-48830	-48615	215
Synth RSS	-52422	-52602	-52285	317	-54500	-54717	-54337	380
Synth RSL	-46954	-46991	-46707	285	-49574	-49817	-49415	402
Synth RAS	-51955	-51979	-51846	133	-53033	-53085	-52937	148
Synth RAL	-43984	-44023	-43821	202	-46732	-46855	-46657	198
Synth R0.00	-50621	-50695	-50540	155	-52434	-52640	-52396	243
Synth R0.25	-50100	-50192	-49999	192	-51456	-51647	-51403	244
Synth R0.50	-49548	-49597	-49470	126	-50183	-50214	-50110	104
Synth R0.75	-48820	-48969	-48741	228	-49447	-49461	-49352	109
Synth R0.90	-48613	-48640	-48463	178	-49033	-49085	-48949	136
Synth R0.95	-48487	-48492	-48422	71	-49230	-49248	-49221	27
Synth Exp	-56491	-56553	-56414	138	-57847	-57962	-57742	220
Synth Normal	-53332	-53374	-53160	214	-55208	-55366	-55090	277
Synth Gamma	-53004	-53147	-52939	2084	-54443	-54532	-54313	219
NatStor (RT)	-2257085	-2260266	-2254447	5819	-1215293	-1228864	-1201714	27150
NatStor (log RT)	44814	42910	46898	3988	11662	11021	13561	2540
Dundee (FP)	-564252	-565470	-563804	1666	-288257	-288433	-288071	362
Dundee (log FP)	-39696	-39740	-39438	302	-20735	-20750	-20709	41
Dundee (GP)	-602841	-603463	-601880	1583	-307875	-307936	-307280	656
Dundee (log GP)	-53825	-53889	-53628	261	-27556	-27586	-27474	112
Dundee (SP)	-756298	-756572	-755303	1269	-386310	-386896	-385588	1307
Dundee (log SP)	-61205	-61381	-61038	343	-31834	-31914	-31740	174
fMRI	-248687	-249617	-247269	2348	-130094	-130470	-129692	777

Table S6: Consistency of Performance Across Replicates. Training and exploratory set log likelihoods across 5 replicates of the base CDR-NN model for each response variable in the main analyses. The log likelihood statistic is high variance across replicates of the same model (typically spread over hundreds or even thousands of likelihood points), leading to the possibility that statistical differences in performance between models may be driven by optimization noise. For this reason, it is recommended (a) to statistically compare ensembles of models and (b) to use early stopping on a validation set to prevent overfitting.

I Full Results: Consistency

The full results for the model consistency analysis (variability of predictive performance across replicates of the CDR-NN base model definition) are presented in Table S6.

J Significance Testing through Model Comparison

J.1 General Approach

All statistical comparisons use paired permutation tests of out-of-sample conditional likelihood. The logic of the test is as follows: if null hypothesis H_0 is true, then a model instantiating alternative hypothesis H_1 should predict unseen data no better than a model instantiating H_0. The difference between H_1 and H_0 is cached out as a *constraint* that is enforced in H_0 but not in H_1, and the test asks whether relaxing this constraint leads to significantly improved fit, and thus, a basis for rejecting the null. The advantages of this approach are that (1) it requires no assumptions about the sampling distribution of effects in CDR-NNs, (2) it can be applied to any null hypothesis that can be instantiated as a model constraint, and (3) it is based directly on generalization performance, thereby potentially improving replicability of results.

As in CDR (Shain and Schuler, 2021), in-sample tests of CDR-NN models are discouraged: intervals-based tests are not necessarily valid because the intervals are variationally approximated, and in-sample tests like the likelihood ratio test are not necessarily valid because likelihood in a non-convex model like CDR-NN

cannot be guaranteed to be maximized. Even if it could, the model is so expressive that in-sample likelihood gains can simply be due to overfitting.

However, as discussed in **SI H**, variability in CDR-NNs' out-of-sample generalization performance across random seeds also presents a problem for null hypothesis significance testing via pairwise model comparison: performance differences can be driven by noise in the optimization process, in addition to any differences due to the constraint that distinguishes H_0 from H_1. This problem can be mitigated by comparing *ensembles* of models, where an ensemble is a set of E replicates of a given model design. To perform such a comparison, we generalize the Shain and Schuler (2021) paired permutation test to ensembles of models. Note that, given N evaluation items and corresponding sets of log-likelihoods $\mathcal{E}_0$ and $\mathcal{E}_1$ respectively derived from the E component models of the ensembles representing H_0 and H_1, the $2E$ likelihoods assigned to each datum in the evaluation set are exchangeable under the null hypothesis of no performance difference between models. We therefore compute a bootstrap sample in a way that respects these exchangeability criteria (see also e.g., Winkler et al. 2014, 2015) by randomly resampling ensembles by item, using the following procedure:

1. For each of the N evaluation items $1 \leq n \leq N$, repartition the $2E$ log-likelihood statistics into two random sets of likelihoods $\hat{\mathcal{E}}_{1,n}$, $\hat{\mathcal{E}}_{2,n}$, each with E elements.

2. Compute the resampled dataset likelihood as the sum of averages within the resampled partition: $\hat{\mathcal{L}}_i = \sum_{n=1}^{N} \frac{1}{E} \sum_{e=1}^{E} \hat{\mathcal{E}}_{i,n,e}$.

3. Compute and store the absolute difference $|\hat{\mathcal{L}}_1 - \hat{\mathcal{L}}_2|$.

This process is repeated many times to construct an empirical null distribution over the likelihood differences between ensembles, which is then compared to the observed difference in mean likelihood between ensembles in order to compute a p value. The number of resampling iterations determines the minimum obtainable p value. Here, we use 10,000 resampling iterations, and thus the minimum detectable p value is 0.0001. In cases where none of the resampled likelihood differences exceed the empirical one, this value serves as an upper bound on p.

Estimates from an entire ensemble are visualized by first sampling uniformly over the E fitted ensemble components, then sampling a model from that component's variational posterior, then querying the sampled model. Repeated many times, this procedure defines an empirical distribution over model estimates that takes into account uncertainty across the ensemble. All plots derived from ensembles were computed in this way. Unless otherwise stated, plots show the effect on the mean of the response distribution. All ensembles in this study used $E = 10$. Comparisons of CDR-NNs to baseline models do not involve ensembling, since most baselines are deterministically optimized. This is equivalent to using the procedure above with $E = 1$.

J.2 Null Models Used in this Study

Here we present the implementation details for the null models used to test effects in Examples A–E.

J.2.1 Example A: The Existence of Effects

To test for the presence of overall effects of a predictor, it is necessary to construct a null model in which the predictor is not present. To do so, we simply remove the predictor from the model altogether, holding everything else constant.

J.2.2 Example B: Linearity of Effects

To test nonlinearity of effects, it is necessary to construct a null model model in which the critical effect is constrained to be linear. To do so, we remove the predictor of interest from the inputs to the deep neural IRF (but retain it in the outputs), thereby enforcing a linear response (since the IRF cannot condition on the value of the predictor and therefore can only define a linear coefficient).

J.2.3 Example C: Effect Interactions

To test an interaction of predictors A and B, it is necessary to construct a null model in which both A and B are present but cannot interact. This is challenging to enforce within a single neural network, where interactions between inputs are ubiquitous in non-initial layers. To address this problem, we implement the IRF as a sum of the outputs of two distinct neural networks, one containing A along with all other predictors but B, and another containing B along with all other predictors but A. In this way, the interaction between A and B is removed while retaining all remaining interactions (i.e., between A and all other predictors but B, and between B and all other predictors but A). To ensure a minimal comparison, we reimplement the full model (containing the interaction between A and B) by keeping the same two-network IRF design, but including all predictors (A, B, and all other predictors) as inputs to each network. The IRF in the full model is thus the sum of two neural networks with identical inputs and architecture, but with different weights. This redundant design does not alter the solution space of the model, and it improves architectural parallelism between the full and ablated models for this comparison, reducing the likelihood of differences due to architecture (rather than the presence/absence of the critical interaction)..

J.2.4 Example D: Distributional Regression

To test an effect of a predictor on a specific distributional parameter, it is necessary to construct a null model in which only that effect is removed. To do so, we split the IRF into two distinct neural networks, one that generates μ, and another that generates σ. Doing so permits selective removal of effects in one but not the other distributional parameter. To ensure a minimal comparison, we reimplement the full model using this two-network design without ablating any effects on any distributional parameters.

J.2.5 Example E: Nonstationarity

To test nonstationarity of an effect, it is necessary to construct a null model in which the timestamp (which licenses nonstationarity) is removed from the input for the IRF to that effect only, retaining it for all others. To do so, we split the IRF into two distinct neural networks, one that retains the timestamp in the input and convolves all predictors but the critical one, and one that omits the timestamp but convolves only the critical predictor, while additionally including all remaining predictors as network *inputs* to ensure retention of interactions. This guarantees that the response to the critical predictor is stationary. To ensure a minimal comparison, we reimplement the full model using this two-network design without ablating the timestamp in either network.

K LME, GAM, GAMLSS, and CDR Baseline Model Specifications

For ease of reference, here we present the model formulae used to define the LME (`lme4` package), GAM (`mgcv` package), and GAMLSS (`gamlss` package) baseline models in R as well as the non-neural CDR (`cdr` package) baseline models in Python. For full software implementation details, see the public code repository at https://github.com/coryshain/cdr. Variable names below are modified from those used in our codebase for readability. Variable names suffixed with Sn (e.g., S2) are "lagged", representing that variable's value from n timesteps into the past. In all GAMLSS models, the same formula was used simultaneously for the μ and σ parameters. GAM models require at least as many unique values for a predictor as there are knots in its associated smoothing spline. As a result, the number of knots k is assigned manually for variables with too few unique values. For example, as discussed in **Materials and Methods: Datasets**, the Dundee dataset was filtered to remove values of *SaccadeLength* > 4. As a result, *SaccadeLength* has only 4 unique values, and k is manually set to 4 in the relevant formulae below.

K.1 Dundee (Scan Path)

K.1.1 LME (0 lags)

y ∼ Trial + SentencePosition + NotARegression + SaccadeLength + SaccadeLengthInRegression + PreviousWasFixated + PreviousWasFixatedInRegression + WordLength + WordLengthInRegression + UnigramSurprisal + UnigramSurprisalInRegression + 5GramSurprisal + 5GramSurprisalInRegression + (1 + Trial + SentencePosition + NotARegression + SaccadeLength + SaccadeLengthInRegression + PreviousWasFixated + PreviousWasFixatedInRegression + WordLength + WordLengthInRegression + UnigramSurprisal + UnigramSurprisalInRegression + 5GramSurprisal + 5GramSurprisalInRegression | Participant)

K.1.2 LME (3 lags)

y ∼ Trial + SentencePosition + NotARegression + NotARegressionS1 + NotARegressionS2 + NotARegressionS3 + SaccadeLength + SaccadeLengthInRegression + SaccadeLengthS1 + SaccadeLengthInRegressionS1 + SaccadeLengthS2 + SaccadeLengthInRegressionS2 + SaccadeLengthS3 + SaccadeLengthInRegressionS3 + PreviousWasFixated + PreviousWasFixatedInRegression + PreviousWasFixatedS1 + PreviousWasFixatedInRegressionS1 + PreviousWasFixatedS2 + PreviousWasFixatedInRegressionS2 + PreviousWasFixatedS3 + PreviousWasFixatedInRegressionS3 + WordLength + WordLengthInRegression + WordLengthS1 + WordLengthInRegressionS1 + WordLengthS2 + WordLengthInRegressionS2 + WordLengthS3 + WordLengthInRegressionS3 + UnigramSurprisal + UnigramSurprisalInRegression + UnigramSurprisalS1 + UnigramSurprisalInRegressionS1 + UnigramSurprisalS2 + UnigramSurprisalInRegressionS2 + UnigramSurprisalS3 + UnigramSurprisalInRegressionS3 + 5GramSurprisal + 5GramSurprisalInRegression + 5GramSurprisalS1 + 5GramSurprisalInRegressionS1 + 5GramSurprisalS2 + 5GramSurprisalInRegressionS2 + 5GramSurprisalS3 + 5GramSurprisalInRegressionS3 + (1 + Trial + SentencePosition + NotARegression + NotARegressionS1 + NotARegressionS2 + NotARegressionS3 + SaccadeLength + SaccadeLengthInRegression + SaccadeLengthS1 + SaccadeLengthInRegressionS1 + SaccadeLengthS2 + SaccadeLengthInRegressionS2 + SaccadeLengthS3 + SaccadeLengthInRegressionS3 + PreviousWasFixated + PreviousWasFixatedInRegression + PreviousWasFixatedS1 + PreviousWasFixatedInRegressionS1 + PreviousWasFixatedS2 + PreviousWasFixatedInRegressionS2 + PreviousWasFixatedS3 + PreviousWasFixatedInRegressionS3 + WordLength + WordLengthInRegression + WordLengthS1 + WordLengthInRegressionS1 + WordLengthS2 + WordLengthInRegressionS2 + WordLengthS3 + WordLengthInRegressionS3 + UnigramSurprisal + UnigramSurprisalInRegression + UnigramSurprisalS1 + UnigramSurprisalInRegressionS1 + UnigramSurprisalS2 + UnigramSurprisalInRegressionS2 + UnigramSurprisalS3 + UnigramSurprisalInRegressionS3 + 5GramSurprisal + 5GramSurprisalInRegression + 5GramSurprisalS1 + 5GramSurprisalInRegressionS1 + 5GramSurprisalS2 + 5GramSurprisalInRegressionS2 + 5GramSurprisalS3 + 5GramSurprisalInRegressionS3 | Participant)

K.1.3 GAM (0 lags)

y ∼ s(Trial) + s(SentencePosition) + NotARegression + s(SaccadeLength, k=4) + s(SaccadeLengthInRegression, k=4) + PreviousWasFixated + PreviousWasFixatedInRegression + s(WordLength) + s(WordLengthInRegression) + s(UnigramSurprisal) + s(UnigramSurprisalInRegression) + s(5GramSurprisal) + s(5GramSurprisalInRegression) + s(Participant, bs="re") + s(Trial, Participant, bs="re") + s(SentencePosition, Participant, bs="re") + s(NotARegression, Participant, bs="re") + s(SaccadeLength, Participant, bs="re") + s(SaccadeLengthInRegression, Participant, bs="re") + s(PreviousWasFixated, Participant, bs="re") + s(PreviousWasFixatedInRegression, Participant, bs="re") + s(WordLength, Participant, bs="re") + s(WordLengthInRegression, Participant, bs="re") + s(UnigramSurprisal, Participant, bs="re") + s(UnigramSurprisalInRegression, Participant, bs="re") + s(5GramSurprisal, Participant, bs="re") + s(5GramSurprisalInRegression, Participant, bs="re")

K.1.4 GAM (3 lags)

y $\sim$ s(Trial, bs=‘fs’) + s(SentencePosition, bs=‘fs’) + NotARegression + NotARegressionS1 + NotARegressionS2 + NotARegressionS3 + s(SaccadeLength, k=4) + s(SaccadeLengthInRegression, k=4) + s(SaccadeLengthS1, k=4) + s(SaccadeLengthInRegressionS1, k=4) + s(SaccadeLengthS2, k=4) + s(SaccadeLengthInRegressionS2, k=4) + s(SaccadeLengthS3, k=4) + s(SaccadeLengthInRegressionS3, k=4) + PreviousWasFixated + PreviousWasFixatedInRegression + PreviousWasFixatedS1 + PreviousWasFixatedInRegressionS1 + PreviousWasFixatedS2 + PreviousWasFixatedInRegressionS2 + PreviousWasFixatedS3 + PreviousWasFixatedInRegressionS3 + s(WordLength) + s(WordLengthInRegression) + s(WordLengthS1) + s(WordLengthInRegressionS1) + s(WordLengthS2) + s(WordLengthInRegressionS2) + s(WordLengthS3) + s(WordLengthInRegressionS3) + s(UnigramSurprisal) + s(UnigramSurprisalInRegression) + s(UnigramSurprisalS1) + s(UnigramSurprisalInRegressionS1) + s(UnigramSurprisalS2) + s(UnigramSurprisalInRegressionS2) + s(UnigramSurprisalS3) + s(UnigramSurprisalInRegressionS3) + s(5GramSurprisal) + s(5GramSurprisalInRegression) + s(5GramSurprisalS1) + s(5GramSurprisalInRegressionS1) + s(5GramSurprisalS2) + s(5GramSurprisalInRegressionS2) + s(5GramSurprisalS3) + s(5GramSurprisalInRegressionS3) + s(Participant, bs=“re”) + s(Trial, Participant, bs=“re”) + s(SentencePosition, Participant, bs=“re”) + s(NotARegression, Participant, bs=“re”) + s(NotARegressionS1, Participant, bs=“re”) + s(NotARegressionS2, Participant, bs=“re”) + s(NotARegressionS3, Participant, bs=“re”) + s(SaccadeLength, Participant, bs=“re”) + s(SaccadeLengthInRegression, Participant, bs=“re”) + s(SaccadeLengthS1, Participant, bs=“re”) + s(SaccadeLengthInRegressionS1, Participant, bs=“re”) + s(SaccadeLengthS2, Participant, bs=“re”) + s(SaccadeLengthInRegressionS2, Participant, bs=“re”) + s(SaccadeLengthS3, Participant, bs=“re”) + s(SaccadeLengthInRegressionS3, Participant, bs=“re”) + s(PreviousWasFixated, Participant, bs=“re”) + s(PreviousWasFixatedInRegression, Participant, bs=“re”) + s(PreviousWasFixatedS1, Participant, bs=“re”) + s(PreviousWasFixatedInRegressionS1, Participant, bs=“re”) + s(PreviousWasFixatedS2, Participant, bs=“re”) + s(PreviousWasFixatedInRegressionS2, Participant, bs=“re”) + s(PreviousWasFixatedS3, Participant, bs=“re”) + s(PreviousWasFixatedInRegressionS3, Participant, bs=“re”) + s(WordLength, Participant, bs=“re”) + s(WordLengthInRegression, Participant, bs=“re”) + s(WordLengthS1, Participant, bs=“re”) + s(WordLengthInRegressionS1, Participant, bs=“re”) + s(WordLengthS2, Participant, bs=“re”) + s(WordLengthInRegressionS2, Participant, bs=“re”) + s(WordLengthS3, Participant, bs=“re”) + s(WordLengthInRegressionS3, Participant, bs=“re”) + s(UnigramSurprisal, Participant, bs=“re”) + s(UnigramSurprisalInRegression, Participant, bs=“re”) + s(UnigramSurprisalS1, Participant, bs=“re”) + s(UnigramSurprisalInRegressionS1, Participant, bs=“re”) + s(UnigramSurprisalS2, Participant, bs=“re”) + s(UnigramSurprisalInRegressionS2, Participant, bs=“re”) + s(UnigramSurprisalS3, Participant, bs=“re”) + s(UnigramSurprisalInRegressionS3, Participant, bs=“re”) + s(5GramSurprisal, Participant, bs=“re”) + s(5GramSurprisalInRegression, Participant, bs=“re”) + s(5GramSurprisalS1, Participant, bs=“re”) + s(5GramSurprisalInRegressionS1, Participant, bs=“re”) + s(5GramSurprisalS2, Participant, bs=“re”) + s(5GramSurprisalInRegressionS2, Participant, bs=“re”) + s(5GramSurprisalS3, Participant, bs=“re”) + s(5GramSurprisalInRegressionS3, Participant, bs=“re”)

K.1.5 GAMLSS (0 lags)

y $\sim$ pb(Trial) + pb(SentencePosition) + NotARegression + SaccadeLength + SaccadeLengthInRegression + PreviousWasFixated + PreviousWasFixatedInRegression + pb(WordLength) + pb(WordLengthInRegression) + pb(UnigramSurprisal) + pb(UnigramSurprisalInRegression) + pb(5GramSurprisal) + pb(5GramSurprisalInRegression) + random(Participant)

K.1.6 GAMLSS (3 lags)

y $\sim$ pb(Trial) + pb(SentencePosition) + NotARegression + NotARegressionS1 + NotARegressionS2 + NotARegressionS3 + SaccadeLength + SaccadeLengthInRegression + SaccadeLengthS1 +

SaccadeLengthInRegressionS1 + SaccadeLengthS2 + SaccadeLengthInRegressionS2 + SaccadeLengthS3 + SaccadeLengthInRegressionS3 + PreviousWasFixated + PreviousWasFixatedInRegression + PreviousWasFixatedS1 + PreviousWasFixatedInRegressionS1 + PreviousWasFixatedS2 + PreviousWasFixatedInRegressionS2 + PreviousWasFixatedS3 + PreviousWasFixatedInRegressionS3 + pb(WordLength) + pb(WordLengthInRegression) + pb(WordLengthS1) + pb(WordLengthInRegressionS1) + pb(WordLengthS2) + pb(WordLengthInRegressionS2) + pb(WordLengthS3) + pb(WordLengthInRegressionS3) + pb(UnigramSurprisal) + pb(UnigramSurprisalInRegression) + pb(UnigramSurprisalS1) + pb(UnigramSurprisalInRegressionS1) + pb(UnigramSurprisalS2) + pb(UnigramSurprisalInRegressionS2) + pb(UnigramSurprisalS3) + pb(UnigramSurprisalInRegressionS3) + pb(5GramSurprisal) + pb(5GramSurprisalInRegression) + pb(5GramSurprisalS1) + pb(5GramSurprisalInRegressionS1) + pb(5GramSurprisalS2) + pb(5GramSurprisalInRegressionS2) + pb(5GramSurprisalS3) + pb(5GramSurprisalInRegressionS3) + random(Participant)

K.1.7 CDR

y $\sim$ Trial + SentencePosition + C(Rate + NotARegression + SaccadeLength + SaccadeLengthInRegression + PreviousWasFixated + PreviousWasFixatedInRegression + WordLength + WordLengthInRegression + UnigramSurprisal + UnigramSurprisalInRegression + 5GramSurprisal + 5GramSurprisalInRegression, ShiftedGammaShapeGT1(alpha=2, beta=2, delta=-0.5)) + (Trial + SentencePosition + C(Rate + NotARegression + SaccadeLength + SaccadeLengthInRegression + PreviousWasFixated + PreviousWasFixatedInRegression + WordLength + WordLengthInRegression + UnigramSurprisal + UnigramSurprisalInRegression + 5GramSurprisal + 5GramSurprisalInRegression, ShiftedGammaShapeGT1(alpha=2, beta=2, delta=-0.5, ran=T)) | Participant)

K.2 Dundee (First Pass and Go-Past)

K.2.1 LME (0 lags)

y $\sim$ Trial + SentencePosition + SaccadeLength + PreviousWasFixated + WordLength + UnigramSurprisal + 5GramSurprisal + (1 + Trial + SentencePosition + SaccadeLength + PreviousWasFixated + WordLength + UnigramSurprisal + 5GramSurprisal | Participant)

K.2.2 LME (3 lags)

y $\sim$ Trial + SentencePosition + SaccadeLength + SaccadeLengthS1 + SaccadeLengthS2 + SaccadeLengthS3 + PreviousWasFixated + PreviousWasFixatedS1 + PreviousWasFixatedS2 + PreviousWasFixatedS3 + WordLength + WordLengthS1 + WordLengthS2 + WordLengthS3 + UnigramSurprisal + UnigramSurprisalS1 + UnigramSurprisalS2 + UnigramSurprisalS3 + 5GramSurprisal + 5GramSurprisalS1 + 5GramSurprisalS2 + 5GramSurprisalS3 + (1 + Trial + SentencePosition + SaccadeLength + SaccadeLengthS1 + SaccadeLengthS2 + SaccadeLengthS3 + PreviousWasFixated + PreviousWasFixatedS1 + PreviousWasFixatedS2 + PreviousWasFixatedS3 + WordLength + WordLengthS1 + WordLengthS2 + WordLengthS3 + UnigramSurprisal + UnigramSurprisalS1 + UnigramSurprisalS2 + UnigramSurprisalS3 + 5GramSurprisal + 5GramSurprisalS1 + 5GramSurprisalS2 + 5GramSurprisalS3 | Participant)

K.2.3 GAM (0 lags)

y $\sim$ s(Trial) + s(SentencePosition) + s(SaccadeLength, k=4) + PreviousWasFixated + s(WordLength) + s(UnigramSurprisal) + s(5GramSurprisal) + s(Participant, bs="re") + s(Trial, Participant, bs="re") + s(SentencePosition, Participant, bs="re") + s(SaccadeLength, Participant, bs="re") + s(PreviousWasFixated, Participant, bs="re") + s(WordLength, Participant, bs="re") + s(UnigramSurprisal, Participant, bs="re") + s(5GramSurprisal, Participant, bs="re")

K.2.4 GAM (3 lags)

y $\sim$ s(Trial) + s(SentencePosition) + s(SaccadeLength, k=4) + s(SaccadeLengthS1, k=4) + s(SaccadeLengthS2, k=4) + s(SaccadeLengthS3, k=4) + PreviousWasFixated + PreviousWasFixatedS1 + PreviousWasFixatedS2 + PreviousWasFixatedS3 + s(WordLength) + s(WordLengthS1) + s(WordLengthS2) + s(WordLengthS3) + s(UnigramSurprisal) + s(UnigramSurprisalS1) + s(UnigramSurprisalS2) + s(UnigramSurprisalS3) + s(5GramSurprisal) + s(5GramSurprisalS1) + s(5GramSurprisalS2) + s(5GramSurprisalS3) + s(Participant, bs="re") + s(Trial, Participant, bs="re") + s(SentencePosition, Participant, bs="re") + s(SaccadeLength, Participant, bs="re") + s(SaccadeLengthS1, Participant, bs="re") + s(SaccadeLengthS2, Participant, bs="re") + s(SaccadeLengthS3, Participant, bs="re") + s(PreviousWasFixated, Participant, bs="re") + s(PreviousWasFixatedS1, Participant, bs="re") + s(PreviousWasFixatedS2, Participant, bs="re") + s(PreviousWasFixatedS3, Participant, bs="re") + s(WordLength, Participant, bs="re") + s(WordLengthS1, Participant, bs="re") + s(WordLengthS2, Participant, bs="re") + s(WordLengthS3, Participant, bs="re") + s(UnigramSurprisal, Participant, bs="re") + s(UnigramSurprisalS1, Participant, bs="re") + s(UnigramSurprisalS2, Participant, bs="re") + s(UnigramSurprisalS3, Participant, bs="re") + s(5GramSurprisal, Participant, bs="re") + s(5GramSurprisalS1, Participant, bs="re") + s(5GramSurprisalS2, Participant, bs="re") + s(5GramSurprisalS3, Participant, bs="re")

K.2.5 GAMLSS (0 lags)

y $\sim$ pb(Trial) + pb(SentencePosition) + SaccadeLength + PreviousWasFixated + pb(WordLength) + pb(UnigramSurprisal) + pb(5GramSurprisal) + random(Participant)

K.2.6 GAMLSS (3 lags)

y $\sim$ pb(Trial) + pb(SentencePosition) + SaccadeLength + SaccadeLengthS1 + SaccadeLengthS2 + SaccadeLengthS3 + PreviousWasFixated + PreviousWasFixatedS1 + PreviousWasFixatedS2 + PreviousWasFixatedS3 + pb(WordLength) + pb(WordLengthS1) + pb(WordLengthS2) + pb(WordLengthS3) + pb(UnigramSurprisal) + pb(UnigramSurprisalS1) + pb(UnigramSurprisalS2) + pb(UnigramSurprisalS3) + pb(5GramSurprisal) + pb(5GramSurprisalS1) + pb(5GramSurprisalS2) + pb(5GramSurprisalS3) + random(Participant)

K.2.7 CDR

y $\sim$ Trial + SentencePosition + C(Rate + SaccadeLength + PreviousWasFixated + WordLength + UnigramSurprisal + 5GramSurprisal, ShiftedGammaShapeGT1(alpha=2, beta=2, delta=-0.5)) + (Trial + SentencePosition + C(Rate + SaccadeLength + PreviousWasFixated + WordLength + UnigramSurprisal + 5GramSurprisal, ShiftedGammaShapeGT1(ran=T)) | Participant)

K.3 Natural Stories Self-Paced Reading (Reading Time)

K.3.1 LME (0 lags)

y $\sim$ Trial + SentencePosition + WordLength + UnigramSurprisal + 5GramSurprisal + (1 + Trial + SentencePosition + WordLength + UnigramSurprisal + 5GramSurprisal | Participant)

K.3.2 LME (3 lags)

y $\sim$ Trial + SentencePosition + WordLength + WordLengthS1 + WordLengthS2 + WordLengthS3 + + UnigramSurprisal + UnigramSurprisalS1 + UnigramSurprisalS2 + UnigramSurprisalS3 + 5GramSurprisal + 5GramSurprisalS1 + 5GramSurprisalS2 + 5GramSurprisalS3 + (1 + Trial + SentencePosition + WordLength + WordLengthS1 + WordLengthS2 + WordLengthS3 + UnigramSurprisal + UnigramSurprisalS1 +

UnigramSurprisalS2 + UnigramSurprisalS3 + 5GramSurprisal + 5GramSurprisalS1 + 5GramSurprisalS2 + 5GramSurprisalS3 | Participant)

K.3.3 GAM (0 lags)

y ∼ s(Trial) + s(SentencePosition) + s(WordLength) + s(UnigramSurprisal) + s(5GramSurprisal) + s(Participant, bs="re") + s(Trial, Participant, bs="re") + s(SentencePosition, Participant, bs="re") + s(WordLength, Participant, bs="re") + s(UnigramSurprisal, Participant, bs="re") + s(5GramSurprisal, Participant, bs="re")

K.3.4 GAM (3 lags)

y ∼ s(Trial) + s(Trial) + s(SentencePosition) + s(WordLength) + s(WordLengthS1) + s(WordLengthS2) + s(WordLengthS3) + s(UnigramSurprisal) + s(UnigramSurprisalS1) + s(UnigramSurprisalS2) + s(UnigramSurprisalS3) + s(5GramSurprisal) + s(5GramSurprisalS1) + s(5GramSurprisalS2) + s(5GramSurprisalS3) + s(Participant, bs="re") + s(Trial, Participant, bs="re") + s(SentencePosition, Participant, bs="re") + s(WordLength, Participant, bs="re") + s(WordLengthS1, Participant, bs="re") + s(WordLengthS2, Participant, bs="re") + s(WordLengthS3, Participant, bs="re") + s(UnigramSurprisal, Participant, bs="re") + s(UnigramSurprisalS1, Participant, bs="re") + s(UnigramSurprisalS2, Participant, bs="re") + s(UnigramSurprisalS3, Participant, bs="re") + s(5GramSurprisal, Participant, bs="re") + s(5GramSurprisalS1, Participant, bs="re") + s(5GramSurprisalS2, Participant, bs="re") + s(5GramSurprisalS3, Participant, bs="re")

K.3.5 GAMLSS (0 lags)

y ∼ pb(Trial) + pb(SentencePosition) + pb(WordLength) + pb(UnigramSurprisal) + pb(5GramSurprisal) + re(random= 1|Participant) + random(Participant)

K.3.6 GAMLSS (3 lags)

y ∼ pb(Trial) + pb(SentencePosition) + pb(WordLength) + pb(WordLengthS1) + pb(WordLengthS2) + pb(WordLengthS3) + pb(UnigramSurprisal) + pb(UnigramSurprisalS1) + pb(UnigramSurprisalS2) + pb(UnigramSurprisalS3) + pb(5GramSurprisal) + pb(5GramSurprisalS1) + pb(5GramSurprisalS2) + pb(5GramSurprisalS3) + random(Participant)

K.3.7 CDR

y ∼ Trial + SentencePosition + C(Rate + WordLength + UnigramSurprisal + 5GramSurprisal, ShiftedGammaShapeGT1()) + (Trial + SentencePosition + C(Rate + WordLength + UnigramSurprisal + 5GramSurprisal, ShiftedGammaShapeGT1(ran=T)) | Participant)

K.4 Natural Stories fMRI (BOLD)

K.4.1 LME

y ∼ TR + Rate + SoundPower + UnigramSurprisal + 5GramSurprisal + (TR + Rate + SoundPower + UnigramSurprisal + 5GramSurprisal | — fROI) + (1 | Participant)

K.4.2 GAM

y ∼ s(TR) + s(Rate) + s(SoundPower) + s(UnigramSurprisal) + s(5GramSurprisal) + s(Participant, bs="re") + s(fROI, bs="re") + s(TR, fROI, bs="re") + s(Rate, fROI, bs="re") + s(SoundPower, fROI, bs="re") + s(UnigramSurprisal, fROI, bs="re") + s(5GramSurprisal, fROI, bs="re")

K.4.3 GAMLSS

y ~ pb(TR) + pb(Rate) + pb(SoundPower) + pb(UnigramSurprisal) + pb(5GramSurprisal) + random(Participant) + random(fROI)

K.4.4 CDR

y ~ TR + C(Rate + SoundPower + UnigramSurprisal + 5GramSurprisal, HRFDoubleGamma5(irf_id=HRF)) + (TR + C(Rate + SoundPower + UnigramSurprisal + 5GramSurprisal, HRFDoubleGamma5(irf_id=HRF, ran=T)) | fROI) + (1 | Participant)
